# Optimising antibiotic switching via forecasting of patient physiology

**DOI:** 10.1038/s41467-026-76715-w

**Published:** 2026-08-25

**Authors:** Magnus Ross, Nel Swanepoel, Akish Luintel, Emma McGuire, Ingemar J. Cox, Steve Harris, Vasileios Lampos

**Affiliations:** 1 Department of Computer Science, Centre for AI, UCL, UK; 2 Institute of Health Informatics, UCL, UK; 3 Department of Medical Microbiology, Infection Division, University College Hospital London, London, UK; 4 Department of Computer Science, University of Copenhagen, Copenhagen, Denmark

**Keywords:** Health care, Translational research, Applied microbiology, Information technology

## Abstract

Timely transition from intravenous to oral antibiotic therapy shortens hospital stays, reduces catheter-related infections, and lowers healthcare costs, yet one in five patients in England remain on intravenous antibiotics despite meeting switching criteria. Clinical decision support systems can improve switching rates, but approaches that learn from historical decisions reproduce the delays and inconsistencies of routine practice. We propose using neural processes to model vital sign trajectories probabilistically, predicting switch-readiness by comparing forecasts against clinical guidelines rather than learning from past actions, and ranking patients to prioritise clinical review. The design yields interpretable outputs, adapts to updated guidelines without retraining, and preserves clinical judgement. Validated on datasets from US intensive care (6333 encounters) and a UK academic hospital group (10,584 encounters), the system selects 2.2–3.2 × more relevant patients than random. We show that forecasting patient physiology offers a principled foundation for decision support in antibiotic stewardship.

## Abstract (web summary)

The authors present a decision support system that forecasts patients’ vital signs to identify those ready to switch from intravenous to oral antibiotics. Clinical criteria are applied to the forecasts to rank patients for stewardship review.

## Introduction

The timely transition of hospital patients from intravenous (IV) to oral antimicrobial therapy, known as the IV-to-oral switch (IVOS), benefits patients and hospitals^1^. Clinically, it can shorten hospital stays and reduce catheter-related bloodstream infections^2,3^; operationally, it can free nursing time and reduce costs^4^. Yet IV therapy often continues after an oral alternative would be safe. In England, 19% of patients remain on IV antibiotics despite meeting switching criteria^5^, and some are not switched at all^6^. IVOS depends on timely review of a changing clinical picture, but that review is easy to miss on busy wards. Senior review, especially around discharge planning, microbiology and stewardship input, typically pushes prescribing towards IVOS, but these are finite resources that cannot reach every eligible patient when they are ready, delaying the switch.

IVOS decisions have two linked components^7,8^. The first is clinical stability: whether the patient is improving and likely to remain stable, assessed using trends in vital signs (e.g., temperature, heart rate, blood pressure, respiratory rate, oxygen saturation) and, where available, laboratory markers. The second is oral suitability: whether oral therapy is appropriate and feasible, for example, that the patient can tolerate oral intake and that an appropriate oral agent is available. This paper targets clinical stability. Our task is to identify patients whose recent observations imply a high probability of staying within normal ranges in the near future, so they can be prioritised for review.

This problem has a practical constraint^9^. In England, the median hospital has around 550 general/acute/maternity inpatients at any time, and about one-third receive antibiotics. That volume exceeds the capacity of typical antimicrobial stewardship teams (infection specialists and specialist pharmacists). Decision support must therefore help them focus on the patients most likely to benefit from review. IVOS decisions also depend on information that is hard to capture reliably in structured EHR data (for example, surgical wound progress, source control plans, evolving imaging, or bedside assessments). The goal of a clinical decision support system (CDSS) is therefore not to automate IVOS, but to surface patients for expert review and discussion with the primary team.

CDSSs can increase switching rates and reduce IV therapy days. Prior rule-based systems have reduced IV use through alerts based on fixed criteria^10–13^ (see Supplementary Discussion for a detailed review). However, fixed rules struggle to assess whether a patient is improving rather than merely meeting thresholds at one point in time. AI-enabled methods that learn from historical switching behaviour face a deeper limitation: if the training signal is what clinicians did, then the best the model can do is reproduce that sub-optimal behaviour. It cannot reliably improve practice against a clinical standard^14,15^. This bias can be partly mitigated by careful study design, for example, by deriving model training data from a curated cohort of switching decisions deemed appropriate by experts. Our approach instead circumvents switch labels entirely by enhancing established rule-based IVOS criteria with probabilistic forecasts of patient physiology.

**Fig. 1:**
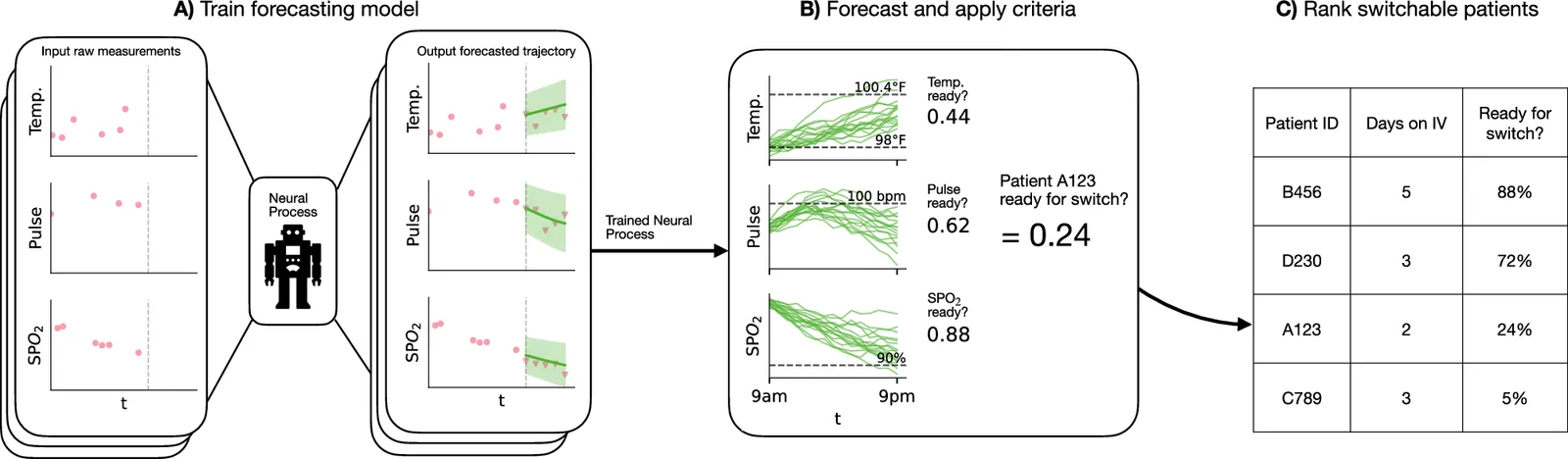
Overview of the proposed AI-enabled IVOS decision support system. **A** Training a probabilistic neural process model to forecast patient trajectories. **B** Using the trained model to generate likely trajectories over the following day and applying clinical criteria to determine the proportion of samples ready to be switched. **C** Presentation of switch-readiness probabilities as a ranked list of patients for review. Throughout, “switch-readiness” refers specifically to the physiological-stability component of the IVOS decision (vital signs within criteria); oral suitability and other clinical factors remain with the reviewing clinician.

In this work, we propose an alternative approach that forecasts patient physiology rather than past clinical actions. Using neural processes to model vital sign trajectories probabilistically, we predict switch-readiness by applying clinical criteria to these forecasts. This avoids learning historical delays, sidesteps the need to label switches from electronic health records (EHRs), and yields outputs clinicians can inspect directly (the forecast trajectories and their uncertainty). Because criteria are applied after forecasting, the system can be updated as clinical standards evolve without retraining the forecasting model, and it can be adapted in non-standard cases (e.g., discounting a variable that is clinically uninformative in context, such as fever in patients on antipyretics). The system ranks patients by predicted switch-readiness, helping teams prioritise reviews while preserving clinical judgement for factors outside routine vital signs data. Fig. 1 illustrates our system architecture. We forecast key vital signs (A), apply clinical criteria to estimate switch-readiness probabilities (B), and use these to rank patients for review (C). Oral suitability and the final IVOS decision remain with clinicians, who consider the broader clinical record and coordinate with the primary team after reviewing flagged patients^7,8^.

## Results

We validate our method using two distinct EHR datasets: MIMIC-IV v3.1^16^, comprising intensive care unit (ICU) admissions from Beth Israel Deaconess Medical Centre in Boston, USA, and a non-public dataset from University College London Hospitals (UCLH), a general hospital group in London, UK. These cohorts represent significantly different populations and clinical practices, providing a robust test of model generalisability (see Table 1 for detailed cohort characteristics).

**Table 1:** Comparison of the evaluation set cohorts for MIMIC-IV and UCLH datasets

Statistic	MIMIC	UCLH
Number of admissions Unique subjects Age (years) Length of stay (days)	6333 5850 65.70 ± 15.29 17.69 ± 17.07	10,584 8701 55.98 ± 19.24 14.23 ± 14.42
**Sex** Female Male	2579 (40.7%) 3754 (59.3%)	5564 (52.6%) 5019 (47.4%)
**Top 5 Race/Ethnicity**	White: 3772 (59.6%)	White British: 3324 (31.4%)
	Unknown: 1074 (17.0%)	Unknown: 3033 (28.7%)
	Black: 720 (11.4%)	Other White Bg.: 1204 (11.4%)
	Other: 292 (4.6%)	Other Ethnic Group: 671 (6.3%)
	Hispanic: 247 (3.9%)	Black African: 509 (4.8%)
**Top 5 Oral Antibiotics**	Azithromycin: 724 (11.4%)	Co-amoxiclav: 3471 (32.8%)
	Metronidazole: 704 (11.1%)	Clarithromycin: 797 (7.5%)
	Sulfameth/Trimeth: 477 (7.5%)	Ciprofloxacin: 771 (7.3%)
	Doxycycline: 371 (5.9%)	Amoxicillin: 421 (4.0%)
	Vancomycin: 370 (5.8%)	Doxycycline: 410 (3.9%)
**Top 5 IV Antibiotics**	Vancomycin: 4158 (65.7%)	Co-amoxiclav: 4920 (46.5%)
	Cefepime: 2774 (43.8%)	Pip.-Taz.: 3841 (36.3%)
	Ceftriaxone: 2629 (41.5%)	Metronidazole: 2849 (26.9%)
	Cefazolin: 2249 (35.5%)	Meropenem: 1208 (11.4%)
	Metronidazole: 1449 (22.9%)	Ciprofloxacin: 882 (8.3%)
**Measurement variable**	**Mean # per admission**	**Mean # per admission**
Heart rate Respiratory rate SpO_2_ Systolic BP Temperature	157.8 154.0 154.9 84.0 36.1	81.1 78.4 80.8 62.9 61.9

Categorical variables are reported as count (percentage of admissions). Continuous variables are reported as mean  ± standard deviation. MIMIC-IV records race while UCLH records ethnicity, reflecting the coding systems used in each source database; “Other White Bg” denotes Other White Background. Antibiotic frequencies may sum to more than 100% as patients can receive multiple antibiotics during an admission. The bottom section shows the mean number of vital sign measurements recorded per admission for each variable. Abbreviations: Pip.-Taz. (Piperacillin-Tazobactam), Sulfameth/Trimeth (Sulfamethoxazole-Trimethoprim), IV (Intravenous), SpO_2_ (Oxygen Saturation), BP (Blood Pressure).

The core task of our CDSS is to support daily IVOS reviews by predicting patient physiological stability. Unless otherwise stated, we forecast five key vital signs—heart rate, respiratory rate, oxygen saturation, systolic blood pressure, and temperature—over a 12-hour future window. A patient is defined as “switch-ready” if all forecast vital signs remain within clinically normal ranges throughout this window. We model these trajectories using Neural Processes (NPs)^17^, a class of probabilistic models chosen for their ability to handle the irregular sampling and uncertainty inherent in clinical time-series data. Detailed model architecture and training procedures are provided in Section 4.

### Forecasting

We first aim to establish the forecasting performance of the proposed neural process model. Table 2 shows the point forecasting performance of the proposed NP model and two baselines. The model is tasked with predicting the values of any measurements occurring in the 12-hour period immediately following the prediction time, defined as the point up to which patient history is known. The repeat baseline simply uses the last measured value prior to the forecast window as the prediction. The gradient boosted decision tree (GBDT) is more sophisticated and uses a set of statistical features generated from past measurements^18^. For discussion of baseline construction, see Section 4. The NP model provides a significant improvement over both baseline models on all measurements except SpO_2_, although the GBDT scores are only marginally worse. The mean improvement in the NP scores relative to the GBDT across variables was 2.25% for UCLH and 2.99% for MIMIC. The UCLH data is more easily forecastable than the MIMIC data, as reflected in the lower errors for all models in each measurement. This is likely due to the fact that the MIMIC ICU cohort is made up of patients with more serious, less stable conditions. In addition, ICU patients frequently present with severe non-infectious comorbidities which can perturb vital signs in ways that are not driven by the response to antibiotic therapy, further increasing the difficulty of forecasting.

**Table 2:** Mean absolute error of model forecasts for 12-hour prediction horizon

Dataset	Model	Variable				
		HR	RR	SpO_2_	SBP	Temp.
		(bpm)	(bpm)	(%)	(mmHg)	(^∘^F)
**UCLH**	**Repeat**	8.79 [8.76–8.82]	1.97 [1.96–1.98]	1.51 [1.51–1.52]	12.86 [12.81–12.91]	0.69 [0.69–0.70]
	**GBDT**	7.94 [7.92–7.96]	1.76 [1.76–1.77]	**1.31 [1.30–1.31]**	10.86 [10.83–10.89]	0.58 [0.58–0.58]
	**NP**	**7.58 [7.56–7.60]**	**1.71 [1.70–1.72]**	**1.30 [1.30–1.31]**	**10.71 [10.67–10.75]**	**0.57 [0.57–0.57]**
**MIMIC**	**Repeat**	9.17 [9.14–9.19]	4.09 [4.08–4.11]	2.05 [2.04–2.05]	14.29 [14.24–14.34]	0.63 [0.62–0.63]
	**GBDT**	8.92 [8.90–8.94]	3.49 [3.48–3.50]	1.79 [1.78–1.79]	12.27 [12.24–12.30]	0.55 [0.55–0.55]
	**NP**	**8.32 [8.29–8.34]**	**3.40 [3.39–3.41]**	**1.76 [1.76–1.77]**	**11.97 [11.92–12.01]**	**0.54 [0.54–0.54]**

The 95% confidence intervals are shown, computed by a bootstrap resampling of the mean error over each of the windows in the test set. Bold marks the best model and any not significantly worse (paired bootstrap, *p*≥0.05; Methods). Variable abbreviations: HR (Heart Rate), RR (Respiratory Rate), SpO_2_ (Oxygen Saturation), SBP (Systolic Blood Pressure), Temp. (Temperature). Model abbreviations: GBDT (Gradient Boosted Decision Trees), NP (Neural Process).

**Fig. 2:**
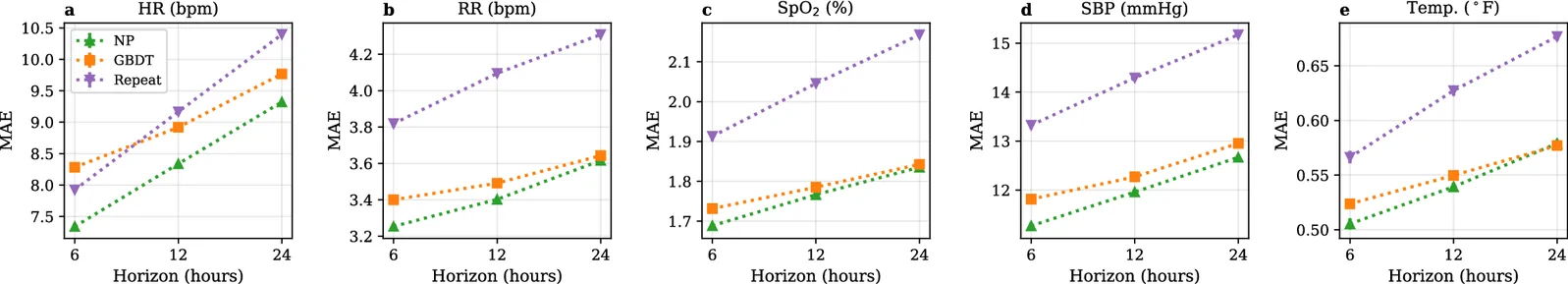
Vital-sign forecasting error across prediction horizons on MIMIC. Panels show the mean absolute error for **a** heart rate (HR), **b** respiratory rate (RR), **c** oxygen saturation (SpO_2_), **d** systolic blood pressure (SBP) and **e** temperature (Temp.). Note: the horizontal axis scale is logarithmic. Points show the median over bootstrap resamples of the test set and error bars show 95% bootstrap confidence intervals. Model abbreviations: GBDT (Gradient Boosted Decision Trees), NP (Neural Process).

Although a 12-hour forecast horizon is most appropriate for our clinical context, this is likely to vary across institutions with different workflows. As such, we also validate the model performance on two additional horizons: 6 hours and 1 day. Fig. 2 shows the result of this experiment for the MIMIC data. As expected, the mean absolute error increases as the forecast horizon increases; points further into the future are forecasted with lower accuracy. For the shorter horizons, the NP is significantly better than all baselines across variables. As the horizon increases, the performance of the GBDT and NP converge, with the models providing similar performance on the 1-day horizon. We note that the NP model is probabilistic and, as such, produces a forecasting distribution over possible measurement values as opposed to a single point estimate like the baseline models. This probabilistic output enables its predictions to be used downstream for the purposes of ranking, patients’ readiness for switching, as we will see in the following section. Table 2 does not assess this probabilistic aspect of the model and quantifies only the point forecasting performance. We additionally evaluate the NP under the continuous ranked probability score (CRPS), a strictly proper scoring rule that integrates over the full predictive distribution (Supplementary Note 2).

### Switch-readiness prediction

The key machine learning task in the proposed CDSS is the prediction of a patient’s readiness for switching in the forecast window. As noted in the Introduction, “switch-readiness” here refers to the physiological-stability component of the IVOS decision; the model does not predict oral suitability or other non-vital-sign factors, which remain with the reviewing clinician. We use a set of clinical criteria, which define a normal range for each of the key vital measurements, as a proxy for switch readiness. If a patient’s measurements fall within the normal range over the forecast window, we label them as ready to be switched on that day. The two datasets have disparate class distributions, reflecting the differences in the populations: in the UCLH data, patients are ready to be switched on 40.2% of the test days, compared to only 12.2% in the more unwell MIMIC ICU population.

The NP model produces a distribution over predicted measurements. We use this distribution to determine the probability a patient will meet the criteria in the forecast window, and thus the probability that they are ready to be switched. Patients with a higher probability of switch-readiness are ranked higher in the list presented to clinicians and thus are prioritised for review. The two-step process of first forecasting vitals, then applying criteria, gives an interpretable output that clinicians can use to inform their decision-making, since the prediction of the patient’s state underlying the predicted probability is clearly understandable.

An alternative, less interpretable, approach is to directly predict the switch readiness label using a supervised classification model. We apply the direct prediction method to serve as a baseline. As inputs to the baseline models, we use a set of statistical features generated from the past measurements. We apply both logistic regression and gradient-boosted decision trees. We additionally include STraTS^19^, a transformer-based classifier purpose-built for sparse, irregularly sampled multivariate clinical time series, as a representative deep-learning baseline that operates directly on the raw measurement sequence rather than on tabular features. For the NP model, we use two variants: the standard NP, as described above, and a variant tuned to produce well-calibrated probabilities (NP-Tuned). This variant uses an additional classification head that operates on the encodings generated by the final layer of the forecasting model to directly predict switch readiness. The issue of calibration is discussed further in Supplementary Note 1. Details for all baselines are given in Section 4.

In order to evaluate the ability of the models to predict the readiness of the patients for switching, we use four metrics: AUROC, average precision (AP), Brier score, and precision@5 (see Supplementary Methods for detailed definitions). These metrics are chosen to align with the intended deployment of the models: producing a ranked list of patients most likely to be ready for review. The AUROC gives a global measure of the model’s ability to discriminate between the positive and negative classes, representing the probability that a randomly chosen positive patient is ranked higher than a randomly chosen negative one. The average precision is also reported because, unlike AUROC, it is particularly sensitive to the model’s performance on the positive (switch-ready) class, of which there is a lower proportion. A high average precision score indicates that the model populates the top of the ranked list of predicted probabilities with a high concentration of true positives. The Brier score is included to assess the accuracy and calibration of the probability scores themselves; a low Brier score indicates the model’s predicted probabilities reliably represent the true chance of the event occurring^20^. The precision@5 metric corresponds to the proportion of the model’s top 5 ranked patients on each day that are truly ready to be switched, averaged over all days in the test set. This metric is intended to reflect the deployment context of the model, in which a ranked list will be presented to a clinician on a daily cadence. Sensitivity of this metric to the assumed review capacity is assessed by computing precision@*K* for a range of *K* values, alongside a per-day oracle, in Supplementary Note 2.

Table 3 shows the performance of each model on the test set from both the UCLH and MIMIC data; statistical significance was determined via bootstrap resampling (see Methods). These results indicate that both the GBDT baseline and the NP models are able to accurately rank patients on both datasets. A discussion of the advantages of the NP approach is provided in Section 3. For a random model, we would expect the AUROC to be 0.5, and the average precision and precision@5 to be equal to the proportion of positive examples (0.402 for UCLH and 0.122 for MIMIC). The precision@5, the metric most closely aligned with the intended clinical deployment, indicates that the NP model would select 2.2 × more relevant patients than random selection on the UCLH data, and 3.2 × on the MIMIC data. On the UCLH data, STraTS achieves the highest AUROC and average precision, the NP-tuned model achieves the best Brier score, and STraTS, NP-tuned and GBDT are statistically indistinguishable on precision@5. On the MIMIC data, STraTS attains the highest AUROC; STraTS, NP and GBDT are indistinguishable on average precision; NP and STraTS share the lead on precision@5; and the GBDT and NP-tuned achieve the best Brier scores. The strong performance of STraTS confirms that a modern irregular-multivariate time-series classifier can match or modestly exceed the NP on aggregate ranking metrics. However, unlike the NP, STraTS does not produce forecasts of individual vital signs and so cannot expose the per-variable predictions underlying the switch-readiness score to the clinician, an interpretability feature central to the proposed CDSS design (Section 3). The poor calibration of the standard NP model on both datasets is indicated by the elevated Brier score; this issue is discussed further in Supplementary Note 1. As in the case of forecasting, we find that the UCLH data is more predictable. The strong performance of all models in selecting relevant patients validates the overall approach of ranking patients for IVOS review by forecasting physiology and applying clinical criteria, rather than learning from historical switching decisions. Subgroup analyses stratifying performance by age, gender, ethnicity, and length of stay on both datasets are reported in Supplementary Note 4, and show that performance is broadly consistent across subgroups.

**Table 3:** Comparison of model performance for switch-readiness prediction

Dataset	Model	Metric			
		AUROC	AP	Brier Score	Precision@5
**UCLH**	**Logistic**	0.827 [0.823–0.831]	0.740 [0.732–0.747]	0.167 [0.165–0.169]	0.836 [0.823–0.850]
	**GBDT**	0.862 [0.858–0.865]	0.794 [0.787–0.800]	0.149 [0.147–0.151]	**0.899 [0.888–0.910]**
	**STraTS**	**0.870 [0.867–0.873]**	**0.805 [0.798–0.811]**	0.157 [0.155–0.159]	**0.904 [0.895–0.915]**
	**NP**	0.858 [0.854–0.861]	0.785 [0.779–0.792]	0.216 [0.212–0.219]	0.883 [0.871–0.894]
	**NP–tuned**	0.867 [0.864–0.871]	0.800 [0.793–0.807]	**0.146 [0.144–0.148]**	**0.901 [0.890–0.912]**
**MIMIC**	**Logistic**	0.759 [0.749–0.767]	0.300 [0.284–0.315]	0.098 [0.095–0.100]	0.280 [0.263–0.297]
	**GBDT**	0.869 [0.863–0.875]	**0.471 [0.453–0.490]**	**0.081 [0.079–0.084]**	0.381 [0.363–0.400]
	**STraTS**	**0.886 [0.881–0.892]**	**0.492 [0.472–0.513]**	0.159 [0.156–0.162]	**0.394 [0.375–0.413]**
	**NP**	0.882 [0.877–0.888]	**0.480 [0.461–0.500]**	0.106 [0.102–0.110]	**0.397 [0.377–0.417]**
	**NP–tuned**	0.862 [0.856–0.868]	0.453 [0.433–0.472]	**0.083 [0.080–0.085]**	0.369 [0.350–0.387]

Higher values are better for all metrics except the Brier score. Bold marks the best model and any not significantly worse under a two-sided paired bootstrap difference test against it (1000 resamples, Bonferroni-corrected across the *k* − 1 comparisons, *α* = 0.05; Methods). Metric abbreviations: AUROC (Area under the Receiver Operating Characteristic), AP (Average Precision). Model abbreviations: GBDT (Gradient Boosted Decision Trees), NP (Neural Process).

**Fig. 3:**
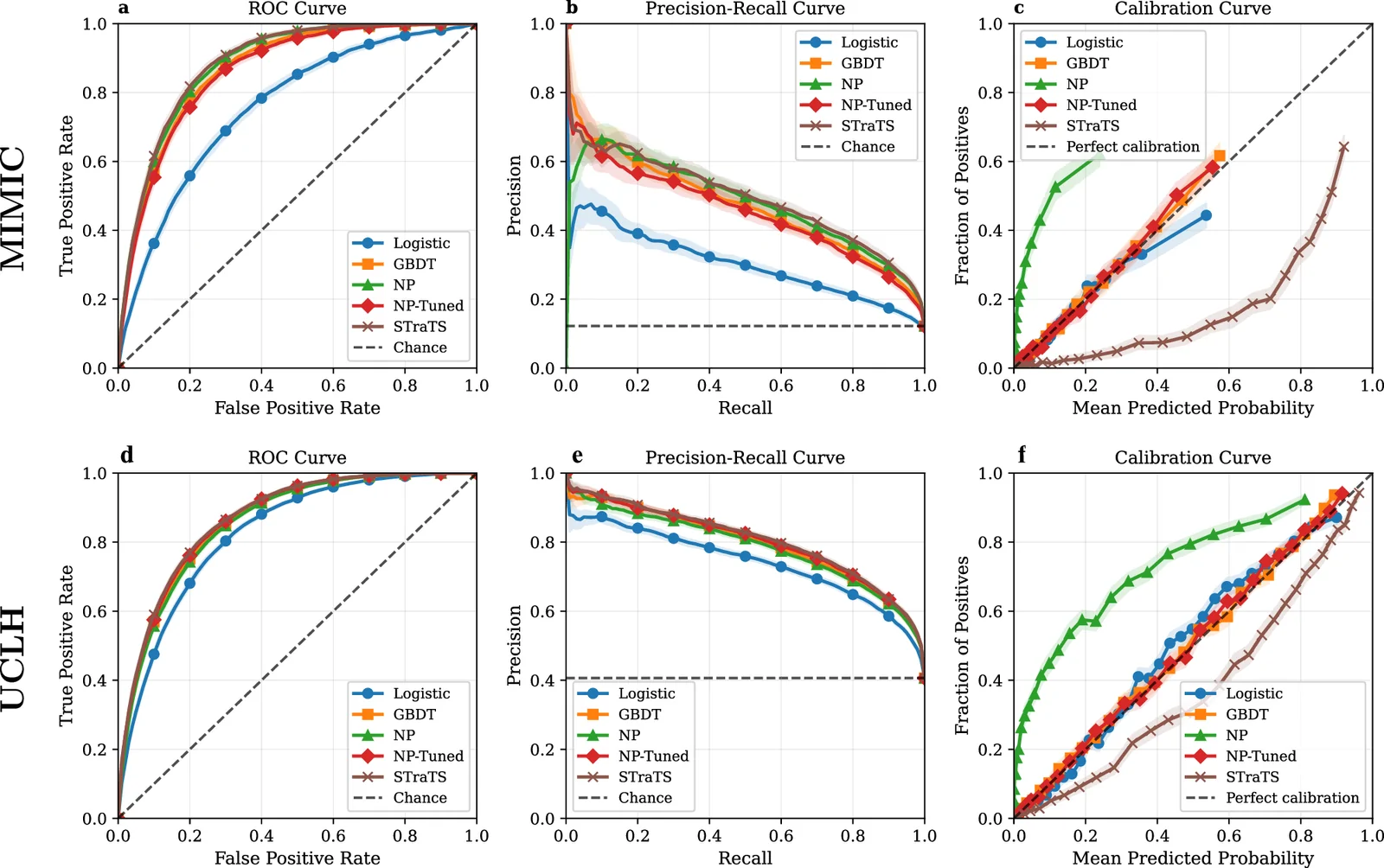
Discrimination, precision–recall and calibration of switch-readiness models on both datasets. Rows are labelled by dataset. **a** Receiver Operating Characteristic, **b** precision-recall, and **c** calibration curves on MIMIC; **d**–**f** show the same plots on the UCLH data. For the ROC and precision-recall curves the dashed line shows the performance of a chance model; for the calibration curves it shows a perfect model. For the calibration curves, aggregation bins are the quantiles of the distribution of predicted probabilities; the small proportion of positive examples in MIMIC means the curves do not span the full range of the plot. Curves show the median over bootstrap resamples of the test set and the shaded region shows 95% bootstrap confidence intervals. Model abbreviations: GBDT (Gradient Boosted Decision Trees), NP (Neural Process).

**Fig. 4:**
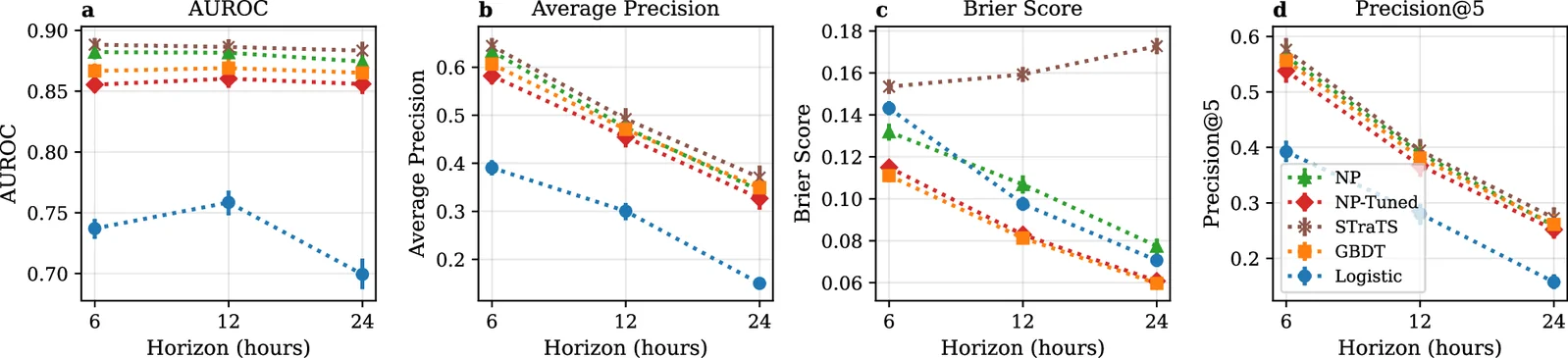
Switch-readiness ranking performance across prediction horizons on MIMIC. **a** AUROC, **b** average precision, **c** Brier score and **d** precision@5. Horizontal axis scale is logarithmic. Points show the median over bootstrap resamples of the test set and error bars show 95% bootstrap confidence intervals. Each metric is computed over the held-out MIMIC test tasks, where a task is one forecast window for one patient encounter: *n* = 22,560 tasks (4,536 positive, 20.1%) at 6 h, *n* = 22,615 (2761 positive, 12.2%) at 12 h and *n* = 22,680 (1782 positive, 7.9%) at 24 h. The counts differ slightly between horizons because the horizon changes how many target windows meet the inclusion criteria within each encounter. Note that the classes become more imbalanced as the forecast horizon increases. Model abbreviations: GBDT (Gradient Boosted Decision Trees), NP (Neural Process).

Figure 3 shows performance curves for each of the models on both datasets. These curves reinforce the finding that the GBDT, NP, NP-tuned and STraTS models perform similarly well in ranking patients on both datasets, with the NP and STraTS models providing a small improvement on MIMIC. The poor calibration of the standard NP is evident in the calibration curve, where the model consistently underestimates probabilities. All other models, including the tuned NP, are well calibrated on both datasets. We also validate the ranking performance of the NP models and baselines on varying forecast horizons, with results shown in Fig. 4. The ordering of the models remains consistent across forecasting horizons, with all of the NP and GBDT and STraTS providing strong performance. Note that the Brier score decreases as the forecast horizon increases, despite the problem being harder, because the fraction of positive instances decreases.

Our primary concern is the performance of models at the task of ranking patients as most ready for switching, as this is most closely aligned with the intended deployment of the models. Ranking does not require setting a binary decision threshold, and we do not intend to use the models to directly make a decision to switch or not. Nevertheless, it may still be instructive to compare the models’ ability to predict this binary class. Table 4 provides a number of metrics assessing the binary prediction performance of the models. A decision threshold was set for each model by computing the value that gave the highest F1 score on the validation set. On the UCLH data, STraTS achieves the best F1 score, while STraTS and GBDT are statistically indistinguishable on accuracy and precision, and the Repeat baseline retains the best recall. On the MIMIC data, STraTS and NP share the best F1 score, STraTS and the Repeat baseline share the best recall, and STraTS, NP and GBDT are indistinguishable on precision, with GBDT achieving the best accuracy. Consistent with the forecasting and ranking results, performance is lower on the MIMIC dataset, where the low prevalence of the positive class leads to significantly reduced precision across all models.

**Table 4:** Comparison of model performance for binary classification, with the positive class representing a patient that is ready to be switched

Dataset	Model	Metric			
		F1 Score	Accuracy	Precision	Recall
**UCLH**	**Repeat**	0.704 [0.699–0.709]	0.700 [0.696–0.704]	0.587 [0.581–0.594]	**0.879 [0.874–0.884]**
	**Logistic**	0.719 [0.714–0.724]	0.736 [0.732–0.740]	0.634 [0.627–0.640]	0.832 [0.827–0.838]
	**GBDT**	0.744 [0.740–0.749]	**0.765 [0.761–0.769]**	**0.667 [0.661–0.673]**	0.843 [0.837–0.848]
	**STraTS**	**0.752 [0.747–0.757]**	**0.769 [0.765–0.773]**	**0.668 [0.662–0.675]**	0.860 [0.854–0.865]
	**NP**	0.741 [0.736–0.746]	0.754 [0.751–0.759]	0.649 [0.643–0.655]	0.863 [0.857–0.868]
	**NP–tuned**	0.748 [0.744–0.754]	0.764 [0.760–0.768]	0.660 [0.654–0.666]	0.864 [0.859–0.870]
**MIMIC**	**Repeat**	0.432 [0.419–0.446]	0.778 [0.773–0.784]	0.315 [0.303–0.327]	**0.691 [0.673–0.708]**
	**Logistic**	0.372 [0.359–0.385]	0.763 [0.758–0.769]	0.275 [0.264–0.287]	0.575 [0.557–0.594]
	**GBDT**	0.497 [0.482–0.511]	**0.861 [0.856–0.865]**	**0.444 [0.428–0.460]**	0.564 [0.545–0.581]
	**STraTS**	**0.528 [0.514–0.542]**	0.847 [0.842–0.852]	**0.423 [0.409–0.438]**	**0.701 [0.684–0.718]**
	**NP**	**0.518 [0.505–0.531]**	0.847 [0.843–0.852]	**0.422 [0.407–0.436]**	0.672 [0.656–0.691]
	**NP–tuned**	0.487 [0.472–0.502]	0.849 [0.844–0.854]	0.416 [0.401–0.432]	0.587 [0.569–0.607]

The proportion of positive instances is 40.2 and 12.2% for UCLH and MIMIC respectively, indicating predicting the majority class could achieve an accuracy of 59.8% and 87.8% in each case. Decision thresholds were chosen to maximise the F1 score on the validation set. 95% confidence intervals are shown in brackets. Bold marks the best model and any not significantly worse under a two-sided paired bootstrap difference test against it (1000 resamples, Bonferroni-corrected across the *k* − 1 comparisons, *α* = 0.05; Methods). A higher value is better for all metrics. Model abbreviations: GBDT (Gradient Boosted Decision Trees), NP (Neural Process).

### Illustrative example

**Fig. 5:**
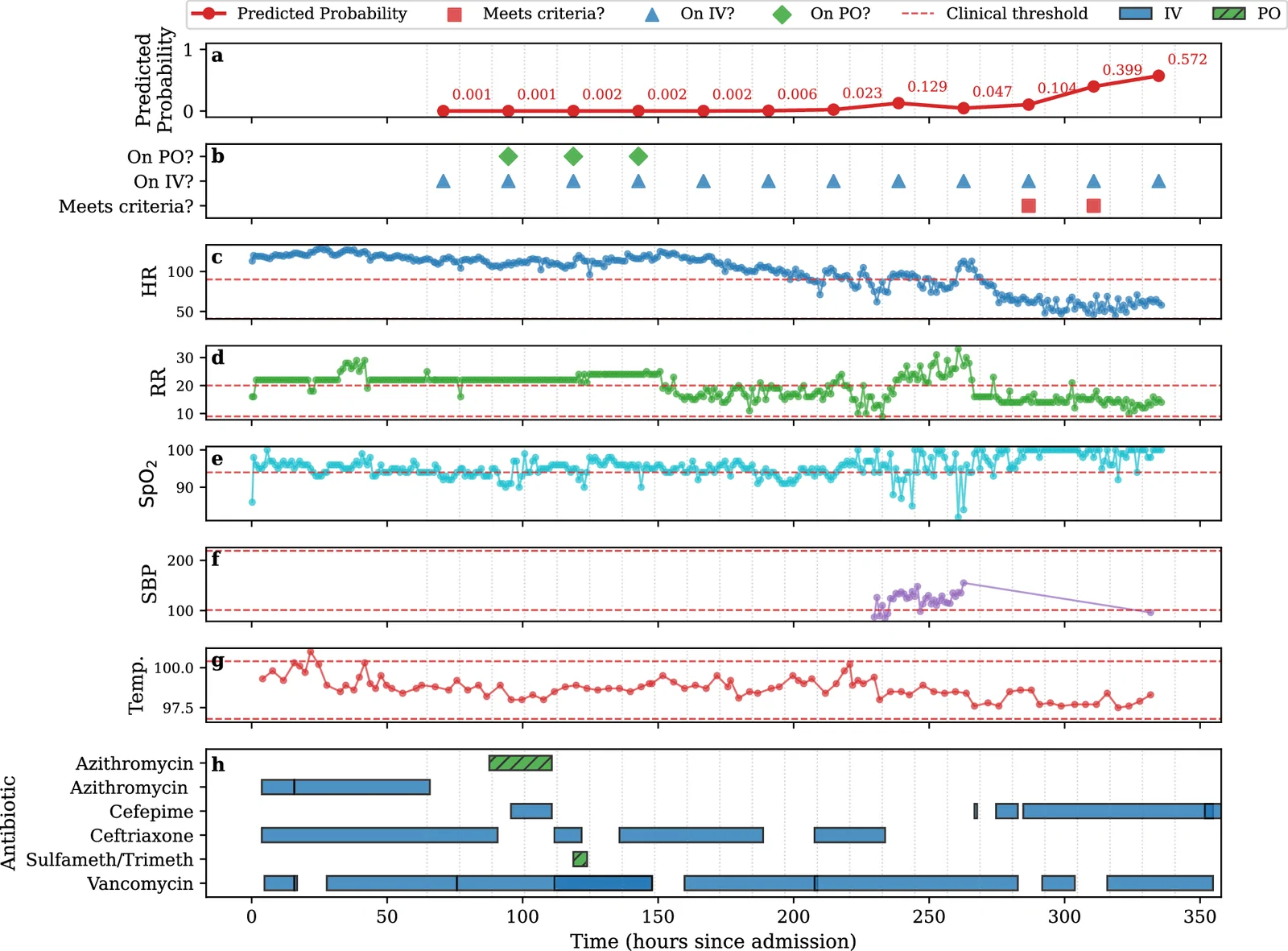
Illustrative MIMIC encounter showing the proposed system in operation. **a** predicted switch-readiness probabilities from the NP-tuned model; **b** labels indicating oral (PO) and IV antibiotic prescriptions and whether clinical criteria are satisfied in each forecast window; **c–g** measured vital signs with clinical thresholds for switch-readiness overlaid; and **h** antibiotic prescription timelines, with colour and pattern indicating administration route. Grey vertical lines demarcate daily 12-hour forecast windows. Forecasts begin 48 hours after admission to ensure sufficient historical data for prediction. Variable Abbreviations: HR (Heart Rate), RR (Respiratory Rate), SpO_2_ (Oxygen Saturation), SBP (Systolic Blood Pressure), Temp. (Temperature). Antibiotic Abbreviations: Sulfameth/Trimeth (Sulfamethoxazole-Trimethoprim).

To illustrate how the proposed system would function in clinical practice, we examine a patient encounter from the test set. Fig. 5 shows the progression of this encounter. Two further examples can be found in Supplementary Note 2. Over the course of approximately two weeks, the patient receives five different antibiotic agents across multiple separate prescriptions. As the encounter progresses, the patient’s vital signs steadily improve, particularly heart rate, temperature, and oxygen saturation. The model’s predicted switch-readiness probability tracks this clinical trajectory closely: remaining low during the initial period when the patient is more unwell, then rising substantially as physiological stability returns. Notably, despite the patient never being switched to oral therapy in reality, the model identifies the patient as ready before the end of their encounter, suggesting that an earlier switch could have been safely achieved had the proposed system been deployed.

## Discussion

While the example encounter in Fig. 5 example demonstrates successful model behaviour, it also reveals the inherent complexities of the IVOS problem that the system must address. Vital signs are sampled irregularly, with significant periods of missingness and frequent transient spikes that must be handled robustly. Antibiotic prescribing patterns rarely follow a simple sequence of an IV agent being replaced by its oral equivalent; in practice, multiple drugs may be prescribed concurrently for different indications, making switches difficult to identify from prescription data alone. A prescription of an oral drug does not necessarily indicate the patient’s state is improving. Labelling patients as meeting clinical criteria is similarly challenging: a single measurement marginally exceeding the threshold can cause the label to be negative, as occurs in the final forecast window where the patient’s heart rate briefly spikes. Note that it is also possible that two patients may have identical physiological measurements whilst one is suitable for switching and one is not; the underlying indication or issues with tolerance of the oral route might make the switch inappropriate.

Despite these complexities, the model produces clinically sensible predictions that track the patient’s physiological trajectory and could support timely identification of switch-ready patients. These challenges motivate the design choices discussed in Section 4, particularly the aggregation strategy for handling irregular measurements and the choice to forecast vital signs rather than directly predict switches.

A key contribution of this work is the formulation itself, which differs from previously proposed approaches for the integration of ML for IVOS decision support^14^. The NP, GBDT and STraTS models all produce substantially better results than random selection using our formulation, demonstrating that effective IVOS prioritisation can be achieved across a range of model architectures. These three models also perform similarly in terms of ranking performance, which raises the question: why use probabilistic forecasting at all? We stress that our intention is to create a practical machine learning system, as opposed to simply a performant model. Explainability is a critically important factor in the successful adoption of any ML-based CDSS^21^. Clinicians must feel confident in the suggestions of any model and have an understanding of how they have been generated. While the NP model itself is not interpretable, the system provides explainability through its outputs and architecture. The system produces forecasts of vital signs into the near future, quantities that clinicians already have a clear understanding of, and applies transparent clinical criteria to these forecasts to determine switch-readiness. When a patient is flagged as switch-ready, clinicians can inspect the underlying vital sign forecasts to understand why.

A further structural advantage of the NP approach is that a single model serves both the forecasting and classification tasks. Because the NP produces a full predictive distribution over future vital sign values, these distributions can be passed directly through the clinical criteria to yield switch-readiness probabilities, which in turn provide a principled ranking of patients for review. The baseline models, by contrast, produce only point estimates when used for forecasting and therefore cannot generate the distributional output needed to assess the probability that a patient’s vital signs will remain within normal ranges. As a result, two entirely separate GBDT models are required: one for forecasting and one for classification, each trained on different objectives and feature representations. Beyond the practical burden of maintaining two independent models, this separation means that the classification model’s predictions are not grounded in an explicit forecast of the patient’s physiological trajectory, weakening the interpretable link between predicted vital signs and the switch-readiness score that clinicians would inspect. Similarly, the STraTS model, as formulated by ref. ^19^, produces probability predictions for binary outcomes and does not forecast vital signs at arbitrary times, so its switch-readiness output is not accompanied by the underlying per-variable trajectories.

The separation between forecasting and application of criteria allows the model to be used much more flexibly in deployment. Take the example of a patient on mechanical ventilation, which controls respiratory rate, potentially causing a patient to seem ready for switching when they’re still unwell. In this case, the measured respiratory rate may fall within normal ranges not because the patient has improved, but because the ventilator is artificially regulating their breathing. When using the forecasting approach, the respiratory rate can be discounted from the switch-readiness calculation very easily, or the threshold can be adjusted on a case-by-case basis. To quantify the impact of such a deployment-time adjustment, we re-evaluated the standard NP on the MIMIC test set with respiratory rate removed from both the probability calculation and the ground-truth criteria. The underlying forecasting model is unchanged, so the per-variable forecasting error is unaffected. The prevalence of switch-ready days rises from 12.2 to 27.2%, and the model’s ranking ability is preserved: AUROC moves from 0.882 to 0.844, while average precision (0.477 to 0.637) and precision@5 (0.311–0.624) increase, primarily reflecting the higher positive-class base rate. Full results are reported in Supplementary Note 2. Likewise, if recommended clinical thresholds change with new evidence, the proposed system can be adapted without retraining or adjusting the base model. This is highly beneficial when considering model deployment, since regularly updating and retraining models can add significant complexity^22^.

To the best of our knowledge, there is only a single work that discusses the application of ML to the IVOS problem in order to assess the suitability of patients for switching^14^. Their approach is to train a model to predict, for a given day of a patient’s encounter, whether that patient received IV antibiotics. By training a model to predict historical clinical actions (i.e., the administration of IV antibiotics), the resulting system will necessarily learn to replicate the existing patterns and frequency of IVOS decisions present in the training data. Clinical practice is changing, and for many conditions there is growing evidence that long courses of IV antibiotics are unnecessary^2,6,23^. If the goal of a CDSS is to improve upon current practice–in this case by encouraging earlier switches and thus reducing the total number of IV therapy days–then a model trained to mimic historical, suboptimal behaviour is inherently limited. This limitation is particularly acute for the complex or non–standard cases where clinical decision support is most needed. Although performance of such a model on held-out retrospective data might appear encouraging, this approach inevitably results in a brittle CDSS, frozen in time and incapable of providing continuous support to clinicians participating in a resilient Learning Health System^24,25^. Our approach of modelling patient physiology keeps the prescribing clinician front and centre and results in a CDSS sufficiently flexible to adapt to clinical evidence and changing guidelines.

Fundamentally, obtaining ground truth for switch-readiness is challenging. The decision to switch is multifaceted, involving factors beyond vital signs such as infection type and severity, antibiotic availability and contraindications, patient comorbidities, and social circumstances affecting discharge planning. Historical switching decisions, as recorded in EHRs, reflect suboptimal practice that the system aims to improve rather than replicate, making them unsuitable as ground truth. Even setting aside the issue of learning from prior practice, constructing reliable ground-truth IVOS labels from EHRs is difficult, and label quality caps model performance and clinical utility^26^. discuss the complexities of reconstructing antibiotic courses from prescription data alone.

The approach to label construction taken by ref. ^14^ contains methodological issues that result in a dataset that is noisy and systematically biased. IV and oral therapy periods are defined by taking the earliest start time and the latest end time across all antibiotic prescriptions of a given administration route within a single hospital stay, which can cause switches to be labelled incorrectly and systematically exclude more complex, but common, clinical scenarios, biasing the final dataset towards unrealistically simple and linear treatment trajectories. Additionally all antibiotics are treated as interchangeable, with no check to ensure that the oral drug is a clinically appropriate replacement for the IV drug, or that the prescription indications are for the same condition; for example, treatment with broad-spectrum IV antibiotics can cause C. difficile infection^27^, and in serious cases the IV course is halted while an oral antibiotic, often vancomycin, is used to control the C. difficile–disregarding indications, this would be labelled as a switch when in fact one has not occurred.

Our system does not rely on the use of ground-truth switching labels and is therefore necessarily incomplete, capturing only the physiological improvement component of the decision. The appropriateness of this proxy can only be validated through prospective deployment, where clinicians review flagged patients and provide feedback on the relevance and timing of recommendations. We note that modelling of physiological factors only is by design; our system purposefully keeps the final switch decision in the hands of the clinician. Modelling clinician behaviour and hospital policy is fraught and leads to models that can reinforce sub-optimal practices. This issue is critically important when considering ML-based CDSSs.

The primary limitation of our work is the absence of prospective clinical evaluation. Without deploying the system in practice and measuring its impact on switching rates, the total number of IV therapy days, patient outcomes, and clinician decision-making, we cannot definitively assess whether the model’s predictions translate into improved clinical care. Our evaluation demonstrates that the model can accurately rank patients according to physiological criteria for switch-readiness, but retrospective performance metrics do not guarantee clinical utility. This limitation is shared across the IVOS CDSS literature: to our knowledge, no ML-based system for IVOS has been prospectively evaluated^14^, although^28^ conduct a trial which measures the influence of ML-based IVOS recommendations on clinician decision making for a small set of curated case vignettes. While rule-based systems have undergone prospective evaluation^10–12^, these studies typically combine the intervention with education and policy changes. We note that deploying any IVOS CDSS, including ours, would likely require accompanying education and policy support. Therefore, any prospective comparison would need to assess the added value of the CDSS alone. The system’s outputs may also shape clinician behaviour: a high false-positive rate could foster scepticism, while over-reliance could promote automation bias. Framing the output as a prioritised review list rather than a binary recommendation partly mitigates but does not eliminate these effects, which warrant explicit study in any prospective evaluation. Such an evaluation would also need to establish the value of the prioritisation itself: because assessing a single patient against the criteria is already quick, any benefit must instead come from directing finite review capacity across every eligible inpatient, which retrospective metrics cannot demonstrate.

The model’s reliance on five vital signs means that other potentially clinically relevant information is not incorporated. It is possible that better forecasting performance could be achieved by considering a broader range of input variables, for example, inflammatory markers (C-reactive protein, white blood cell count), which have been considered in prior work^29,30^. The simple set of five vitals was chosen because they are measured with sufficient regularity in the clinical contexts in which the proposed IVOS system could be applied. The forecasting model also assumes that the recent past is informative of the near future, which may not hold during rapid clinical deterioration or improvement, potentially leading to delayed detection of status changes. As discussed in Supplementary Note 1, the standard NP model exhibits calibration issues that arise from the interaction between the Gaussian likelihood and outliers in EHR data. While the tuned variant addresses this limitation, the need for this correction highlights the difficulty of modelling complex EHR data. We therefore recommend the NP-tuned variant for any application in which the predicted probability is to be interpreted directly rather than used only as a relative ranking score.

Finally, the limited availability of microbiology results in both datasets precluded subgroup analyses by proven infection, infection site, or antibiotic appropriateness. Because of this, it is possible that patients in the evaluation set did not have a bacterial infection at all, and it is therefore not established that forecasting performance is consistent across patients with proven infection and those without. We note, however, that most patients receiving IV antibiotics never have a microbiologically confirmed infection, while IVOS decisions must still be made for them. Because the system flags physiological stability and leaves the resulting action to the clinician, surfacing such patients still prompts a review that may identify either a switch or, where appropriate, a reassessment of the need for antimicrobials.

## Methods

This section details our data sources and preprocessing pipeline, the methodology for constructing switch-readiness labels, the neural process architecture used for forecasting, and the baseline models against which we compare.

This study is a retrospective analysis of de-identified, routinely collected data, with no prospective recruitment and no intervention. Use of the UCLH data was approved by the UCLH Data Access Process for Research under the “University College London Hospitals NHS Foundation Trust Data Only Studies” approval (REC reference 21/SW/0089; IRAS project ID 299136); individual informed consent was not required, as the study used de-identified data collected in the course of routine clinical care. The MIMIC-IV data are publicly available in de-identified form and were collected under a waiver of informed consent granted by the Institutional Review Board of the Beth Israel Deaconess Medical Centre.

### Data

To train and validate our models, we use two databases of routinely collected EHRs. The first is MIMIC-IV v3.1^16^, a publicly available, de-identified database of admissions to the intensive care unit (ICU) at the Beth Israel Deaconess Medical Centre in Boston, USA, spanning the years 2008-2022. The second is formed from an internal database of admissions to University College London Hospitals (UCLH) National Health Service (NHS) Foundation Trust, a group of 11 hospitals in London, UK, spanning the years 2019-2025.

The two datasets represent significantly different populations: comprising only ICU admissions, the MIMIC patients are generally much more unwell; the measurements of patient vital signs are taken much more regularly in the MIMIC data, and the properties of antibiotic prescriptions differ both in terms of route and agents. The datasets are collected from different countries, where care practices, particularly related to antibiotic prescribing, vary; characteristics of each dataset are shown in Table 1. For example, there is a different split of sex (40.7% female in MIMIC vs 52.6% in UCLH), and markedly different prescribing patterns: vancomycin is the most common IV antibiotic in MIMIC (65.7% of admissions) but is rarely used in UCLH, where co-amoxiclav is dominant (46.5%). Furthermore, the intensity of monitoring differs: while heart rate, respiratory rate, and oxygen saturation are measured approximately twice as frequently in MIMIC compared to UCLH, the difference is less pronounced for systolic blood pressure, and temperature is actually recorded less frequently in MIMIC.

From each database, we extract three categories of information: vital signs, stay information, and antibiotic prescriptions. Vital signs consist of five key physiological measurements: heart rate, respiratory rate, oxygen saturation (SpO_2_), systolic blood pressure, and temperature. These are recorded at irregular intervals throughout each patient’s stay. Stay information includes admission and discharge times. Antibiotic prescriptions comprise records of antimicrobial orders, including the drug name, route of administration (IV or oral), and the start and end times of each order.

We now formalise the data structure and task definitions. Let ${{{\mathcal{X}}}}=\{{X}_{1},{X}_{2},\ldots,{X}_{M}\}$ be a dataset of *M* independent hospital encounters. For each encounter *X*_*m*_, we have a set of *N*_*m*_ measurements taken from the patient over the course of their stay. Each measurement is one of *C* variables, in our case, the five vital signs described above. We use the triplet representation, so that each encounter can be written as the set ${X}_{m}={\{({t}_{m,i},{c}_{m,i},{x}_{m,i})\}}_{i=1}^{{N}_{m}}$, where ${t}_{m,i}\in {{\mathbb{R}}}^{+}$ is the time that the measurement of variable *c*_*m*,*i*_ ∈ {1, …, *C*} was taken, and ${x}_{m,i}\in {\mathbb{R}}$ is the measured value. Let ${X}_{m}^{\tau }=\{(t{\prime},c{\prime},x{\prime} )\in {X}_{m}| t{\prime} < \tau \}$ denote the history of the encounter prior to time *τ*. The forecasting task is to predict the patient’s state at some future time *t* > *τ* given ${X}_{m}^{\tau }$. The classification task is to predict a binary label ${y}_{m}^{\tau }\in \{0,1\}$ indicating whether the patient meets the clinical criteria for switch-readiness in a forecast window [*τ*, *τ* + *Δ*], given the history ${X}_{m}^{\tau }$. The methodology for determining these labels is discussed in Section 4.

We apply the following preprocessing steps. We drop any measurements outside a predefined range of physically plausible values, defined in Supplementary Table 1. We truncate encounters to a maximum length of 2 weeks. Paediatrics is already excluded from the MIMIC dataset. For the UCLH data, patients aged under 18 are excluded. As a condition of pseudonymisation required to extract the UCLH data, patients aged over 90 were removed. Encounters without any IV antibiotic prescription are excluded from the cohort. Throughout, we use the relative time since admission to index encounters. Unless otherwise stated, a history of 48 h is used for model inputs. To match the intended deployment of the model, we construct one task per day for each encounter, with the prediction time set to 9 am. A task is created on a given day only if the patient is considered to be on an IV prescription at the prediction time, there are at least 10 measurements in the preceding 48-hour look-back window, and there is at least one measurement in the 12-hour prediction window (9 am to 9 pm). A patient is considered to be on IV medication if they have an active IV antibiotic order at the prediction time, or if an IV order ended within the preceding 36 hours. More sophisticated approaches for reconstructing antibiotic courses do exist^26^, but were not employed in this study.

To simulate a realistic deployment scenario and prevent look-ahead bias, we split the data temporally to form the test set. For the UCLH data, encounters admitted after 1 January 2024 form the test set. For the MIMIC data, we use a cutoff of 1 January 2019; however, the de-identification process in MIMIC-IV shifts all dates by a random patient-specific offset, which scrambles the chronological order of admissions between patients. We therefore reconstruct approximate admission dates to enable a temporal split, as described in Supplementary Methods. Because reconstructed admission dates are approximate, the precision@5 metric should be interpreted with more caution for the MIMIC data relative to the UCLH data. A validation set is created by selecting 10% of non-test data, with the remainder forming the training set. A full breakdown of the number of tasks in each of the training, validation and test sets for each of the datasets is given in Supplementary Table 2. Because the temporal split is encounter-based, a small minority of patients can appear in both the training and test splits via separate admissions. On MIMIC, this overlap is 10.9% of test encounters. Recomputing the test-set metrics on the disjoint subset of test encounters whose patients do not appear in training leaves all reported metrics unchanged within their bootstrap confidence intervals (Supplementary Note 3).

The baseline classification models are trained and evaluated using tasks constructed as described above. However, for training and evaluating the forecasting models, we construct tasks differently to better represent the general forecasting ability of each model rather than the specific switch-readiness prediction setting. For each encounter, we create tasks by uniformly sampling prediction start times *τ*. For each *τ*, we use a 48-hour look-back window as context to predict all measurements within a subsequent 12-hour forecast horizon after *τ*. We sample tasks with a frequency proportional to encounter length, generating one task for every 24 hours of data, so a 7-day encounter would produce 7 tasks with randomly spaced prediction times. This follows standard practice for task construction when training NPs^31^.

### Labelling switches

We define switch-readiness labels based on whether a patient’s vital signs fall within clinically normal ranges over the forecast window [*τ*, *τ* + Δ]. In line with the scope set out in the Introduction, these labels capture only the physiological-stability component of the IVOS decision; non-vital-sign factors (oral suitability, infection type and severity, contraindications, social circumstances) are not encoded in the label and are intended to be assessed by the reviewing clinician. For each vital sign *c*, we specify lower and upper thresholds representing the normal range; these thresholds are given in Supplementary Table 3. A number of different definitions of clinically normal ranges exist in the context of IVOS^8^. We consulted with the microbiology team at our institution to determine the levels at which they would consider a patient suitable for review, anchoring the threshold values to the NEWS-2 score-1 boundaries (the levels at which a NEWS-2 score of 1 begins to be assigned), and maintained these levels across both the MIMIC and UCLH datasets. It may be that different levels are appropriate in different contexts; it is simple to adjust these in our framework, and the result would be an altered proportion of patients meeting criteria. In order to investigate the sensitivity of our results to a change in the clinical thresholds, we re-ran the MIMIC ranking experiments with less stringent thresholds, resulting in an increase in the number of positive (switch-ready) days from 12.2% to 50.0%. We found that the top-performing model(s) for each of the ranking metrics (AUROC, AP, Brier score, and precision@5) were nearly identical. For full results, see Supplementary Note 2.

A key consideration in constructing these labels is that the times at which measurements are taken are not known in advance. During model deployment, predictions must be made at fixed evaluation times without knowledge of when future measurements will occur. Evaluating criteria at the actual measurement times would introduce look-ahead bias, since these times are only available retrospectively. To address this, we aggregate measurements within the forecast window onto a fixed grid. We divide the 12-hour forecast window into four 3-hour intervals and compute the median value of each vital sign within each interval. A patient is labelled as switch-ready (${y}_{m}^{\tau }=1$) if all aggregated values fall within their respective normal ranges. If no measurements are available for a given vital sign in an interval, that interval is excluded from the criteria check for that variable. This choice to treat missing values as meeting criteria, rather than violating, is a modelling decision that must be clearly communicated to end users; the alternative would result in substantially more imbalanced class distributions, since missing data is common.

### Neural processes

Neural Processes (NPs) are a type of probabilistic meta-learning model that uses neural networks to parametrise the prediction map from the space of possible datasets to the space of stochastic processes representing the model’s probabilistic predictions at an arbitrary set of target points^17,31^. While the preceding definition is technically accurate, it is perhaps of limited utility in understanding how NPs can be useful for the forecasting of vital signs. In the parlance of meta-learning, we can view each of the encounters described in Section 4 as an individual task or dataset. The set of encounters is then known as the meta-dataset. We aim to build a model that, when provided with data from an unseen encounter, can make predictions about the values of the patient state. NPs generally solve this problem by taking the dataset of past tasks and splitting each task into a context set and a target set. The model is then trained to produce well-calibrated probabilistic predictions at a set of target inputs using the information in the context set. In this case, the context set is all the measurements of vital signs in the look-back window, and the target points are the measurements in the forecast window.

Convolutional conditional neural processes (ConvCNPs) are a type of NP which encode the property of translation equivariance into the prediction map^32^. Translation equivariance implies that if the time values of the data are shifted by a constant time, the model’s predictions will also be shifted by that time. Translationally equivariant NPs map to stationary stochastic processes, meaning they are a useful model class for temporal or spatio-temporal problems where stationarity is a reasonable inductive bias^31^. ConvCNPs use a functional encoding based on a kernel smoothing of the input data, followed by processing with a convolutional neural network. A description of the model architecture, training objective, and the derivation of switch-readiness probabilities from the predictive distributions is provided in Supplementary Methods.

Our ConvCNP uses a UNet architecture^33^, with 4 convolutional layers before and after the bottleneck, and a length scale of 1 hour for the kernel smoothing. At each target time *t* and for each vital sign *c* in the forecast window, the model outputs the parameters of a Gaussian predictive distribution; these predictions are conditionally independent given the context data ${X}_{m}^{\tau }$. The model is trained by minimising the negative log-likelihood of the target observations under this predictive distribution. We train for up to 500 epochs with early stopping (patience of 250 epochs), using a batch size of 512. We perform a grid search over learning rate and number of channels, selecting the model with the lowest validation loss. Full hyperparameter settings are given in Supplementary Table 4. All models were trained on a single NVIDIA A10 or NVIDIA A40 GPU. In wall-clock terms, training a single NP on the MIMIC dataset took 25 minutes, with a further 20 minutes for the NP-tuned classification head on top, a STraTS model 1 hour, and a single GBDT 1 minute on this hardware.

To compute switch-readiness probabilities, we consider two approaches. In the standard NP model, for each vital sign, we produce predictive distributions at forecast times aligned with the centre of the 3-hour aggregation intervals described in Section 4. We then compute the probability that the measurement at each point falls within the clinical threshold range using the cumulative distribution function of the Gaussian. The overall switch-readiness probability is then the product of these individual probabilities across all vital signs and time points, under the conditional independence assumption. We empirically verify that this assumption does not materially affect performance: on the MIMIC test set, probabilities computed this way agree closely (Spearman 0.86) with those from autoregressive samples^34^, an established technique for drawing coherent correlated samples from NPs trained on marginals, and downstream ranking metrics are at least as good (Supplementary Note 2). In the tuned variant (NP-tuned), we add a classification head to the forecasting model. This head aggregates the encoding from the final convolutional layer across the time dimension using a max-pooling operation, then applies a multi-layer perceptron to output a single switch-readiness probability. The classification head is trained with the forecasting model weights frozen. Architecture details for the classification head are given in Supplementary Table 5.

### Baselines

We develop baseline models for both the forecasting and classification tasks. These baselines use tabular models that take as input a fixed-length feature vector derived from the look-back window ${X}_{m}^{\tau }$. For each vital sign *c* ∈ {1, …, *C*}, we extract 16 features from the measurements in ${X}_{m}^{\tau }$: statistical summaries (mean, standard deviation, minimum, maximum, median, count, 25th and 75th percentiles), temporal features (time since the most recent measurement, time span of observations, and measurement frequency), and trend features (mean and standard deviation of successive slopes, total change from first to last observation, and counts of positive and negative changes). If a measurement type is entirely absent for a given task, its corresponding features are imputed with default values. We also compute two global features describing missingness: the count of absent measurement types and an overall completeness ratio. In total, there are 82 features for the classification task.

The classification baselines directly predict the switch-readiness label ${y}_{m}^{\tau }$ from the feature vector. We apply both logistic regression, using the scikit-learn implementation^35^, and gradient boosted decision trees (GBDT), using the LightGBM implementation^18^. We perform a grid search over hyperparameters, selecting the model with the highest average precision on a held-out validation set; the hyperparameter search space is given in Supplementary Table 6. These models serve as a more challenging baseline for the NP-based system, since they directly optimise the classification objective rather than first forecasting vital signs and then applying criteria.

In addition to the tabular baselines, we include STraTS^19^, a transformer-based classifier designed for sparse and irregularly sampled multivariate clinical time series. STraTS represents each measurement as a (time, variable, value) triplet and uses self-attention over the entire look-back window to produce a single switch-readiness probability. We train STraTS on the same daily tasks used for the other classification baselines, omit static demographic features for fair comparison, and adopt the hyperparameter settings recommended in the original paper with a learning-rate sweep matched to that used for the NP; the resulting model has approximately 48k parameters. Note that STraTS produces only a binary switch-readiness probability and cannot be queried for individual vital signs at arbitrary future times, so it is not included in the forecasting comparison.

For the forecasting task, we use two baselines. The repeat baseline simply uses the most recent measured value of each vital sign prior to the forecast window as the prediction for all future time points. The GBDT forecasting baseline is more sophisticated and must account for the irregularity of the target times and the multiple output variables. Each time point in the forecast window is treated as a separate prediction instance. The shared feature vector representing the look-back window is augmented with a one-hot encoding specifying which vital sign *c* is being predicted and the time elapsed since the prediction time *τ* (i.e., the forecast horizon). This formulation allows a single GBDT model to predict all vital signs at arbitrary future times within the forecast window. Neither baseline produces probabilistic forecasts, limiting their ability to quantify uncertainty in predictions.

### Statistics and reproducibility

Model performance was compared using paired bootstrap resampling. For each of 1000 bootstrap iterations, a single set of indices was sampled with replacement from the test set and used to select the predictions of every model, so that all metrics are computed on identical subsets of the data. Pairwise comparisons against the best-performing model were conducted using two-sided bootstrap difference tests, with a model considered significantly worse if the best model outperformed it in a proportion of iterations exceeding 1 − *α*/2. We applied a Bonferroni correction for multiple testing: for *k* models, the threshold was adjusted to *α*/(*k* − 1), with *α* = 0.05. Models not significantly different from the top performer are shown in bold in the results tables.

No statistical method was used to predetermine sample size; both cohorts comprise every encounter in the source database meeting the pre-established inclusion criteria described in Section 4, which also details the exclusions applied. The experiments were not randomised: allocation to the training, validation and test sets is by a temporal split on admission date, chosen to prevent look-ahead bias and to simulate prospective deployment. The investigators were not blinded to allocation during experiments and outcome assessment; blinding is not relevant to this retrospective computational study, as the switch-readiness labels are derived automatically by applying fixed clinical criteria to recorded vital signs, and all metrics are computed programmatically. Sex is reported for both cohorts in Table 1, and performance disaggregated by age, sex, ethnicity and length of stay is reported in Supplementary Note 4.

### Software

All analyses were performed in Python 3.12. The neural process models were implemented with the neuralprocesses library (v0.2.7)^36^ on PyTorch (v2.8.0), the logistic regression baseline and all evaluation metrics used scikit-learn (v1.7.2)^35^, and the gradient boosted decision tree baselines used LightGBM (v4.6.0)^18^. Data handling and analysis used pandas (v2.3.2), NumPy (v2.3.3) and SciPy (v1.16.1); figures were produced with Matplotlib (v3.10.6) and seaborn (v0.13.2); experiments were tracked with MLflow (v3.5.1). The analysis depends on a larger set of packages than can reasonably be listed here; the complete dependency set, with exact pinned versions, is given in the lock file in the code repository^37^.

### Reporting summary

Further information on research design is available in the Nature Portfolio Reporting Summary linked to this article.

## Supplementary information

Supplementary information

Reporting Summary

Transparent Peer Review file

## Data Availability

The MIMIC-IV dataset (version 3.1) is publicly available from PhysioNet under the PhysioNet Credentialed Health Data License, at https://doi.org/10.13026/kpb9-mt58^38^; access requires credentialed registration and completion of the required training. The UCLH dataset contains personally identifiable data that has not been approved for external release. Researchers wishing to validate or replicate the results on the UCLH dataset would need to be approved for research collaborations with University College London Hospitals NHS Foundation Trust, and to secure appropriate permissions from the UCLH/UCL Joint Research Office. Researchers who meet these requirements can contact the corresponding author for further information about access to the datasets. No source data are provided with this paper; all reported results are derived from the two datasets described above using the code cited below.

## Code availability

The computer code used in this research is available at https://github.com/SAFEHR-data/ivos-model-public, and the version used to produce the results reported here (v1.0.0) is archived on Zenodo at https://doi.org/10.5281/zenodo.21337708^37^.
