# Supplementary material for: Optimising antibiotic switching via forecasting of patient physiology
Source: Nat Commun. 2026 Aug 25;17:10475. doi: 10.1038/s41467-026-76715-w (PMC13635169; doi:10.1038/s41467-026-76715-w)
Supplement: Supplementary file 1 [file 41467_2026_76715_MOESM1_ESM.pdf]

Supplementary Information: Optimising antibiotic switching via forecasting of patient physiology

Magnus Ross[1*], Nel Swanepoel[2], Akish Luintel[3], Emma McGuire[3],
Ingemar J. Cox[1,4], Steve Harris[2], Vasileios Lampos[1†]

[1]Department of Computer Science, Centre for AI, UCL, UK
[2]Institute of Health Informatics, UCL, UK
[3]Department of Medical Microbiology, Infection Division, University College Hospital London, UK
[4]Department of Computer Science, University of Copenhagen, Denmark

Supplementary Discussion

There are a number of works that use simple electronic reminders to alert clinicians to review IVOS eligibility after a fixed period of time. Beeler et al. [10] implemented a system that alerts clinicians after the patient has been on a course of IV for longer than 60 hours. In order to be eligible to raise an alert the patient must have at least 24 hours left on their prescription, have normal neutrophils and no fever and be able to swallow. They found that this system resulted in a 17% drop in IV therapy days, in a prospective evaluation. Berrevoets et al. [11] built a similar tool that triggers an alert 72 hours after initial prescription if the patient's inflammation markers are showing signs of improvement, and the patient can tolerate the oral route and does not have an infection type classed as severe. They combine this system with weekly education sessions for clinicians and found a 13% drop in IV antibiotic usage after 72 hours in a prospective evaluation. Kan et al. [12] implement a system that generates an alert for patients with an IV antibiotic order that has been active for at least 48 hours and has no fixed duration, with no fever and an order for an oral diet. They find that this system, paired with a revision of IVOS policy and staff education leads to a significant improvement in switching rate in the post-intervention period. Quintens et al. [13] discuss the implementation of a more complex rule based on a set of 13 criteria, with the goal of increasing clinician acceptance of switch alerts. This rule concerns the suitability of oral treatment, and does not directly address the patient's state of clinical improvement.

The CDSSs discussed above have demonstrated that significant improvements in IVOS rates can be generated by the implementation of simple clinical rules, combined with education of clinicians. The rules discussed in these works primarily address the question of oral treatment suitability, and do not aim to assess the clinical improvement of the patient in a sophisticated way, if at all. In the present work, we aim to use ML to better understand this aspect of the IVOS decision. Note it is not possible to benchmark these rule based methods against the proposed system because we do not have access to reliable ground truth switching labels.

Supplementary Methods

Data setup

Temporal split

A key step in our experimental design is a strict temporal split of the data, which is necessary to simulate a realistic deployment scenario and prevent look-ahead bias. The de-identification process in MIMIC-IV shifts all dates for a given patient by a random offset, which preserves intra-patient timelines but scrambles the chronological order of admissions between different patients. A random split on this scrambled data would incorrectly mix past and future

*Email: magnus.ross@ucl.ac.uk
†Email: v.lampos@ucl.ac.uk

Variable	Range	Units
Heart Rate	10 – 400	bpm
Systolic Blood Pressure	0 – 400	mmHg
Oxygen Saturation	0 – 100	%
Respiration Rate	0 – 120	bpm
Temperature	50 – 120	°F

Supplementary Table 1: Ranges of plausible values used for data cleaning.

data, leading to an over-optimistic evaluation. To address this, we reconstruct an approximate, chronologically-sortable admission date for each encounter. Following the methodology described by the MIMIC-IV creators, we combine the real admission year, available in the `admissions` table as `anchor_year`, with the month and day from the shifted `admittime`. While this does not recover the exact date, it allows us to correctly order all encounters by year.

Number of tasks

	MIMIC-IV		UCLH	
**Partition**	**Forecasting**	**Switch-readiness**	**Forecasting**	**Switch-readiness**
Train	167,923	79,148	344,133	76,586
Val.	18,804	8,902	38,864	8,885
Test	52,537	22,615	186,759	41,053

Supplementary Table 2: Number of tasks in each dataset partition for the forecasting and switch-readiness prediction tasks.

Switch criteria

Measurement	Switch Criterion
Temperature (°F)	96.8 – 100.4
Heart Rate (bpm)	41 – 90
Respiratory Rate (breaths/min)	9 – 20
Systolic BP (mmHg)	101 – 219
SpO2 (%)	> 94

Supplementary Table 3: Physiological criteria for switch readiness.

Further neural process details

In this section, we provide a formal description of the Neural Process (NP) model. We employ a Convolutional Conditional Neural Process (ConvCNP) [32, 31], which defines a conditional distribution over target measurements given a context set of observed measurements.

Model definition

Following the notation established in the main text, let a patient encounter be represented by a set of observations $X_m = \{(t_{m,i}, c_{m,i}, x_{m,i})\}_{i=1}^{N_m}$, where $t_{m,i} \in \mathbb{R}^+$ denotes the observation time, $c_{m,i} \in \{1, \ldots, C\}$ indexes the vital sign variable (e.g., heart rate, temperature), and $x_{m,i} \in \mathbb{R}$ is the measured value. Let X_m^τ denote the history of encounter m prior to time τ, and let $X_m^{[\tau,\tau+\Delta]}$ denote the target observations in the forecast window. The ConvCNP models the predictive distribution $p(X_m^{[\tau,\tau+\Delta]} \mid X_m^\tau)$ through three components:

Encoder. The context set X_m^τ is mapped onto a functional representation via radial basis function (RBF) kernel smoothing, placing observations onto a discretised temporal grid. This encoding, known as ConvDeepSet [31, 39], maintains separate channels for data density and signal values for each vital sign variable.

Processor. A UNet convolutional architecture [33] processes the gridded representation. Our implementation uses four convolutional blocks before and after the bottleneck layer, with the number of feature channels D as a hyperparameter. Let Φ denote the composition of encoder and processor. The resulting representation is

$$\mathbf{R} = \Phi(X_m^\tau) \in \mathbb{R}^{D\times G}, \tag{1}$$

where G is the number of grid points in the discretised time domain.

Decoder. A convolutional layer with kernel size 1 projects the D feature channels to $2C$ output channels, yielding the mean $\mu_{t,c}$ and variance $\sigma^2_{t,c}$ for each vital sign $c \in \{1, \ldots, C\}$. Parameters at any continuous target time t are recovered by interpolating the projected grid.

Training objective

The Conditional Neural Process framework assumes that target observations are conditionally independent given the context set. The joint predictive likelihood factorises as

$$p(X_m^{[\tau,\tau+\Delta]} \mid X_m^\tau) = \prod_{(t_j,c_j,x_j)\in X_m^{[\tau,\tau+\Delta]}} \mathcal{N}\big(x_j \mid \mu_{t_j,c_j},\, \sigma^2_{t_j,c_j}\big), \tag{2}$$

where μ_{t_j,c_j} and $\sigma^2_{t_j,c_j}$ are the decoder outputs for time t_j and variable c_j.

The model is trained by minimising the negative log-likelihood (NLL) of the target observations:

$$\mathcal{L}_{\text{forecast}} = -\frac{1}{|X_m^{[\tau,\tau+\Delta]}|} \sum_{(t_j,c_j,x_j)\in X_m^{[\tau,\tau+\Delta]}} \log \mathcal{N}\big(x_j \mid \mu_{t_j,c_j},\, \sigma^2_{t_j,c_j}\big). \tag{3}$$

This objective encourages accurate point forecasts via the mean while learning uncertainty estimates via the variance that capture the noise inherent in vital signs data.

Switch-readiness probability

Throughout this section, "switch-readiness" refers to the physiological-stability component of the IVOS decision as defined in the main text; the probability below captures only whether forecast vital signs satisfy the clinical criteria, not non-vital-sign factors such as oral suitability. Switch-readiness is determined by whether all vital signs remain within clinically defined normal ranges throughout the forecast window $[\tau, \tau + \Delta]$. For each vital sign c, let $[\ell_c, u_c]$ denote the

acceptable range. Let $\mathcal{T} = \{t_1, \ldots, t_K\}$ be the set of evaluation time points on the forecast grid (at 3-hour intervals), and let $\tilde{X}_{t,c}$ denote the random variable for the predicted measurement of vital sign c at time t, distributed as $\tilde{X}_{t,c} \sim \mathcal{N}(\mu_{t,c}, \sigma^2_{t,c})$.

The probability that a patient is switch-ready, denoted $P(y^\tau_m = 1 \mid X^\tau_m)$, is the probability that all forecast measurements satisfy the clinical criteria:

$$P(y^\tau_m = 1 \mid X^\tau_m) = P\left(\bigcap_{t \in \mathcal{T}} \bigcap_{c=1}^{C} \ell_c \leq \tilde{X}_{t,c} \leq u_c\right). \tag{4}$$

Under the conditional independence assumption, this joint probability factorises:

$$P(y^\tau_m = 1 \mid X^\tau_m) = \prod_{t \in \mathcal{T}} \prod_{c=1}^{C} P\big(\ell_c \leq \tilde{X}_{t,c} \leq u_c\big). \tag{5}$$

Each marginal probability is computed analytically using the cumulative distribution function of the Gaussian.

Classification head

For the NP-tuned variant, we augment the model with a classification head to directly predict the binary switch-readiness label $y^\tau_m \in \{0, 1\}$. This head operates on the feature representation $\mathbf{R} \in \mathbb{R}^{D \times G}$ produced by the processor. Global max-pooling over the temporal dimension yields a fixed-size embedding:

$$h_d = \max_{g \in \{1,\ldots,G\}} R_{d,g}, \quad \mathbf{h} = [h_1, \ldots, h_D]^\top \in \mathbb{R}^D. \tag{6}$$

A multi-layer perceptron maps this embedding to a predicted probability $\hat{p} = \mathrm{MLP}(\mathbf{h}) \in [0, 1]$.

The classification head is trained in a second stage with the forecasting model weights frozen, using binary cross-entropy loss:

$$\mathcal{L}_{\text{class}} = -\big[y^\tau_m \log \hat{p} + (1 - y^\tau_m) \log(1 - \hat{p})\big]. \tag{7}$$

This two-stage approach calibrates probabilities to the binary task labels while retaining the feature representations learned from the forecasting objective.

Neural Process Hyperparameters

Parameter	Value
Architecture	
Number of layers	4
Number of channels*	{64, 128, 256, 512}
Lengthscale	1.0 hour
Training	
Learning rate*	$\{10^{-5}, 5 \times 10^{-5}, 10^{-4}, 5 \times 10^{-4}, 10^{-3}\}$
Batch size	512
Epoch size	16384 tasks
Validation epoch size	4096 tasks
Maximum epochs	500
Early stopping patience	250 epochs
Learning rate scheduler	Cosine with 50 epochs linear warmup
Data	
Lookback window	48 hours
Forecast window	12 hours
Forecast grid size	3 hours
Minimum task measurements	10
Training tasks per day	1.0

Supplementary Table 4: Neural process model hyperparameters. Parameters marked with * were selected via grid search, with the search space shown in parentheses.

Parameter	Value
Architecture	
Hidden layer 1 size	256
Hidden layer 2 size	128
Dropout rate	{0.0, 0.1, 0.5}
Training	
Learning rate	$\{5 \times 10^{-5}, 10^{-4}, 5 \times 10^{-4}, 10^{-3}\}$
Other settings	Same as base model

Supplementary Table 5: NP-tuned classification head hyperparameters. The classification head is trained with the base forecasting model weights frozen.

Parameter	Value
GBDT	
Number of estimators	{5, 10, 50, 100, 200}
Maximum depth	{3, 4, 5, 6}
Learning rate	{0.001, 0.005, 0.05, 0.1, 0.5}
Logistic Regression	
Inverse regularization strength (C)	{0.1, 1.0, 10.0, 100.0, 1000.0}

Supplementary Table 6: Baseline model hyperparameters. A grid search was performed over the sets of hyperparameters. The model with the highest average precision on the validation set was selected for evaluation on the test set.

Evaluation Metrics

We denote the test set as $\{(\hat{p}_i, y_i)\}_{i=1}^N$, where $\hat{p}_i \in [0, 1]$ is the predicted switch-readiness probability and $y_i \in \{0, 1\}$ is the true label for instance i. For a decision threshold $\theta \in [0, 1]$, let $\mathrm{TP}(\theta)$, $\mathrm{FP}(\theta)$, $\mathrm{TN}(\theta)$, and $\mathrm{FN}(\theta)$ denote the number of true positives, false positives, true negatives, and false negatives, respectively.

Area Under the ROC Curve (AUROC) The AUROC assesses the model's ability to discriminate between switch-ready and non-switch-ready patients. It represents the probability that a randomly selected positive instance receives a higher predicted probability than a randomly selected negative instance. The AUROC is computed as the integral of the Receiver Operating Characteristic curve, which plots the true positive rate against the false positive rate at varying thresholds:

$$\mathrm{AUROC} = \int_0^1 \mathrm{TPR}\big(\mathrm{FPR}^{-1}(t)\big)\, dt, \tag{8}$$

where the true positive rate and false positive rate at threshold θ are defined as

$$\mathrm{TPR}(\theta) = \frac{\mathrm{TP}(\theta)}{\mathrm{TP}(\theta) + \mathrm{FN}(\theta)}, \qquad \mathrm{FPR}(\theta) = \frac{\mathrm{FP}(\theta)}{\mathrm{FP}(\theta) + \mathrm{TN}(\theta)}. \tag{9}$$

In practice, this integral is approximated using the trapezoidal rule over the empirical ROC curve.

Average Precision (AP) Average Precision summarises the precision-recall curve and is particularly informative for imbalanced classification tasks. Unlike AUROC, which treats positive and negative classes symmetrically, AP focuses on performance with respect to the positive class.

AP is computed as the area under the precision-recall curve, approximated as the weighted sum of precisions at each threshold:

$$\mathrm{AP} = \sum_{k=1}^{K} \big[R(\theta_k) - R(\theta_{k-1})\big] \cdot P(\theta_k), \tag{10}$$

where $\theta_1 < \theta_2 < \cdots < \theta_K$ are the unique thresholds, and precision and recall are defined as

$$P(\theta) = \frac{\mathrm{TP}(\theta)}{\mathrm{TP}(\theta) + \mathrm{FP}(\theta)}, \qquad R(\theta) = \frac{\mathrm{TP}(\theta)}{\mathrm{TP}(\theta) + \mathrm{FN}(\theta)}. \tag{11}$$

A high AP indicates that the model achieves both high recall and high precision.

Brier Score The Brier score evaluates the accuracy and calibration of probabilistic predictions. It is defined as the mean squared error between predicted probabilities and observed outcomes:

$$\text{Brier} = \frac{1}{N}\sum_{i=1}^{N}(\hat{p}_i - y_i)^2. \tag{12}$$

A lower Brier score indicates better calibrated and more accurate probability estimates. The score ranges from 0 (perfect predictions) to 1 (maximally incorrect). A model that always predicts the class prior p achieves a Brier score of $p(1-p)$.

Precision@5 Precision@5 measures clinical utility by evaluating the model's ability to prioritise patients for review. For each day d in the test set with at least 10 active encounters, patients are ranked by predicted switch-readiness probability in descending order. Let $\mathcal{D}$ denote the set of valid days, and let $\pi_d(j)$ denote the index of the patient with the j-th highest predicted probability on day d. Precision@5 is the proportion of truly switch-ready patients among the top 5, averaged over all valid days:

$$\text{Precision@5} = \frac{1}{|\mathcal{D}|}\sum_{d\in\mathcal{D}}\frac{1}{5}\sum_{j=1}^{5} y_{\pi_d(j)}. \tag{13}$$

Supplementary Note 1: Model Calibration

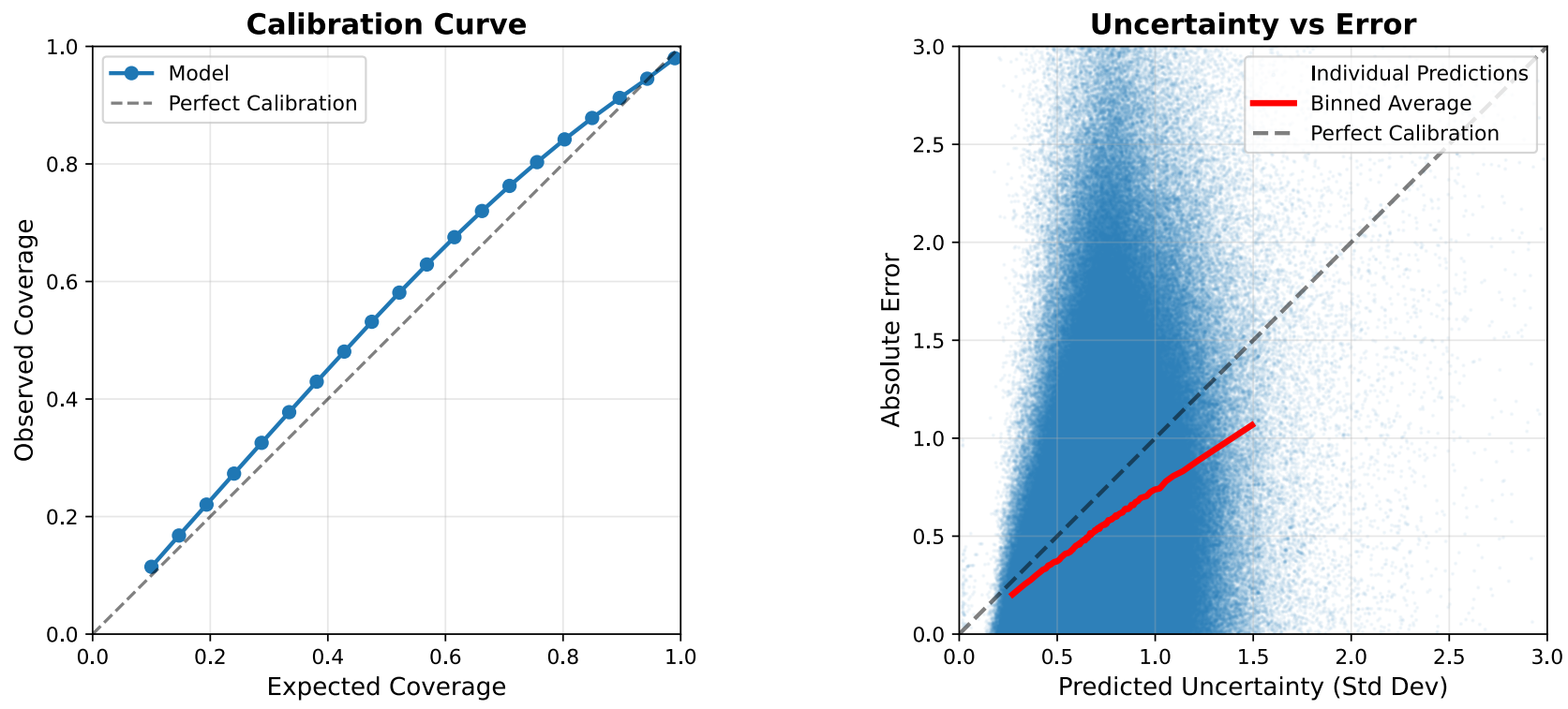

Supplementary Figure 1: Left plot shows calibration curve for the forecasting model's predicted uncertainty. For each confidence level (e.g., 80%), we generate a prediction interval that should contain the true value with that probability using the Normal predictive distribution. The x-axis shows the chosen confidence level while the y-axis shows the actual proportion of test observations that fell within the intervals. Perfect calibration follows the diagonal: an 80% interval should contain 80% of observations. Points above the diagonal indicate underconfident predictions. Right shows relationship between predicted uncertainty and absolute prediction error. Individual predictions are shown as scatter points, with binned averages over 1st to 99th percentiles (red line) summarising the trend. The diagonal represents ideal calibration where predicted uncertainty magnitude matches actual error magnitude. In this case, an underconfident model has binned average error below the diagonal; where the predicted uncertainty is higher than the actual error.

As shown in the main text results, the standard NP model exhibits poor calibration for the switch-readiness classification task, despite strong ranking performance. The elevated Brier score indicates that the model's predicted probabilities do not accurately reflect the true likelihood of switch-readiness. Specifically, the model consistently underestimates probabilities of switch-readiness.

This miscalibration stems from the interaction between the training objective and outliers in EHR data. The model minimises the negative log-likelihood of observed measurements under a Gaussian predictive distribution. Because Gaussian distributions have light tails, they assign very low probability to the measurement errors and transient spikes common in routinely collected vital signs. When the model encounters such outliers during training, the NLL becomes very large for predictions with narrow uncertainty, causing the model to learn to widen its predictive distributions. Supplementary Figure 1 demonstrates this through two complementary views: the left panel shows the calibration curve lies above the diagonal, indicating uncertainty estimates are too high, while the right panel shows binned average errors fall below the diagonal, meaning that the realised error of the model's forecasts is consistently lower than the predicted uncertainty.

When an underconfident forecasting model is used to predict switch-readiness by applying clinical criteria to the forecast distributions, this miscalibration propagates to the classification task. The wider predictive distributions increase the probability that forecast values will fall outside the normal ranges that define switch-readiness. This results in predicted switch-readiness probabilities that are systematically too low, producing the poor calibration observed in the results.

The NP-tuned variant addresses this issue by adding a classification head trained directly on the switch-readiness labels. This head learns to correct for the forecasting model's underconfidence, producing well-calibrated probabilities that match the observed frequencies. The tuned model achieves Brier scores comparable to the baseline methods while maintaining strong ranking performance. This approach allows the system to retain the interpretability benefits of the forecasting framework while providing calibrated probability outputs suitable for clinical decision support.

Supplementary Note 2: Additional results

Criteria threshold sensitivity

Measurement	Switch Criterion
Temperature (°F)	96.8 – 100.58
Heart Rate (bpm)	40 – 131
Respiratory Rate (breaths/min)	8 – 24
Systolic BP (mmHg)	90 – 229
SpO2 (%)	> 91

Supplementary Table 7: Alternative, loose criteria for switch readiness.

We tested the sensitivity of the proposed system to a change in clinical criteria since it is likely that different institutions will have different requirements. Supplementary Table 7 shows an alternative looser set of criteria. Applying these criteria to the MIMIC data resulted in an increase in the number of positive (switch-ready) days from 12.2% to 50.0%. The relative performance of our model was preserved when measured with the looser criteria. Results for the ranking metrics are shown in Supplementary Table 8 and for the binary metrics in Supplementary Table 9.

Discounting a vital sign at deployment

A key motivation for separating physiological forecasting from the application of clinical criteria is that individual vital signs can be discounted at deployment without retraining the forecasting

Dataset	Model	Metric			
		AUROC	AP	Brier Score	Precision@5
MIMIC-loose	Logistic	0.676 [0.669-0.682]	0.650 [0.640-0.660]	0.229 [0.228-0.231]	0.680 [0.663-0.696]
	GBDT	0.829 [0.824-0.834]	**0.809 [0.801-0.817]**	**0.168 [0.165-0.170]**	**0.835 [0.821-0.849]**
	NP	**0.836 [0.831-0.841]**	**0.810 [0.802-0.817]**	0.239 [0.235-0.243]	**0.841 [0.827-0.855]**
	NP-tuned	0.814 [0.809-0.820]	0.792 [0.783-0.800]	0.175 [0.172-0.177]	0.822 [0.808-0.838]

Supplementary Table 8: Model performance for switch-readiness prediction on the MIMIC dataset with looser clinical criteria (switch rate 50.0%). Bold marks the best model and any not significantly worse (paired bootstrap, $p \geq 0.05$; Methods). Model abbreviations: GBDT (Gradient Boosted Decision Trees), NP (Neural Process).

Dataset	Model	Metric			
		F1 Score	Accuracy	Precision	Recall
MIMIC-loose	Repeat	0.739 [0.733-0.745]	0.700 [0.695-0.706]	0.654 [0.647-0.662]	0.849 [0.842-0.856]
	Logistic	0.684 [0.678-0.690]	0.560 [0.554-0.567]	0.534 [0.527-0.541]	**0.950 [0.946-0.954]**
	GBDT	0.772 [0.766-0.777]	0.741 [0.736-0.747]	0.690 [0.683-0.697]	0.875 [0.869-0.881]
	NP	**0.779 [0.773-0.784]**	**0.753 [0.748-0.759]**	**0.707 [0.699-0.714]**	0.867 [0.861-0.872]
	NP-tuned	0.762 [0.756-0.768]	0.730 [0.724-0.736]	0.681 [0.674-0.689]	0.865 [0.859-0.872]

Supplementary Table 9: Model performance for binary switch-readiness prediction on the MIMIC dataset with looser clinical criteria (switch rate 50.0%). Bold marks the best model and any not significantly worse (paired bootstrap, $p \geq 0.05$; Methods). Model abbreviations: GBDT (Gradient Boosted Decision Trees), NP (Neural Process).

model. For example, the respiratory rate of a patient on mechanical ventilation is controlled by the ventilator and is therefore uninformative about clinical improvement. In this case a clinician would want to exclude respiratory rate from the switch-readiness calculation. We here quantify the effect of doing so on the MIMIC test set.

We re-evaluate the standard NP model with respiratory rate discounted from both the model's switch-readiness probability and the construction of the ground-truth label. The underlying forecasting model is not retrained: the same NP is queried at the same target times for the same vital signs, so per-variable forecasting error is unchanged. The intervention is entirely a deployment-time adjustment.

Results are reported in Supplementary Table 10. Excluding respiratory rate raises the prevalence of switch-ready days from 12.2% to 27.2%. Discrimination is broadly preserved: AUROC decreases modestly from 0.882 to 0.844, while average precision and precision@5 both increase substantially (0.477 to 0.637 and 0.311 to 0.624 respectively), reflecting the higher base rate of the positive class. The Brier score increases from 0.106 to 0.187; this is again driven principally by the change in class balance, since for the standard NP a higher proportion of positive labels exposes more of the systematic underconfidence of the Gaussian-likelihood

Setting	Positive rate	AUROC	AP	Brier Score	Precision@5
All vitals (baseline)	12.2%	0.882 [0.877–0.887]	0.477 [0.459–0.497]	0.106 [0.102–0.110]	0.311 [0.294–0.328]
Respiratory rate excluded	27.2%	0.844 [0.840–0.850]	0.637 [0.625–0.651]	0.187 [0.183–0.191]	0.624 [0.606–0.644]

Supplementary Table 10: Effect of discounting respiratory rate at deployment on switch-readiness metrics for the standard NP model, evaluated on the MIMIC test set. The baseline uses all five vital signs; the lower row drops respiratory rate from both the model's probability calculation and the ground-truth label. The forecasting model is unchanged across the two rows. 95% bootstrap confidence intervals are shown in brackets.

forecasting model (see Supplementary Note 1). The clinically relevant property, the ordering of patients by predicted switch-readiness, is preserved.

Method	AUROC	AP	Brier score
Product of marginals	**0.882 [0.877–0.888]**	**0.481 [0.461–0.500]**	**0.106 [0.102–0.110]**
Autoregressive joint	0.850 [0.843–0.858]	0.444 [0.425–0.463]	0.108 [0.105–0.112]

Supplementary Table 11: Comparison of the product-of-marginals and autoregressive joint methods for computing switch-readiness probability on the MIMIC test set, using the same trained NP. 95% bootstrap CIs in brackets; bold marks the best value on each metric under a paired bootstrap test ($p < 0.05$).

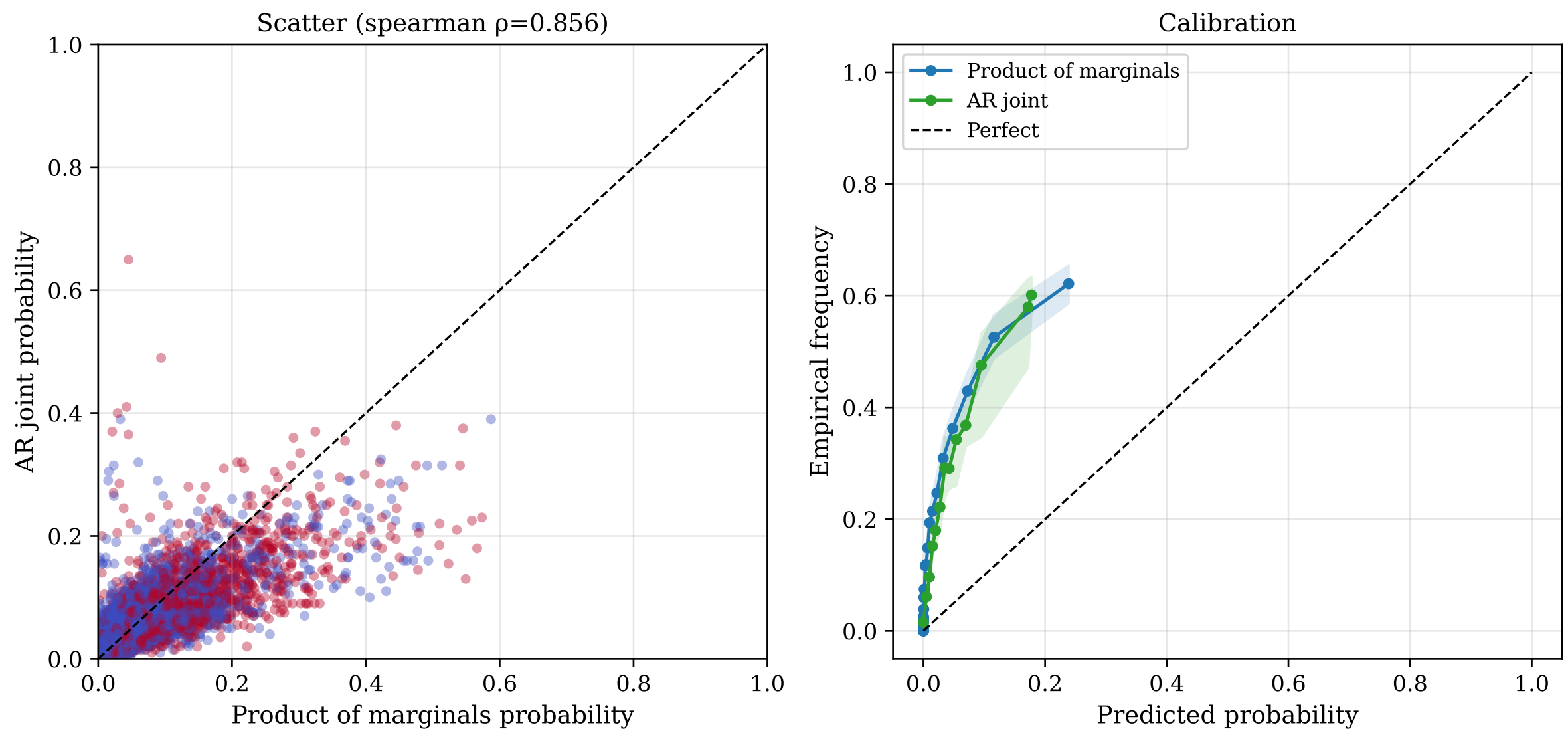

Supplementary Figure 2: Comparison of the product-of-marginals and autoregressive joint methods on the MIMIC test set, using the same trained NP. Left: per-encounter switch-readiness probability from the product of marginals (x-axis) against the autoregressive joint estimate (y-axis); the dashed line marks equality. Right: calibration curves for the two methods; curves show the median over bootstrap resamples of the test set and shaded regions denote 95% bootstrap confidence intervals.

Empirical assessment of the conditional independence assumption

In the standard NP described in Supplementary Methods, the overall switch-readiness probability is computed as a product of per-vital, per-interval Gaussian-CDF marginals, under a conditional independence assumption across vitals and forecast times. Physiological signals are typically correlated in practice, so this is a simplification of the true joint. Here we quantify its empirical impact on the MIMIC test set.

We compare two ways of computing the switch-readiness probability from the same trained NP. The first is the product of marginals used throughout the paper, the second is an autoregressive joint estimate, obtained by drawing $N = 200$ autoregressive samples from the same trained NP, in which each target point is sampled conditional on previously drawn targets [34], and then taking the fraction of samples in which all aggregated vitals fall within their thresholds. The autoregressive estimate explicitly captures correlations between vitals and forecast times. Both methods target the same event probability and differ only in whether cross-vital and cross-time correlations are modelled explicitly.

The two methods agree closely. The mean per-encounter switch-readiness probability is 0.0192 (product of marginals) versus 0.0191 (autoregressive joint), and the Spearman rank correlation across encounters is 0.86; the per-encounter scatter in Supplementary Figure 2 (left)

sits close to the equality line, indicating that the two probabilities track each other across the full distribution of encounters. Downstream ranking performance is shown in Supplementary Table 11: the product of marginals is at least as good as the autoregressive joint on AUROC, average precision and Brier score, with the difference statistically significant in its favour on all three under a paired bootstrap test. The calibration curves for the two methods (Supplementary Figure 2, right) overlap within their bootstrap bands.

We attribute the improved performance of the product of marginals method primarily to Monte Carlo noise in the autoregressive estimator: the switch-readiness event is rare, so a feasible number of joint samples yields noisy per-encounter estimates that shuffle the ordering on which ranking metrics depend, whereas the product of marginals is deterministic. Drawing enough samples to resolve probabilities of this magnitude precisely would also be very expensive: each autoregressive sample requires one forward pass per target point sequentially, and these cannot be vectorised in the same way as the single batched forward pass that produces the closed-form marginals.

For the bias direction, any positive correlation between vitals would cause the product of marginals to *underestimate* the joint probability, which is the conservative direction in this clinical setting: the system would tend to flag fewer patients as ready, rather than more.

Model	Pulse	Resp. rate	SpO_2	Systolic BP	Temp.
Repeat	9.17 [9.14–9.19]	4.09 [4.08–4.11]	2.05 [2.04–2.05]	14.29 [14.24–14.34]	0.63 [0.62–0.63]
GBDT	8.92 [8.90–8.94]	3.49 [3.48–3.50]	1.79 [1.78–1.79]	12.27 [12.24–12.30]	0.55 [0.55–0.55]
NP	**6.05 [6.03–6.07]**	**2.47 [2.46–2.47]**	**1.28 [1.27–1.28]**	**8.60 [8.57–8.63]**	**0.40 [0.40–0.40]**

Supplementary Table 12: Probabilistic forecasting performance on the MIMIC test set under CRPS. For the deterministic Repeat and GBDT baselines, CRPS reduces to MAE under their point-mass predictive distribution, so the values shown are equivalent to those in Table 2; for the NP, CRPS is computed in closed form from its Gaussian predictive distribution. 95% bootstrap CIs in brackets; bold marks the best score in each column. Lower is better.

CRPS evaluation

The forecasting comparison in the main text (Table 2) is reported using mean absolute error (MAE), which assesses only point predictions. Here we report the continuous ranked probability score (CRPS), a strictly proper scoring rule that integrates over the full predictive distribution and is jointly sensitive to calibration and sharpness [40]. For deterministic models, which place all probability mass on a single value, CRPS reduces exactly to MAE; the MAE figures already in Table 2 for the Repeat and GBDT baselines therefore serve as the appropriate reference. For the NP, whose predictive distribution at each target point is Gaussian with mean μ and standard deviation σ, CRPS admits the closed form

$$\mathrm{CRPS}\big(\mathcal{N}(\mu, \sigma^2),\, y\big) = \sigma \left[z\big(2\Phi(z) - 1\big) + 2\varphi(z) - \tfrac{1}{\sqrt{\pi}} \right], \qquad z = \frac{y - \mu}{\sigma}, \tag{14}$$

where Φ and φ are the standard normal CDF and PDF respectively and y is the observed value [40]. We evaluate this at each forecast time point and average across all five vital signs in the MIMIC test set. Across all five vital signs the NP's CRPS is substantially lower than the Repeat and GBDT MAEs, confirming that the NP's probabilistic forecasts remain meaningfully more informative than the deterministic baselines.

**Model**	K=1	K=2	K=3	K=5	K=10
Logistic	0.385 [0.346–0.423]	0.364 [0.339–0.391]	0.324 [0.302–0.347]	0.280 [0.263–0.297]	0.223 [0.211–0.235]
GBDT	0.554 [0.515–0.590]	0.501 [0.471–0.529]	0.443 [0.418–0.464]	0.381 [0.363–0.400]	0.275 [0.262–0.290]
NP	0.577 [0.536–0.614]	0.526 [0.494–0.554]	0.469 [0.443–0.495]	0.397 [0.377–0.417]	0.283 [0.269–0.298]
STraTS	0.593 [0.551–0.632]	0.541 [0.511–0.570]	0.481 [0.456–0.505]	0.394 [0.375–0.413]	0.287 [0.273–0.302]
Oracle	0.908 [0.886–0.931]	0.837 [0.810–0.862]	0.761 [0.735–0.788]	0.625 [0.597–0.654]	0.369 [0.350–0.390]

Supplementary Table 13: Precision@K on the MIMIC test set for $K \in \{1, 2, 3, 5, 10\}$. The Oracle row reports the per-day maximum $\min(1, n_+/K)$, the highest precision attainable given the daily positive count. Median and 95% bootstrap confidence intervals.

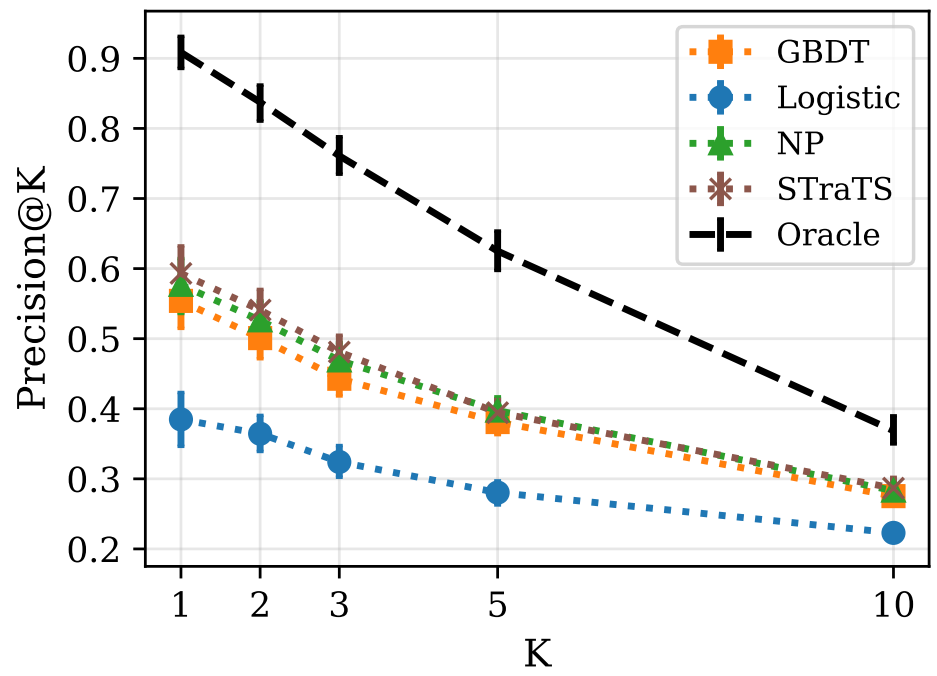

Supplementary Figure 3: Precision@K on the MIMIC test set as a function of K. The dashed line shows the per-day oracle, i.e. the highest precision attainable given the daily positive count. Curves show the median over bootstrap resamples of the test set and shaded regions show 95% bootstrap confidence intervals.

Precision@K across review-capacity values

The main text reports precision@5 as the deployment-aligned ranking metric, reflecting a daily review capacity of five patients. To assess sensitivity to this choice we compute precision@K for $K \in \{1, 2, 3, 5, 10\}$ on the MIMIC test set; results are shown in Supplementary Table 13 and Supplementary Figure 3. We additionally report a per-day oracle, the highest precision attainable on each day given the number of positive instances available; this provides a meaningful upper bound because precision@K is capped by the daily positive count.

The relative ordering of models is essentially stable across K: NP and STraTS lead at all values of K and are statistically indistinguishable from each other, the GBDT is a step behind, and the logistic baseline is consistently weakest. Absolute precision falls with K for all models, as the daily top-K is increasingly diluted by negatives once K exceeds the typical daily positive count, and this same dilution drives the oracle downwards too. NP-Tuned is omitted because the per-day predictions required for this analysis were not retained for that model.

Example encounters

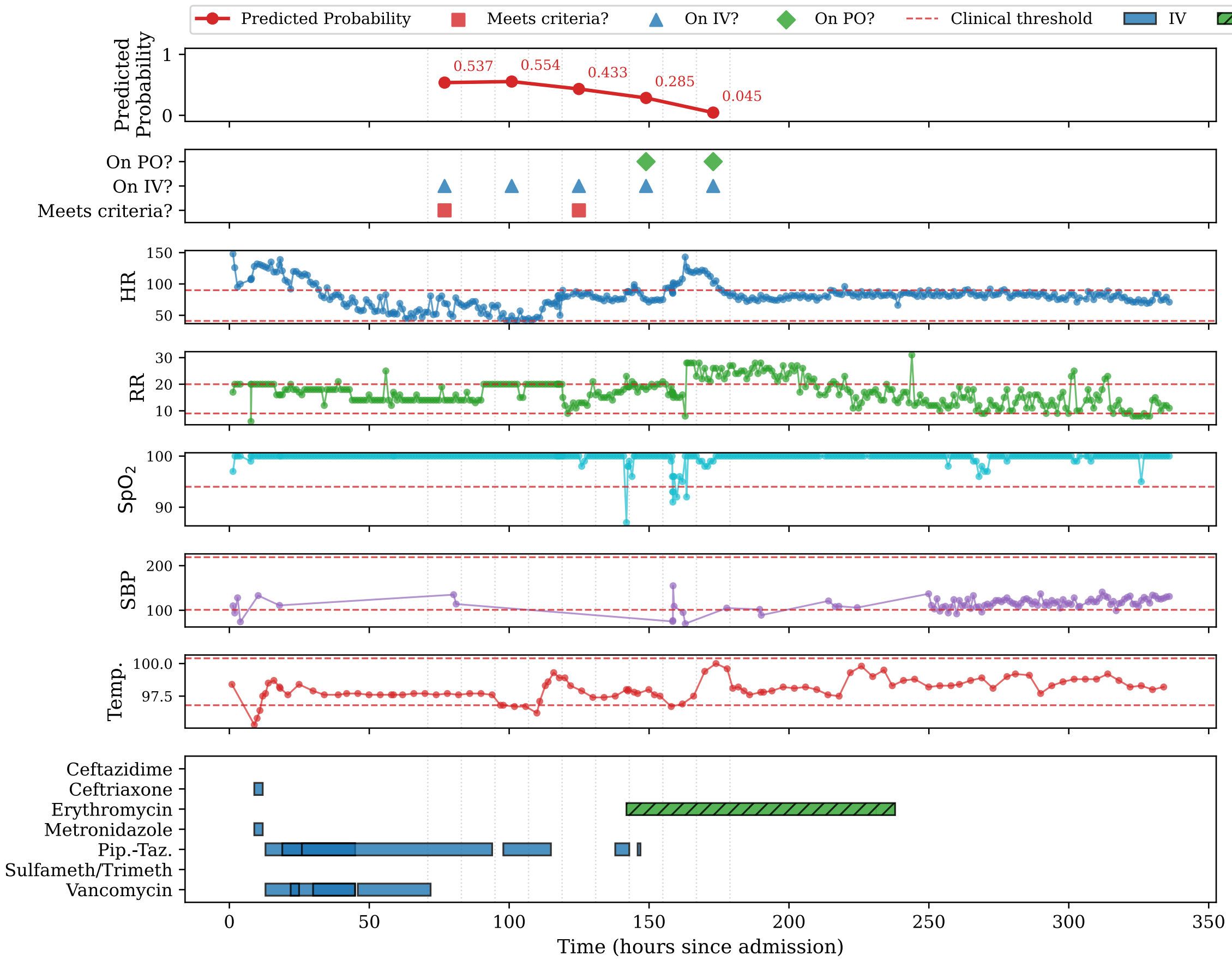

Supplementary Figure 4: Example encounter from the MIMIC test set illustrating a case in which the system's prediction is less clearly beneficial. The system recommends an earlier switch, but then the patient becomes more unwell as they are switched. In this encounter the test days only span around half of the full stay, since the IV prescription is halted after 150 hours. Panels show the same information as those in the figure in the main text. Variable Abbreviations: HR (Heart Rate), RR (Respiratory Rate), SpO_2 (Oxygen Saturation), SBP (Systolic Blood Pressure), Temp. (Temperature). Antibiotic Abbreviations: Pip.-Taz., Piperacillin-Tazobactam; Sulfameth/Trimeth, Sulfamethoxazole-Trimethoprim

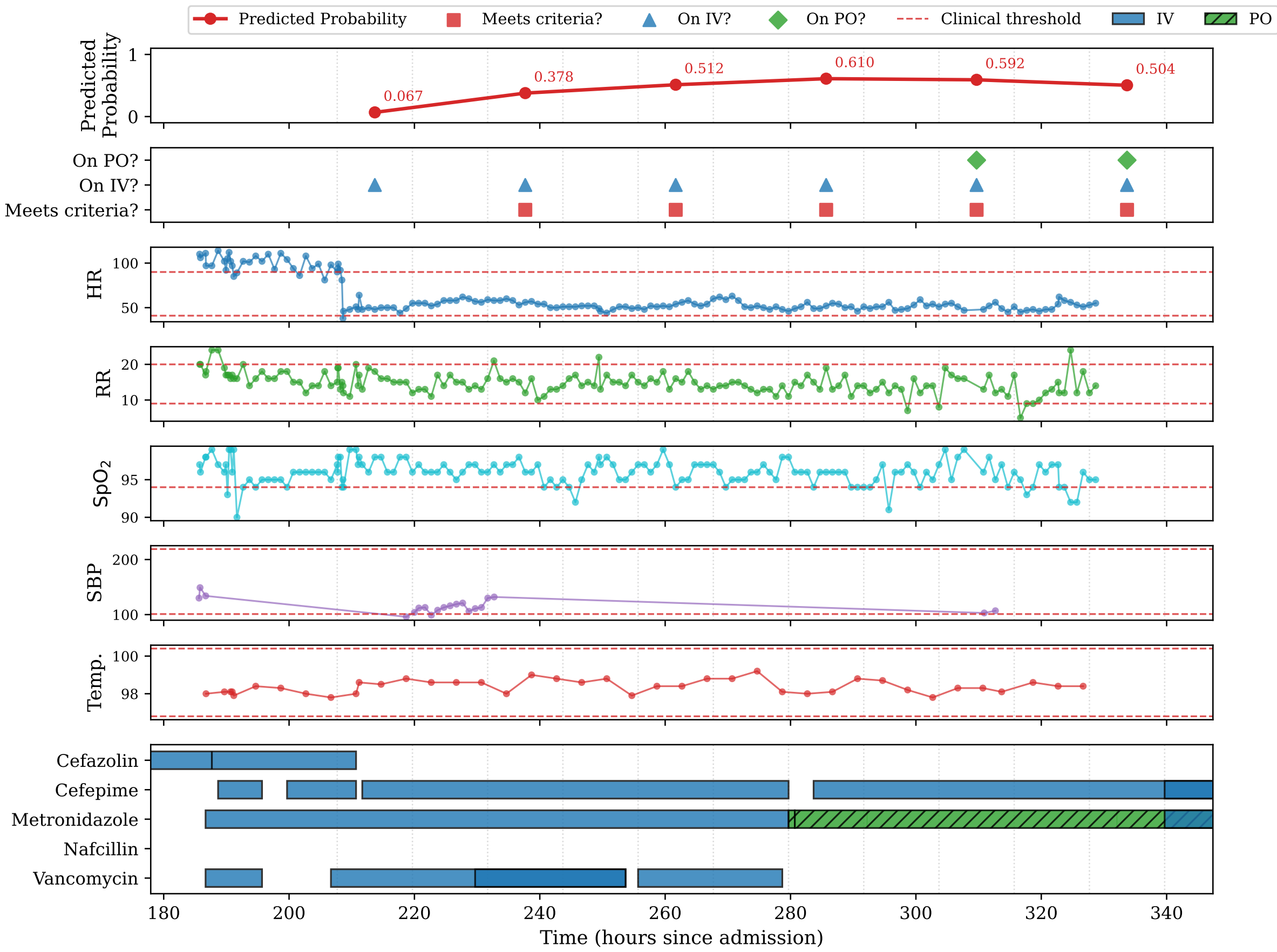

Supplementary Figure 5: Example encounter from the MIMIC test set. In this example the patient is unwell at the very start of the encounter, but their vitals are stable for most of the remainder, aside from a few spikes. The model becomes more confident the patient is ready to be switched as the encounter progresses, although no switch occurs. Panels show the same information as those in the figure in the main text. Variable Abbreviations: HR (Heart Rate), RR (Respiratory Rate), SpO_2 (Oxygen Saturation), SBP (Systolic Blood Pressure), Temp. (Temperature).

Supplementary Table 14: Test-set discrimination on MIMIC computed on the full test split ('Full') and the subset of test encounters whose patient does not appear in the training split ('Disjoint'). Median [95% bootstrap CI].

Model	Subset	AUROC	AP	Brier
Logistic	Full	0.759 [0.749–0.767]	0.300 [0.284–0.315]	0.098 [0.095–0.100]
	Disjoint	0.761 [0.751–0.771]	0.307 [0.290–0.325]	0.098 [0.095–0.101]
GBDT	Full	0.869 [0.863–0.875]	0.471 [0.453–0.490]	0.081 [0.079–0.084]
	Disjoint	0.868 [0.862–0.875]	0.467 [0.446–0.488]	0.082 [0.079–0.085]
NP	Full	0.882 [0.877–0.887]	0.477 [0.459–0.497]	0.106 [0.102–0.110]
	Disjoint	0.883 [0.877–0.889]	0.477 [0.457–0.499]	0.107 [0.103–0.111]
NP-Tuned	Full	0.856 [0.849–0.863]	0.444 [0.426–0.465]	0.083 [0.081–0.086]
	Disjoint	0.855 [0.848–0.862]	0.440 [0.420–0.461]	0.084 [0.082–0.087]
STraTS	Full	0.886 [0.881–0.892]	0.492 [0.472–0.513]	0.159 [0.156–0.162]
	Disjoint	0.887 [0.881–0.893]	0.491 [0.470–0.512]	0.159 [0.156–0.162]

Supplementary Note 3: Train/test patient overlap

Because the test set is constructed by a temporal cut on encounter admission dates, a small minority of patients can appear in both the training and test splits through separate admissions falling either side of the cut. To quantify the impact of this overlap on the reported test-set metrics we recompute discrimination on the subset of test encounters whose patient does not appear in the training split ("Disjoint"), and compare against the full test split ("Full"). The analysis is restricted to MIMIC, since UCLH patient identifiers were not exported alongside the prediction outputs for data governance reasons. For MIMIC 8.5% of unique test patients also appear in training, accounting for 10.9% of test encounters; the Full split contains 22,615 test encounters (2,761 switch-ready) and the Disjoint subset contains 20,154 (2,478 switch-ready). Supplementary Table 14 shows that all metrics on the Disjoint subset are within the bootstrap confidence intervals of the Full split for every model, indicating that the reported test-set performance is not driven by patient leakage across splits.

Supplementary Note 4: Subgroup analysis

We report AUROC, average precision, and Brier score stratified by age band, gender, ethnicity, and length-of-stay band on both datasets. Precision@5 is not included because it is a per-day metric defined over the daily ranked list of patients, and most subgroups do not contain enough patients on a given day for the top-5 cut to be a meaningful summary; the cohort-level metrics shown are robust to this and remain directly comparable to the main-text tables.

Supplementary Table 15: Subcohort AUROC metrics for MIMIC.

Variable	Level	n_{enc}	Logistic	GBDT	NP	NP-Tuned	STraTS
Age band	<40	26,327	0.781 [0.748–0.810]	0.880 [0.859–0.899]	0.893 [0.874–0.909]	0.870 [0.846–0.890]	0.899 [0.879–0.914]
	40–65	118,128	0.767 [0.754–0.781]	0.872 [0.863–0.882]	0.885 [0.877–0.894]	0.859 [0.849–0.869]	0.886 [0.877–0.895]
	65–80	111,715	0.741 [0.723–0.756]	0.865 [0.854–0.875]	0.879 [0.870–0.887]	0.849 [0.838–0.860]	0.886 [0.877–0.894]
	80+	53,096	0.770 [0.746–0.793]	0.864 [0.846–0.879]	0.878 [0.862–0.891]	0.862 [0.845–0.877]	0.883 [0.868–0.897]
Gender	F	128,060	0.770 [0.756–0.785]	0.869 [0.859–0.879]	0.882 [0.872–0.891]	0.852 [0.841–0.863]	0.884 [0.875–0.893]
	M	181,206	0.751 [0.739–0.762]	0.869 [0.861–0.877]	0.882 [0.875–0.890]	0.858 [0.850–0.867]	0.887 [0.881–0.895]
LOS band	<3d	1,149	0.801 [0.727–0.869]	0.841 [0.776–0.904]	0.845 [0.773–0.913]	0.826 [0.755–0.893]	0.857 [0.791–0.919]
	3–7d	34,367	0.743 [0.714–0.772]	0.844 [0.821–0.865]	0.854 [0.833–0.874]	0.838 [0.814–0.861]	0.861 [0.839–0.879]
	>7d	273,750	0.758 [0.748–0.768]	0.871 [0.865–0.877]	0.885 [0.879–0.891]	0.858 [0.850–0.864]	0.889 [0.883–0.894]
Race	Asian	9,569	0.775 [0.725–0.820]	0.867 [0.831–0.896]	0.866 [0.833–0.893]	0.839 [0.800–0.873]	0.889 [0.861–0.914]
	Black	33,147	0.775 [0.748–0.800]	0.890 [0.874–0.905]	0.897 [0.881–0.912]	0.870 [0.853–0.888]	0.906 [0.892–0.920]
	Hispanic	12,127	0.758 [0.711–0.803]	0.894 [0.865–0.918]	0.916 [0.894–0.936]	0.891 [0.864–0.916]	0.908 [0.883–0.930]
	Other	14,336	0.744 [0.698–0.786]	0.867 [0.840–0.893]	0.868 [0.840–0.895]	0.852 [0.820–0.880]	0.876 [0.849–0.902]
	Unknown	37,431	0.744 [0.720–0.767]	0.859 [0.843–0.874]	0.880 [0.866–0.893]	0.846 [0.828–0.862]	0.882 [0.868–0.896]
	White	201,355	0.760 [0.747–0.770]	0.866 [0.858–0.874]	0.878 [0.871–0.885]	0.856 [0.847–0.863]	0.882 [0.875–0.889]

Supplementary Table 16: Subcohort average precision (AP) metrics for MIMIC.

Variable	Level	n_{enc}	Logistic	GBDT	NP	NP-Tuned	STraTS
Age band	<40	26,327	0.314 [0.256–0.372]	0.474 [0.405–0.546]	0.496 [0.420–0.563]	0.451 [0.374–0.517]	0.517 [0.438–0.583]
	40–65	118,128	0.313 [0.289–0.340]	0.480 [0.451–0.513]	0.498 [0.466–0.531]	0.458 [0.428–0.489]	0.504 [0.470–0.536]
	65–80	111,715	0.283 [0.259–0.309]	0.453 [0.419–0.486]	0.451 [0.420–0.482]	0.424 [0.394–0.455]	0.470 [0.438–0.504]
	80+	53,096	0.315 [0.276–0.360]	0.491 [0.438–0.545]	0.502 [0.451–0.553]	0.475 [0.421–0.525]	0.512 [0.461–0.561]
Gender	F	128,060	0.301 [0.275–0.326]	0.460 [0.429–0.491]	0.454 [0.424–0.487]	0.422 [0.391–0.453]	0.469 [0.436–0.501]
	M	181,206	0.300 [0.280–0.320]	0.476 [0.451–0.501]	0.494 [0.470–0.520]	0.460 [0.435–0.487]	0.507 [0.482–0.533]
LOS band	<3d	1,149	0.582 [0.438–0.740]	0.741 [0.608–0.848]	0.727 [0.581–0.841]	0.659 [0.507–0.802]	0.729 [0.584–0.855]
	3–7d	34,367	0.373 [0.316–0.431]	0.509 [0.445–0.569]	0.484 [0.422–0.552]	0.477 [0.413–0.538]	0.519 [0.456–0.579]
	>7d	273,750	0.289 [0.272–0.306]	0.462 [0.439–0.483]	0.475 [0.454–0.495]	0.438 [0.416–0.458]	0.484 [0.462–0.504]
Race	Asian	9,569	0.311 [0.241–0.391]	0.474 [0.367–0.570]	0.468 [0.376–0.565]	0.465 [0.365–0.552]	0.491 [0.394–0.584]
	Black	33,147	0.329 [0.278–0.379]	0.516 [0.460–0.572]	0.537 [0.473–0.595]	0.498 [0.439–0.555]	0.547 [0.486–0.608]
	Hispanic	12,127	0.298 [0.226–0.381]	0.517 [0.417–0.613]	0.529 [0.435–0.625]	0.487 [0.389–0.592]	0.502 [0.406–0.603]
	Other	14,336	0.308 [0.240–0.381]	0.439 [0.359–0.521]	0.468 [0.377–0.556]	0.427 [0.344–0.514]	0.462 [0.376–0.546]
	Unknown	37,431	0.255 [0.217–0.290]	0.428 [0.379–0.475]	0.461 [0.413–0.511]	0.413 [0.365–0.462]	0.475 [0.427–0.523]
	White	201,355	0.311 [0.290–0.332]	0.475 [0.449–0.503]	0.470 [0.444–0.494]	0.443 [0.418–0.466]	0.490 [0.464–0.514]

Supplementary Table 17: Subcohort Brier score metrics for MIMIC.

Variable	Level	n_{enc}	Logistic	GBDT	NP	NP-Tuned	STraTS
Age band	<40	26,327	0.089 [0.080–0.099]	0.075 [0.067–0.083]	0.098 [0.086–0.110]	0.076 [0.068–0.085]	0.141 [0.132–0.152]
	40–65	118,128	0.101 [0.096–0.106]	0.084 [0.080–0.088]	0.112 [0.105–0.118]	0.087 [0.083–0.091]	0.160 [0.155–0.166]
	65–80	111,715	0.096 [0.091–0.101]	0.080 [0.076–0.084]	0.102 [0.097–0.109]	0.082 [0.079–0.086]	0.161 [0.157–0.166]
	80+	53,096	0.098 [0.091–0.104]	0.083 [0.076–0.088]	0.107 [0.098–0.116]	0.082 [0.076–0.089]	0.162 [0.154–0.170]
Gender	F	128,060	0.094 [0.090–0.099]	0.079 [0.076–0.083]	0.103 [0.097–0.109]	0.082 [0.078–0.086]	0.160 [0.155–0.165]
	M	181,206	0.100 [0.096–0.103]	0.083 [0.080–0.086]	0.108 [0.104–0.113]	0.084 [0.081–0.087]	0.159 [0.155–0.163]
LOS band	<3d	1,149	0.198 [0.161–0.241]	0.165 [0.128–0.204]	0.287 [0.221–0.356]	0.173 [0.137–0.214]	0.210 [0.165–0.256]
	3–7d	34,367	0.110 [0.101–0.121]	0.097 [0.088–0.105]	0.128 [0.115–0.142]	0.097 [0.088–0.106]	0.184 [0.173–0.196]
	>7d	273,750	0.096 [0.093–0.098]	0.079 [0.077–0.082]	0.103 [0.099–0.106]	0.081 [0.079–0.084]	0.157 [0.153–0.160]
Race	Asian	9,569	0.104 [0.089–0.120]	0.088 [0.075–0.102]	0.114 [0.093–0.134]	0.090 [0.076–0.106]	0.149 [0.133–0.166]
	Black	33,147	0.094 [0.086–0.101]	0.077 [0.070–0.083]	0.102 [0.091–0.112]	0.079 [0.073–0.086]	0.144 [0.136–0.153]
	Hispanic	12,127	0.093 [0.080–0.108]	0.074 [0.064–0.086]	0.100 [0.081–0.117]	0.075 [0.063–0.087]	0.146 [0.133–0.163]
	Other	14,336	0.099 [0.086–0.112]	0.084 [0.073–0.095]	0.108 [0.091–0.125]	0.086 [0.075–0.097]	0.156 [0.144–0.170]
	Unknown	37,431	0.088 [0.082–0.095]	0.075 [0.070–0.081]	0.094 [0.085–0.102]	0.076 [0.071–0.083]	0.150 [0.143–0.157]
	White	201,355	0.101 [0.097–0.105]	0.084 [0.081–0.087]	0.111 [0.106–0.116]	0.086 [0.083–0.090]	0.168 [0.163–0.172]

Supplementary Table 18: Subcohort positive-class prevalence for MIMIC.

Variable	Level	n_{enc}	n_{pos}	Positive rate
Age band	<40	2,009	226	11.2%
	40–65	8,045	1,034	12.9%
	65–80	9,113	1,071	11.8%
	80+	3,448	430	12.5%
Gender	F	9,078	1,069	11.8%
	M	13,537	1,692	12.5%
LOS band	<3d	147	48	32.7%
	3–7d	1,960	287	14.6%
	>7d	20,508	2,426	11.8%
Race	Asian	752	99	13.2%
	Black	2,712	327	12.1%
	Hispanic	947	111	11.7%
	Other	1,089	134	12.3%
	Unknown	4,495	478	10.6%
	White	12,546	1,597	12.7%

Supplementary Table 19: Subcohort AUROC metrics for UCLH.

Variable	Level	n_{enc}	Logistic	GBDT	NP	NP-Tuned	STraTS
Age band	<40	39,750	0.825 [0.816–0.834]	0.848 [0.840–0.856]	0.852 [0.845–0.860]	0.856 [0.848–0.864]	0.859 [0.851–0.867]
	40–65	77,460	0.829 [0.823–0.835]	0.863 [0.857–0.868]	0.858 [0.852–0.864]	0.868 [0.862–0.873]	0.870 [0.864–0.875]
	65–80	66,207	0.833 [0.826–0.841]	0.870 [0.864–0.877]	0.864 [0.858–0.870]	0.874 [0.868–0.880]	0.877 [0.871–0.883]
	80+	25,945	0.809 [0.798–0.821]	0.856 [0.846–0.866]	0.851 [0.840–0.861]	0.864 [0.853–0.872]	0.865 [0.855–0.874]
Gender	Female	96,969	0.823 [0.817–0.829]	0.858 [0.852–0.863]	0.855 [0.850–0.860]	0.865 [0.860–0.870]	0.868 [0.862–0.872]
	Male	112,388	0.831 [0.826–0.837]	0.865 [0.860–0.870]	0.860 [0.855–0.864]	0.869 [0.864–0.874]	0.872 [0.867–0.876]
LOS band	<3d	2,227	0.763 [0.721–0.798]	0.778 [0.737–0.814]	0.793 [0.752–0.830]	0.801 [0.763–0.835]	0.809 [0.774–0.842]
	3–7d	23,953	0.796 [0.787–0.806]	0.829 [0.819–0.838]	0.828 [0.819–0.837]	0.837 [0.827–0.845]	0.841 [0.831–0.849]
	>7d	183,182	0.830 [0.825–0.834]	0.865 [0.861–0.869]	0.859 [0.855–0.863]	0.870 [0.866–0.874]	0.872 [0.868–0.876]
Race	Asian	16,745	0.818 [0.805–0.831]	0.845 [0.833–0.857]	0.852 [0.839–0.863]	0.855 [0.843–0.867]	0.858 [0.845–0.869]
	Black	16,626	0.827 [0.814–0.841]	0.862 [0.849–0.874]	0.859 [0.846–0.870]	0.867 [0.855–0.878]	0.871 [0.859–0.882]
	Other	53,599	0.832 [0.824–0.840]	0.865 [0.858–0.872]	0.860 [0.852–0.866]	0.870 [0.863–0.876]	0.874 [0.867–0.880]
	Unknown	26,376	0.827 [0.817–0.838]	0.862 [0.852–0.871]	0.857 [0.847–0.866]	0.866 [0.856–0.875]	0.868 [0.859–0.877]
	White	96,016	0.825 [0.819–0.831]	0.862 [0.857–0.868]	0.857 [0.852–0.862]	0.868 [0.863–0.873]	0.870 [0.865–0.875]

Supplementary Table 20: Subcohort average precision (AP) metrics for UCLH.

Variable	Level	n_{enc}	Logistic	GBDT	NP	NP-Tuned	STraTS
Age band	<40	39,750	0.723 [0.706–0.741]	0.765 [0.749–0.781]	0.772 [0.757–0.788]	0.773 [0.759–0.790]	0.781 [0.766–0.796]
	40–65	77,460	0.727 [0.714–0.739]	0.782 [0.771–0.793]	0.773 [0.761–0.784]	0.789 [0.778–0.800]	0.794 [0.782–0.804]
	65–80	66,207	0.762 [0.749–0.775]	0.813 [0.802–0.824]	0.802 [0.790–0.813]	0.817 [0.806–0.828]	0.820 [0.808–0.831]
	80+	25,945	0.746 [0.727–0.767]	0.817 [0.801–0.833]	0.802 [0.786–0.818]	0.824 [0.808–0.838]	0.828 [0.812–0.842]
Gender	Female	96,969	0.725 [0.713–0.735]	0.781 [0.770–0.791]	0.778 [0.766–0.787]	0.791 [0.780–0.800]	0.796 [0.785–0.804]
	Male	112,388	0.752 [0.742–0.763]	0.805 [0.796–0.814]	0.792 [0.783–0.802]	0.808 [0.799–0.817]	0.813 [0.804–0.822]
LOS band	<3d	2,227	0.876 [0.844–0.902]	0.883 [0.852–0.907]	0.886 [0.853–0.913]	0.893 [0.863–0.919]	0.903 [0.875–0.925]
	3–7d	23,953	0.804 [0.792–0.816]	0.838 [0.826–0.849]	0.835 [0.824–0.847]	0.846 [0.836–0.857]	0.848 [0.838–0.859]
	>7d	183,182	0.713 [0.703–0.722]	0.774 [0.766–0.782]	0.761 [0.754–0.770]	0.779 [0.772–0.787]	0.784 [0.776–0.791]
Race	Asian	16,745	0.750 [0.725–0.773]	0.787 [0.764–0.811]	0.802 [0.780–0.821]	0.802 [0.778–0.822]	0.802 [0.779–0.823]
	Black	16,626	0.729 [0.704–0.755]	0.786 [0.763–0.808]	0.778 [0.753–0.801]	0.791 [0.768–0.812]	0.797 [0.772–0.818]
	Other	53,599	0.733 [0.716–0.748]	0.780 [0.766–0.793]	0.767 [0.753–0.782]	0.784 [0.771–0.798]	0.791 [0.779–0.805]
	Unknown	26,376	0.717 [0.697–0.738]	0.778 [0.760–0.797]	0.764 [0.743–0.784]	0.782 [0.763–0.801]	0.787 [0.768–0.805]
	White	96,016	0.749 [0.738–0.759]	0.808 [0.798–0.817]	0.799 [0.789–0.808]	0.814 [0.805–0.822]	0.818 [0.809–0.827]

Supplementary Table 21: Subcohort Brier score metrics for UCLH.

Variable	Level	n_{enc}	Logistic	GBDT	NP	NP-Tuned	STraTS
Age band	<40	39,750	0.169 [0.164–0.173]	0.155 [0.151–0.160]	0.213 [0.207–0.220]	0.152 [0.148–0.156]	0.161 [0.156–0.166]
	40–65	77,460	0.164 [0.161–0.167]	0.147 [0.143–0.150]	0.206 [0.201–0.211]	0.144 [0.141–0.147]	0.157 [0.154–0.161]
	65–80	66,207	0.166 [0.163–0.169]	0.145 [0.141–0.149]	0.218 [0.212–0.224]	0.143 [0.139–0.146]	0.153 [0.150–0.158]
	80+	25,945	0.177 [0.172–0.183]	0.154 [0.149–0.160]	0.242 [0.234–0.251]	0.150 [0.145–0.156]	0.160 [0.154–0.167]
Gender	Female	96,969	0.168 [0.166–0.171]	0.150 [0.147–0.153]	0.212 [0.207–0.216]	0.146 [0.144–0.149]	0.158 [0.155–0.162]
	Male	112,388	0.166 [0.164–0.169]	0.148 [0.145–0.150]	0.219 [0.215–0.223]	0.145 [0.143–0.148]	0.156 [0.153–0.159]
LOS band	<3d	2,227	0.202 [0.190–0.216]	0.177 [0.165–0.193]	0.323 [0.300–0.346]	0.171 [0.157–0.186]	0.152 [0.136–0.169]
	3–7d	23,953	0.190 [0.185–0.194]	0.169 [0.164–0.173]	0.262 [0.255–0.269]	0.165 [0.161–0.170]	0.165 [0.160–0.170]
	>7d	183,182	0.161 [0.159–0.163]	0.144 [0.141–0.146]	0.202 [0.199–0.206]	0.141 [0.139–0.143]	0.155 [0.153–0.158]
Race	Asian	16,745	0.175 [0.169–0.181]	0.162 [0.155–0.169]	0.226 [0.216–0.238]	0.156 [0.149–0.163]	0.160 [0.153–0.168]
	Black	16,626	0.167 [0.160–0.173]	0.148 [0.141–0.155]	0.211 [0.201–0.222]	0.145 [0.139–0.152]	0.155 [0.148–0.163]
	Other	53,599	0.161 [0.157–0.165]	0.144 [0.141–0.148]	0.202 [0.196–0.208]	0.142 [0.138–0.146]	0.155 [0.151–0.159]
	Unknown	26,376	0.164 [0.159–0.169]	0.147 [0.141–0.152]	0.204 [0.196–0.212]	0.145 [0.140–0.150]	0.160 [0.154–0.166]
	White	96,016	0.171 [0.168–0.173]	0.150 [0.147–0.153]	0.225 [0.221–0.230]	0.147 [0.144–0.150]	0.157 [0.154–0.160]

Supplementary Table 22: Subcohort positive-class prevalence for UCLH.

Variable	Level	n_{enc}	n_{pos}	Positive rate
Age band	<40	8,085	3,196	39.5%
	40–65	15,714	6,032	38.4%
	65–80	12,161	5,158	42.4%
	80+	5,256	2,370	45.1%
Gender	Female	19,702	7,761	39.4%
	Male	21,510	8,991	41.8%
LOS band	<3d	675	474	70.2%
	3–7d	7,710	4,256	55.2%
	>7d	32,831	12,026	36.6%
Race	Asian	3,508	1,536	43.8%
	Black	3,304	1,323	40.0%
	Other	10,269	3,835	37.3%
	Unknown	5,603	2,125	37.9%
	White	18,532	7,937	42.8%
