# Supplementary material for: Optimising antibiotic switching via forecasting of patient physiology
Source: Nat Commun. 2026 Aug 25;17:10475. doi: 10.1038/s41467-026-76715-w (PMC13635169; doi:10.1038/s41467-026-76715-w)
Supplement: Supplementary file 2 [file 41467_2026_76715_MOESM2_ESM.pdf]

nature portfolio

Corresponding author(s): Magnus Ross

Last updated by author(s): Aug 3, 2026

Reporting Summary

Nature Portfolio wishes to improve the reproducibility of the work that we publish. This form provides structure for consistency and transparency in reporting. For further information on Nature Portfolio policies, see our Editorial Policies and the Editorial Policy Checklist.

Statistics

For all statistical analyses, confirm that the following items are present in the figure legend, table legend, main text, or Methods section.

n/a	Confirmed	
☐	☒	The exact sample size (*n*) for each experimental group/condition, given as a discrete number and unit of measurement
☐	☒	A statement on whether measurements were taken from distinct samples or whether the same sample was measured repeatedly
☐	☒	The statistical test(s) used AND whether they are one- or two-sided<br>*Only common tests should be described solely by name; describe more complex techniques in the Methods section.*
☐	☒	A description of all covariates tested
☐	☒	A description of any assumptions or corrections, such as tests of normality and adjustment for multiple comparisons
☐	☒	A full description of the statistical parameters including central tendency (e.g. means) or other basic estimates (e.g. regression coefficient) AND variation (e.g. standard deviation) or associated estimates of uncertainty (e.g. confidence intervals)
☐	☒	For null hypothesis testing, the test statistic (e.g. *F*, *t*, *r*) with confidence intervals, effect sizes, degrees of freedom and *P* value noted<br>*Give P values as exact values whenever suitable.*
☒	☐	For Bayesian analysis, information on the choice of priors and Markov chain Monte Carlo settings
☒	☐	For hierarchical and complex designs, identification of the appropriate level for tests and full reporting of outcomes
☐	☒	Estimates of effect sizes (e.g. Cohen's *d*, Pearson's *r*), indicating how they were calculated

Our web collection on statistics for biologists contains articles on many of the points above.

Software and code

Policy information about availability of computer code

Data collection	Data is from the publicly available MIMIC-IV data set, and a non-public extract from the ULCH NHS trust.
Data analysis	Custom code was developed for data analysis and is available at https://github.com/SAFEHR-data/ivos-model-public (archived at https://doi.org/10.5281/zenodo.21337708). Analyses used Python 3.12 with neuralprocesses 0.2.7 on PyTorch 2.8.0, scikit-learn 1.7.2, LightGBM 4.6.0, pandas 2.3.2, NumPy 2.3.3, SciPy 1.16.1, Matplotlib 3.10.6, seaborn 0.13.2 and MLflow 3.5.1. The analysis depends on a larger set of packages than can reasonably be listed; the complete set with exact pinned versions is given in the lock file in the repository.

For manuscripts utilizing custom algorithms or software that are central to the research but not yet described in published literature, software must be made available to editors and reviewers. We strongly encourage code deposition in a community repository (e.g. GitHub). See the Nature Portfolio guidelines for submitting code & software for further information.

Data

Policy information about availability of data

All manuscripts must include a data availability statement. This statement should provide the following information, where applicable:

- Accession codes, unique identifiers, or web links for publicly available datasets
- A description of any restrictions on data availability
- For clinical datasets or third party data, please ensure that the statement adheres to our policy

The MIMIC-IV dataset can be accessed at https://physionet.org/content/mimiciv/3.1/ The UCLH dataset contains personally identifiable data that has not been approved for external release. Researchers wishing to validate or replicate the results on the UCLH dataset would need to be approved for research collaborations with University College London Hospitals NHS Foundation Trust, and to secure appropriate permissions from the UCLH/UCL Joint Research Office. Researchers who meet these requirements can contact the corresponding author for further information about access to the datasets.

Research involving human participants, their data, or biological material

Policy information about studies with human participants or human data. See also policy information about sex, gender (identity/presentation), and sexual orientation and race, ethnicity and racism.

Reporting on sex and gender	Included in table 1
Reporting on race, ethnicity, or other socially relevant groupings	Included in table 1
Population characteristics	Included in table 1
Recruitment	Included
Ethics oversight	This study is a retrospective analysis of de-identified, routinely collected data, with no prospective recruitment and no intervention. Use of the UCLH data was approved by the UCLH Data Access Process for Research (DAP-R), following review by the Research Data Access Committee (RDAC), under the "University College London Hospitals NHS Foundation Trust Data Only Studies" approval (REC reference 21/SW/0089; IRAS project ID 299136). Individual informed consent was not required, as the study used de-identified data collected in the course of routine clinical care. The MIMIC-IV data are publicly available in de-identified form and were collected under a waiver of informed consent granted by the Institutional Review Board of the Beth Israel Deaconess Medical Center.

Note that full information on the approval of the study protocol must also be provided in the manuscript.

Field-specific reporting

Please select the one below that is the best fit for your research. If you are not sure, read the appropriate sections before making your selection.

☒ Life sciences ☐ Behavioural & social sciences ☐ Ecological, evolutionary & environmental sciences

For a reference copy of the document with all sections, see nature.com/documents/nr-reporting-summary-flat.pdf

Life sciences study design

All studies must disclose on these points even when the disclosure is negative.

Sample size	6,333 unique admissions for the MIMIC dataset, 10,584 for the UCLH dataset. No statistical method was used to predetermine sample size. Both cohorts comprise every hospital encounter in the source database meeting the pre-established inclusion criteria, so the sample size is determined by the available data rather than chosen.
Data exclusions	Exclusions were pre-established and are described in the Methods (Data). Physiologically implausible measurements were dropped; encounters were truncated to a maximum of two weeks; patients aged under 18 were excluded (paediatric patients are already absent from MIMIC); patients aged over 90 were removed as a condition of the pseudonymisation required to extract the UCLH data; and encounters without any IV antibiotic prescription were excluded as they fall outside the IVOS population. No other data were excluded.
Replication	Described in the methods. Code available.
Randomization	A temporal split was used to assign encounters to the test set, in order to prevent temporal information leakage which could artificially inflate performance.
Blinding	Blinding is not relevant to this study. It is a retrospective computational analysis with no experimental groups and no intervention: switch-readiness labels are derived automatically by applying fixed clinical criteria to recorded vital signs, and all metrics are computed programmatically, so investigator judgement does not enter data collection or outcome assessment.

Reporting for specific materials, systems and methods

We require information from authors about some types of materials, experimental systems and methods used in many studies. Here, indicate whether each material, system or method listed is relevant to your study. If you are not sure if a list item applies to your research, read the appropriate section before selecting a response.

Materials & experimental systems

n/a	Involved in the study
☒	☐ Antibodies
☒	☐ Eukaryotic cell lines
☒	☐ Palaeontology and archaeology
☒	☐ Animals and other organisms
☒	☐ Clinical data
☒	☐ Dual use research of concern
☒	☐ Plants

Methods

n/a	Involved in the study
☒	☐ ChIP-seq
☒	☐ Flow cytometry
☒	☐ MRI-based neuroimaging

Plants

Seed stocks

Report on the source of all seed stocks or other plant material used. If applicable, state the seed stock centre and catalogue number. If plant specimens were collected from the field, describe the collection location, date and sampling procedures.

Novel plant genotypes

Describe the methods by which all novel plant genotypes were produced. This includes those generated by transgenic approaches, gene editing, chemical/radiation-based mutagenesis and hybridization. For transgenic lines, describe the transformation method, the number of independent lines analyzed and the generation upon which experiments were performed. For gene-edited lines, describe the editor used, the endogenous sequence targeted for editing, the targeting guide RNA sequence (if applicable) and how the editor was applied.

Authentication

Describe any authentication procedures for each seed stock used or novel genotype generated. Describe any experiments used to assess the effect of a mutation and, where applicable, how potential secondary effects (e.g. second site T-DNA insertions, mosiacism, off-target gene editing) were examined.
