# Supplementary material for: Optimising antibiotic switching via forecasting of patient physiology
Source: Nat Commun. 2026 Aug 25;17:10475. doi: 10.1038/s41467-026-76715-w (PMC13635169; doi:10.1038/s41467-026-76715-w)
Supplement: Supplementary file 3 [file 41467_2026_76715_MOESM3_ESM.pdf]

nature portfolio

Peer Review File

Optimising antibiotic switching via forecasting of patient physiology

Corresponding Author: Dr Magnus Ross

This file contains all reviewer reports in order by version, followed by all author rebuttals in order by version.

Version 0:

Reviewer comments:

Reviewer #1

(Remarks to the Author)
I read with interest the retrospective study by Magnus Ross and colleagues entitled “Optimising antibiotic switching via forecasting of patient physiology”. Please find below some major and minor comments on its technical merits.

Major comments

1) Page 2, lines 44-48: “However, fixed rules struggle to assess whether a patient is improving rather than merely meeting thresholds at one point in time. AI enabled methods that learn from historical switching behavior face a deeper limitation: if the training signal is what clinicians did, then the best the model can do is reproduce that sub-optimal behavior. It cannot reliably improve practice against a clinical standard.” I am not totally convinced by this justification. Certainly, I agree that learning from historical switching (clinicians’ decisions, including suboptimal ones) is a major limitation and that this may have occurred in the previous articles cited by the authors. However, simply learning from all historical decisions is not the rule in well-designed prediction studies that lead to the development of scores for guiding decision-making in healthcare, including switch to oral antibiotics. There are proper corrections. For example, scores may stem from studies looking at patients with switching at a given time that would be considered appropriate, vital signs at the time of switching or trend of vital signs in a time window preceding the time of switching, and subsequent relevant clinical outcomes (cure, recurrent infection, etc). This would allow to identify vital signs values associated with safe switching without relying on biased historical decisions (i.e., those suboptimal and not in line with guidelines). It may be a design issue rather than a model issue, in my opinion.

2) Page 2, lines 40-41: “The goal of a CDSS is therefore not to automate IVOS, but to surface patients for expert review and discussion with the primary team.” This is in line with page 2, lines 56-58: “The system ranks patients by predicted switch-readiness, helping teams prioritise reviews while preserving clinical judgement for factors outside routine vital signs data”. In my opinion, this is a correct idea and consideration. However, also the related limitations should be acknowledged, based on the study results. In this regard, there are two possible concerns, in my opinion. The first (least important) is the suboptimal recall, especially for the MIMIC dataset (ranging from 0.564 to 0.691 for the different model, including also NP and NP-tuned, as shown in Table 4). This means that at least 3 out of 10 patients who are ready to switch would be false negatives or, perhaps better, would show a low probability of readiness to switch despite they would be perfectly fit, meaning that they would not be proposed to expert clinicians for evaluation while conversely they could be readily intercepted by standard non-AI-supported approach (the cost-benefit of this possible scenario should be evaluated with dedicated future analyses). Anyway, this first concern seems to be less important clinically (although, of course, it is important from a antibiotic stewardship perspective) since the patient with true infection would continue to be treated with appropriate IV antibiotics (i.e., no expected changes in efficacy for the given treated infection). The second, more important concern regards the suboptimal precision (PPV) also in the UCLH cohort (the one in which the forecasting model seem to perform better), with a maximum of 0.667 for GBDT and 0.660 for NP-tuned. This means again that at least 3 out of 10 positives (i.e., classified as ready to switch by the model) would not be, in reality, ready to switch, and a switch to oral therapy could be deleterious in terms of efficacy. Certainly, the authors are right that this is exactly the scenario in which the expert review should intervene and that prospective studies are needed to validate this strategy (as they correctly discuss in the study limitations paragraph). However, they miss to discuss, in my opinion, also the possible impact of the forecasting model on expert decisions. Indeed, 3 out of 10 is a non-negligible frequency, and knowing this could, based on different users, either increase skepticism (high rate of not followed suggestions) or promote excessive reliance on suggestions by early

adopters/clinicians unawarely favouring the technology due to baseline positive attitude toward technology, or also increasing automation bias with use (leading to perilous switches in false positives). For these reasons, not only the validation should be prospective (a term which includes also observational designs) but also randomized (implementation vs. not implementation by proper RCT design) and should take into account clinicians' attitudes toward acting or not acting according to the proposed ranking of readiness to switch (in the implementation arm).

3) In the previous comment, I used the term "true infection" (although technically "proven infection" would be more terminologically correct). Moving this consideration to the denominator of the study (study population) this can bring the attention on another crucial point, in my opinion. The study population seem to be composed by patients in the two datasets who received intravenous antibiotics. However, antibiotics may be administered also empirically, due to the suspicion of infection, in patients who are later proved not to have an infection but another disease. This is not an uncommon situation and, in these patients, any improvement in vital signs is inherently not dependent on antibiotic therapy. Of course, it can be argued that the model may works also in these patients (by suggesting, instead that they are ready to switch, that they are ready to discontinue antibiotics). However, this has to be supported by data, since it is not automatic that vital signs/trend of vital signs can similarly forecast switching to oral antibiotics in patients receiving antibiotics for a proven infection and discontinuation of antibiotics in patients receiving empirical antibiotics but later found not to have an infection. If presence/absence of a proven infection is an information available in the MIMIC and UCLH datasets I'd strongly encourage the authors to perform such a subgroup analysis. In the subgroup of patients with proven infection please also report the type and site of infection, and the etiological agent whenever available. Regarding this latter point, the availability of the etiological agent and the related susceptibility test results would also allow to create a further subgroup of patients with infection in which the administered antibiotic is appropriate (based on expected activity given the susceptibility of the etiological agent to the administered antibiotic), although a complete assessment would also require clinicians labelling whether the PK of the antibiotic in the site of infection is appropriate and the dosage is correct. All of this is likely too much granular to be explored, nonetheless conducting subgroup analysis at least stratifying appropriateness by susceptibility test results could significantly improve the generalization potential of results, in my opinion.

4) Page 5, lines 95-97: "As the horizon increases the performance of the GBDT and NP converge, with the models providing similar performance on the 1 day horizon." This result may be problematic to externally validate the proposed model considering that it seems to work better in the UCLH cohort which includes less severe patients than in the MIMIC cohort (including more severe ICU patients), with consequent less frequent measurements. While this may not be the specific case of the UCLH cohort if a high number of availability of within 12-hour horizon measurements to properly train the model, this may not be the case for other cohorts of non-critically ill patients in which only daily measurements may be available and thus proper validation could be provided only for the 24-hour horizon (unless the validation is prospective through assessment of clinical outcomes after the switch decisions are supported by the 12-hour forecasting trained on the UCLH cohort). In other words, other retrospective studies in other non-severe cohorts to externally validate the 12-hour horizon forecasting ability may not be easy to conduct.

5) Another problem related to non-ICU cohort (i.e., those with less severe patients like the UCLH on, in which the model eventually seems to forecast better) regards the training of the model, and involves missing values. Consider page 14, lines 365-368: "A task is created on a given day only if the patient is considered to be on an IV prescription at the prediction time, there are at least 10 measurements in the preceding 48-hour look-back window, and there is at least one measurement in the 12-hour prediction window (9 am to 9 pm)"; and page 15, lines 414-416: "This choice to treat missing values as meeting criteria, rather than violating, is a modelling decision that must be clearly communicated to end users; the alternative would result in substantially more imbalanced class distributions, since missing data is common." The fact that non-severe cohorts may have more missing values may further impact on the generalization potential of results, further stressing the need for external validation in other non-severe cohorts (see previous comment).

6) Page 11, lines 239-245: "The separation between forecasting and application of criteria allows the model to be used much more flexibly in deployment. Take the example of a patient on mechanical ventilation, which controls respiratory rate, potentially causing a patient to seem ready for switching when they're still unwell. In this case, the measured respiratory rate may fall within normal ranges not because the patient has improved, but because the ventilator is artificially regulating their breathing. When using the forecasting approach, the respiratory rate can be discounted from the switch-readiness calculation very easily, or the threshold can be adjusted on a case by case basis." In my opinion, possible changes of metrics or forecasting error when one or more features are discounted compared when all features are available should be further discussed.

Minor comments

1) Page 1, lines 23-25: "IVOS depends on timely review of a changing clinical picture, but that review is easy to miss on busy wards, especially when senior input or microbiology advice is delayed". Microbiology advice may refer to different situations (e.g., availability of laboratory results, consultation by clinical microbiologist, consultation by infectious diseases specialists, structured antimicrobial stewardship feedback). Please further elaborate.

2) Page 4, lines 87-90: "The UCLH data is more easily forecastable than the MIMIC data, reflected in the lower errors for all models in each measurement. This is likely due to the fact that the MIMIC ICU cohort is made up of patients with more serious, less stable conditions." It should also be added, in my opinion, that ICU patients not only have usually more severe conditions, but also severe and unstable conditions due to non-infectious diseases that could more unpredictably impact on vital signs and increase the error.

3) Page 12, lines 303-305: "While rule-based systems have undergone prospective evaluation, these studies typically combine the intervention with education and policy changes, making it difficult to isolate the contribution of the CDSS itself." This may not be an important difference with the presence model. Rather, the need for expert evaluation of the ranking of readiness to switch proposed by the forecasting model may conversely further stress the need for education and policy changes to prompt correct evaluation and decisions made while considering the ranking (see comment 2). The key may be not to evaluate the independent contribution of the CDSS, but whether CDSS plus proper education/policy is better than proper education/policy without CDSS (or plus a rule-based system).

(Remarks on code availability)

Reviewer #2

(Remarks to the Author)
This study addresses an important and clinically relevant question—when to switch IV antibiotics to oral therapy. The authors use neural processes to forecast vital sign trajectories and infer switch readiness, and they evaluate the approach using two distinct EHR datasets to enhance generalizability. The proposed framework is particularly appealing for clinical deployment, as it supports prioritization of patients for review while preserving clinician judgment. Overall, the study is meaningful, interesting, and clearly written. However, I have several comments and questions regarding the study design and interpretation:
1) Although two different EHR datasets were used, model training and validation were conducted within each dataset separately, so true external validation is not examined. The authors should consider cross-site validation, e.g., training on UCLH and testing on MIMIC, and vice versa, which would provide stronger evidence of transportability and generalizability.
2) The study includes all inpatient admissions with antibiotic prescriptions. It would be important to clarify the underlying clinical conditions (e.g., bacteremia versus other infections) and whether it is appropriate to treat these heterogeneous cases uniformly, given that criteria for IV-to-oral switching may vary across indications.
3) Line 149: the manuscript states that "the NP model would select 2.2 more relevant patients than random selection on the UCLH data, and 3.2 on the MIMIC data." This appears to be a key finding, as also highlighted in the abstract; however, it is not entirely clear how these values were derived. A brief explanation of the calculation would improve interpretability.
4) Line 454: The manuscript stated that the overall switch-readiness probability is the product of individual probabilities across vital signs under a conditional independence assumption. This assumption requires further justification, as physiological variables are often correlated in clinical settings. The implications of this assumption should be discussed.
5) Table 3&4: the performance of the NP model is not markedly superior to that of the GBDT. Given the latter's relative simplicity, it would be helpful to include comparisons of computational cost (e.g., training time), which are relevant for real-world deployment.
6) The NP model demonstrates poorer calibration compared to the NP-tuned variant, which appears better calibrated and thus more clinically suitable. However, this distinction is not sufficiently emphasized in the Discussion. The authors should explicitly address this and consider recommending the tuned model. In addition, incorporating decision curve analysis would help evaluate clinical net benefit across plausible decision thresholds.
7) To maximise interpretability and robustness, suggest adding subgroup analyses e.g. by age, sex, ethnicity and length of stay.
8) It is unclear whether the same patient could appear in both training and test sets through separate admissions after the temporal split. This should be clarified, and if applicable, sensitivity analyses at the patient level should be considered.
9) The model predicts 'clinical stability' by vital signs measurements, which is not equal to switch-readiness. Although the authors explicitly mentioned their paper targets clinical stability in the Introduction, the manuscript repeatedly uses 'switch-readiness', which may overstate the model's clinical scope. More precise terminology would improve conceptual accuracy.

Minor comments:
1) Line 40: CDSS should be spelled out when first mentioned.
2) The Results section includes substantial methodological detail; this could be streamlined to improve readability.
3) The first paragraph of Discussion is more of Result material.
4) Figures should use color-blind friendly color palette.

(Remarks on code availability)

Reviewer #4

(Remarks to the Author)
Summary:
This paper proposes a clinical decision support system framework for IV-to-oral (IVOS) antibiotic switching. The approach decouples physiological forecasting and clinical decision-making, using neural processes (NP) to model the former, and existing clinical criteria to estimate switch-readiness probabilities and rank patients for review. The model was constructed and validated retrospectively on MIMIC-IV (ICU, USA), and UCLH (general hospital, UK). NP physiological forecasting moderately outperforms reference models including gradient boosted decision trees but yields forecasted vital signs that can be used to prioritize patients for transition from IV to Oral antibiotics.

Strengths:
- The main strength of the paper is the separation of vital sign forecasting from decision support, avoiding learning and labeling issues associated with historical prescribing actions.
- Neural processes are an appropriate approach given their ability to provide uncertainty quantification around forecasted values.
- Multi-setting validation (ICU and general ward)

Critiques:
- No comparison against a simple, interpretable rule-based IVOS alert system, which is prevalent in stewardship programs and directly relevant to the paper's claims.
- Model baselines are limited (repeat, GBDT); no comparison to strong modern irregular multivariate time series (IMTS) models (e.g., GRU-D, TFT/OmniTFT, STraTS, latent ODE/SDE); also a comparison or consideration with respect to reinforcement learning strategies
- Probabilistic forecasting is only assessed by point MAE; proper scoring rules (continuous ranked probability score, negative log-likelihood) and coverage of predictive intervals are not reported. These metrics would substantiate the quality of uncertainty used in the criteria step.
- Limited handling of patient covariates (conditioning on vital signs only, what about comorbidities, etc?) Did you assess subgroup performance (e.g., ventilated vs non-ventilated, oxygen therapy, age/sex/ethnicity)? Are there contexts where excluding certain vitals systematically improves specificity?
- The computation of switch-readiness as a product of per-time, per-variable probabilities assumes conditional independence across vitals and times; this can miscalibrate joint event probabilities.
- The MIMIC temporal split relies on reconstructed dates; although noted, this can affect precision@K and should be probed with additional leakage-controlled splits (e.g., subject-level).

Questions:
- How was the cohort constructed? Only looking at patients who were on antibiotics? If so it should be included in the data description.
- How were clinical thresholds chosen relative to published IVOS guidance (e.g., UK IVOS/NEWS2), and would you consider site-specific threshold learning or adaptive policies?
- Can these scores provide any insight as to the appropriateness of prior actions? For example, can this retrospectively identify cases in which switching was not appropriate in terms of downstream interventions or outcomes?
- How exactly are joint switch-readiness probabilities computed in deployment: by multiplying marginal probabilities at interval centers (see critique) or by sampling full trajectories? If the former, have you quantified the bias introduced by the independence assumption?

Suggestions:
- The clinical thresholds for "normal ranges" are referenced to the Supplementary; brief justification and alignment with recognized guidance (e.g., UK IVOS/NEWS2) in the main text would help.
- Beyond precision@5, how does precision/recall vary with K (review capacity)? Could you include cumulative gain or decision-curve analyses to guide site-specific deployment?

Minor comments:
In multiple tables, "bold indicates best ($p \geq 0.05$)" appears to invert the usual significance convention (likely $p \leq 0.05$).

Acronyms and References:
GRU-D: Che, Z., Purushotham, S., Cho, K. et al. Recurrent Neural Networks for Multivariate Time Series with Missing Values. Sci Rep 8, 6085 (2018). https://doi.org/10.1038/s41598-018-24271-9
TFT: Lim, B., Arik, S., Loeff, N., Pfister, T. Temporal fusion transformers for interpretable multi-horizon time series forecasting. International Journal of Forecasting 37:4 (2021). https://doi.org/10.1016/j.ijforecast.2021.03.012
TFT-Multi/OmniTFT: He, R., Chiang, J.N. Simultaneous forecasting of vital sign trajectories in the ICU. Sci Rep 15, 14996 (2025). https://doi.org/10.1038/s41598-025-99719-w
Xu, W., Dai, Y., Yang Y., Loza M., et al. OmniTFT: Omni-target forecasting for vital signs and laboratory result trajectories in multi center ICU data. arXiv (2025). https://doi.org/10.48550/arXiv.2511.19485
STraTS: Tipirnene, S., Reddy, C., Self-supervised transformer for sparse and irregularly sampled multivariate clinical time-series. ACM Transactions on Knowledge Discovery from Data (TKDD) 16:6 (2022). https://doi.org/10.1145/3516367

(Remarks on code availability)
The code repository seems appropriate and usable.

Reviewer #5

(Remarks to the Author)
I co-reviewed this manuscript with one of the reviewers who provided the listed reports. This is part of the Nature Communications initiative to facilitate training in peer review and to provide appropriate recognition for Early Career Researchers who co-review manuscripts.

(Remarks on code availability)

Version 1:

Reviewer comments:

Reviewer #1

(Remarks to the Author)
I read with interest the revised version of the retrospective study by Magnus Ross and colleagues entitled “Optimising antibiotic switching via forecasting of patent physiology”. Please find below some comments on its technical merits.

Minor comments

1) The authors should be commended for their comprehensive responses to comments and for the detailed additional analyses they provided in the revised version. In my opinion, what should be further highlighted, as a limitation, is the proof-of concept nature of the analysis and the absolute need for prospective clinical validation. Please note that this also deeply involves the additional considerations expressed by the authors in the revised version. For example, it is now (correctly) stated that “switch-readiness refers specifically to the physiological-stability component of the IVOS decision (vital signs within criteria); oral suitability and other clinical factors remain with the reviewing clinician”. The fact is that, in clinical practice, vital signs are often exactly the portion of the assessment that is very quick and easy for experts of IVOS. Consequently, it is also possible that the issue “senior review, especially around discharge planning, microbiology and stewardship input typically push prescribing towards IVOS, but these are finite resources that cannot reach every eligible patient when they are ready, delaying the switch” could not be resolved. Indeed, in clinical practice, clinicians usually check HR, RR, SpO2, SBP, and Temp every day for all patients in their ward very rapidly, also usually and easily remembering the trajectory of vital signs in the previous days (to support these statements, it should be acknowledged that I am a clinician). In those few seconds, they may have already inherently prioritized patients for IVOS and other clinically relevant tasks (and, without efforts, they also have automatically not considered RR in mechanically ventilated patients, etc.). The relevant and time-demanding work is then that of reflecting on suitability of IVOS (availability of oral options, activity of oral options against the causative agent/s, expected patients’ adherence to treatment, etc.), which is not addressed by the model. I do not mean that a model should substitute clinical judgment, but that it is possible that the proposed advantage may not be automatically present in real-world practice because it would substitute an already easy and quick task. Although I recognize that this could not be true everywhere, this is nonetheless why I’d suggest to further stress in the limitations that the performance of the model should be compared with classical approaches though appropriate prospective clinical studies (preferably RCT), to confirm whether or not a true advantage is present (e.g., what if the time to review false positives proves longer than the already quick classical approaches, in the absence of substantial advantages? On this aspect, see also the next comment) or where is present (as the efficacy of standard approaches may vary across centers)

2) The other major limitation that supports, in my opinion, the need for caution and further assessment in clinical studies is the lack of microbiological information. I recognize that now the authors state that “the limited availability of microbiology results in both datasets precluded subgroup analyses by proven infection, infection site, or antibiotic appropriateness. Because of this, it is possible that patients in the evaluation set did not have a bacterial infection at all, and it is therefore not established that forecasting performance is consistent across patients with proven infection and those without”, but this does not cancel that this major limitation remains and it might have important conceptual implications. For example, without proper subgroup analysis for confirmed infection, how one can be sure that the model is not improving switching where not needed (unconfirmed infections that could conversely benefit from a re-assessment of whether or not they truly are infections) and that this contributes relevant noise to the hypothetical benefit?

(Remarks on code availability)

Reviewer #2

(Remarks to the Author)
The authors have sufficiently addressed my previous comments, and I do not have further concerns.

(Remarks on code availability)

Reviewer #4

(Remarks to the Author)
Thank you for the thorough response. The addition of STraTS as a modern IMTS baseline, the CRPS evaluation of probabilistic forecasts, subgroup analyses, and added ablation studies and metrics satisfactorily address my critiques and questions.

(Remarks on code availability)

Reviewer #5

(Remarks to the Author)
I co-reviewed this manuscript with one of the reviewers who provided the listed reports. This is part of the Nature Communications initiative to facilitate training in peer review and to provide appropriate recognition for Early Career Researchers who co-review manuscripts.

(Remarks on code availability)

Version 2:

Reviewer comments:

Reviewer #1

(Remarks to the Author)
Thank you for your detailed responses to comments.

(Remarks on code availability)

Open Access This Peer Review File is licensed under a Creative Commons Attribution 4.0 International License, which permits use, sharing, adaptation, distribution and reproduction in any medium or format, as long as you give appropriate credit to the original author(s) and the source, provide a link to the Creative Commons license, and indicate if changes were made.
In cases where reviewers are anonymous, credit should be given to 'Anonymous Referee' and the source.
The images or other third party material in this Peer Review File are included in the article's Creative Commons license, unless indicated otherwise in a credit line to the material. If material is not included in the article's Creative Commons license and your intended use is not permitted by statutory regulation or exceeds the permitted use, you will need to obtain permission directly from the copyright holder.
To view a copy of this license, visit https://creativecommons.org/licenses/by/4.0/

Response to Reviewers

We thank the editor and all four reviewers for their careful reading of our manuscript. The reviews are thoughtful and constructive, and have prompted a number of new analyses and clarifications that we believe strengthen the work. We respond to each point in turn below. Changes in the manuscript are highlighted in green.

For each reviewer, we first reproduce their full review verbatim under a subheading, and then give our point-by-point responses. Individual comments are referenced by short codes (e.g. R1.1, R2.3, R4.c2) in the responses.

We have also made a number of small formatting changes to bring the manuscript into line with the journal guidelines, including rewording the abstract to spell out acronyms, adding bold titles and panel labels to figures, reformatting subsection headings, and reorganising the supplementary material. These purely cosmetic changes do not affect the substance of the manuscript and are not highlighted in green.

Summary of additional experiments and results

In response to the reviewers' comments we have run a number of new experiments and analyses, which we list here for convenience:

- Added STraTS [Tipirneni & Reddy, 2022] as a modern irregular multivariate time series baseline, retrained and re-evaluated on the same daily switch-readiness tasks, with results integrated into the main ranking and binary tables (see R4.c2).
- Added subgroup analyses of AUROC, average precision, and Brier score stratified by age, gender, ethnicity, and length of stay for all five models on both MIMIC and UCLH (see R2.7 and R4.c4).
- Quantified the conditional independence assumption by comparing the product-of-marginals switch-readiness probability against an autoregressive joint estimator from the same trained ConvCNP on the MIMIC test set (see R2.4 and R4.c5).
- Reported wall-clock training times for the NP, NP-tuned head, STraTS, and GBDT on the same hardware (see R2.5).
- Added a deployment-time ablation in which respiratory rate is dropped from both the label definition and the joint probability calculation on the MIMIC test set (see R1.6).
- Quantified patient-level overlap across the MIMIC temporal split and recomputed all test-set metrics on the disjoint subset of test encounters whose patient does not appear in training (see R2.8 and R4.c6).
- Added a precision@K analysis on the MIMIC test set for K in {1, 2, 3, 5, 10}, with a per-day oracle for context (see R4.s2).
- Evaluated the NP's probabilistic forecasts under the continuous ranked probability score, a strictly proper scoring rule, against the MAE of the deterministic baselines (see R4.c3).

Reviewer 1

Review

I read with interest the retrospective study by Magnus Ross and colleagues entitled "Optimising antibiotic switching via forecasting of patient physiology". Please find below some major and minor comments on its technical merits.

Major comments

1) Page 2, lines 44-48: "However, fixed rules struggle to assess whether a patient is improving rather than merely meeting thresholds at one point in time. AI enabled methods that learn from historical switching behavior face a deeper limitation: if the training signal is what clinicians did, then the best the model can do is reproduce that sub-optimal behavior. It cannot reliably improve practice against a clinical standard." I am not totally convinced by this justification. Certainly, I agree that learning from historical switching (clinicians' decisions, including suboptimal ones) is a major limitation and that this may have occurred in the previous articles cited by the authors. However, simply learning from all historical decisions is not the rule in well-designed prediction studies that lead to the development of scores for guiding decision-making in healthcare, including switch to oral antibiotics. There are proper corrections. For example, scores may

stem from studies looking at patients with switching at a given time that would be considered appropriate, vital signs at the time of switching or trend of vital signs in a time window preceding the time of switching, and subsequent relevant clinical outcomes (cure, recurrent infection, etc). This would allow to identify vital signs values associated with safe switching without relying on biased historical decisions (i.e., those suboptimal and not in line with guidelines). It may be a design issue rather than a model issue, in my opinion.

2) Page 2, lines 40-41: "The goal of a CDSS is therefore not to automate IVOS, but to surface patients for expert review and discussion with the primary team." This is in line with page 2, lines 56-58: "The system ranks patients by predicted switch-readiness, helping teams prioritise reviews while preserving clinical judgement for factors outside routine vital signs data". In my opinion, this is a correct idea and consideration. However, also the related limitations should be acknowledged, based on the study results. In this regard, there are two possible concerns, in my opinion. The first (least important) is the suboptimal recall, especially for the MIMIC dataset (ranging from 0.564 to 0.691 for the different model, including also NP and NP-tuned, as shown in Table 4). This means that at least 3 out of 10 patients who are ready to switch would be false negatives or, perhaps better, would show a low probability of readiness to switch despite they would be perfectly fit, meaning that they would not be proposed to expert clinicians for evaluation while conversely they could be readily intercepted by standard non-AI-supported approach (the cost-benefit of this possible scenario should be evaluated with dedicated future analyses). Anyway, this first concern seems to be less important clinically (although, of course, it is important from a antibiotic stewardship perspective) since the patient with true infection would continue to be treated with appropriate IV antibiotics (i.e., no expected changes in efficacy for the given treated infection). The second, more important concern regards the suboptimal precision (PPV) also in the UCLH cohort (the one in which the forecasting model seem to perform better), with a maximum of 0.667 for GBDT and 0.660 for NP-tuned. This means again that at least 3 out of 10 positives (i.e., classified as ready to switch by the model) would not be, in reality, ready to switch, and a switch to oral therapy could be deleterious in terms of efficacy. Certainly, the authors are right that this is exactly the scenario in which the expert review should intervene and that prospective studies are needed to validate this strategy (as they correctly discuss in the study limitations paragraph). However, they miss to discuss, in my opinion, also the possible impact of the forecasting model on expert decisions. Indeed, 3 out of 10 is a non-negligible frequency, and knowing this could, based on different users, either increase skepticism (high rate of not followed suggestions) or promote excessive reliance on suggestions by early adopters/clinicians unawarely favouring the technology due to baseline positive attitude toward technology, or also increasing automation bias with use (leading to perilous switches in false positives). For these reasons, not only the validation should be prospective (a term which includes also observational designs) but also randomized (implementation vs. not implementation by proper RCT design) and should take into account clinicians' attitudes toward acting or not acting according to the proposed ranking of readiness to switch (in the implementation arm).

3) In the previous comment, I used the term "true infection" (although technically "proven infection" would be more terminologically correct). Moving this consideration to the denominator of the study (study population) this can bring the attention on another crucial point, in my opinion. The study population seem to be composed by patients in the two datasets who received intravenous antibiotics. However, antibiotics may be administered also empirically, due to the suspicion of infection, in patients who are later proved not to have an infection but another disease. This is not an uncommon situation and, in these patients, any improvement in vital signs is inherently not dependent on antibiotic therapy. Of course, it can be argued that the model may works also in these patients (by suggesting, instead that they are ready to switch, that they are ready to discontinue antibiotics). However, this has to be supported by data, since it is not automatic that vital signs/trend of vital signs can similarly forecast switching to oral antibiotics in patients receiving antibiotics for a proven infection and discontinuation of antibiotics in patients receiving empirical antibiotics but later found not to have an infection. If presence/absence of a proven infection is an information available in the MIMIC and UCLH datasets I'd strongly encourage the authors to perform such a subgroup analysis. In the subgroup of patients with proven infection please also report the type and site of infection, and the etiological agent whenever available. Regarding this latter point, the availability of the etiological agent and the related susceptibility test results would also allow to create a further subgroup of patients with infection in which the administered antibiotic is appropriate

(based on expected activity given the susceptibility of the etiological agent to the administered antibiotic), although a complete assessment would also require clinicians labelling whether the PK of the antibiotic in the site of infection is appropriate and the dosage is correct. All of this is likely too much granular to be explored, nonetheless conducting subgroup analysis at least stratifying appropriateness by susceptibility test results could significantly improve the generalization potential of results, in my opinion.

4) Page 5, lines 95-97: "As the horizon increases the performance of the GBDT and NP converge, with the models providing similar performance on the 1 day horizon." This result may be problematic to externally validate the proposed model considering that it seems to work better in the UCLH cohort which includes less severe patients than in the MIMIC cohort (including more severe ICU patients), with consequent less frequent measurements. While this may not be the specific case of the UCLH cohort if a high number of availability of within 12-hour horizon measurements to properly train the model, this may not be the case for other cohorts of non-critically ill patients in which only daily measurements may be available and thus proper validation could be provided only for the 24-hour horizon (unless the validation is prospective through assessment of clinical outcomes after the switch decisions are supported by the 12-hour forecasting trained on the UCLH cohort). In other words, other retrospective studies in other non-severe cohorts to externally validate the 12-hour horizon forecasting ability may not be easy to conduct.

5) Another problem related to non-ICU cohort (i.e., those with less severe patients like the UCLH on, in which the model eventually seems to forecast better) regards the training of the model, and involves missing values. Consider page 14, lines 365-368: "A task is created on a given day only if the patient is considered to be on an IV prescription at the prediction time, there are at least 10 measurements in the preceding 48-hour look-back window, and there is at least one measurement in the 12-hour prediction window (9 am to 9 pm)"; and page 15, lines 414-416: "This choice to treat missing values as meeting criteria, rather than violating, is a modelling decision that must be clearly communicated to end users; the alternative would result in substantially more imbalanced class distributions, since missing data is common." The fact that non-severe cohorts may have more missing values may further impact on the generalization potential of results, further stressing the need for external validation in other non-severe cohorts (see previous comment).

6) Page 11, lines 239-245: "The separation between forecasting and application of criteria allows the model to be used much more flexibly in deployment. Take the example of a patient on mechanical ventilation, which controls respiratory rate, potentially causing a patient to seem ready for switching when they're still unwell. In this case, the measured respiratory rate may fall within normal ranges not because the patient has improved, but because the ventilator is artificially regulating their breathing. When using the forecasting approach, the respiratory rate can be discounted from the switch-readiness calculation very easily, or the threshold can be adjusted on a case by case basis." In my opinion, possible changes of metrics or forecasting error when one or more features are discounted compared when all features are available should be further discussed.

Minor comments

1) Page 1, lines 23-25: "IVOS depends on timely review of a changing clinical picture, but that review is easy to miss on busy wards, especially when senior input or microbiology advice is delayed". Microbiology advice may refer to different situations (e.g., availability of laboratory results, consultation by clinical microbiologist, consultation by infectious diseases specialists, structured antimicrobial stewardship feedback). Please further elaborate.

2) Page 4, lines 87-90: "The UCLH data is more easily forecastable than the MIMIC data, reflected in the lower errors for all models in each measurement. This is likely due to the fact that the MIMIC ICU cohort is made up of patients with more serious, less stable conditions." It should also be added, in my opinion, that ICU patients not only have usually more severe conditions, but also severe and unstable conditions due to non-infectious diseases that could more unpredictably impact on vital signs and increase the error.

3) Page 12, lines 303-305: "While rule-based systems have undergone prospective evaluation, these studies typically combine the intervention with education and policy changes, making it difficult to isolate the contribution of the CDSS itself." This may not be an important difference with the presence model. Rather, the need for expert evaluation of the ranking of readiness to switch proposed by the forecasting model may conversely further stress the need for education and policy changes to prompt correct evaluation

> and decisions made while considering the ranking (see comment 2). The key may be not to evaluate the independent contribution of the CDSS, but whether CDSS plus proper education/policy is better than proper education/policy without CDSS (or plus a rule-based system).

Our responses

We thank the reviewer for their careful reading of the manuscript and for the detailed and constructive comments on its technical merits. We respond to each major and minor comment in turn below.

R1.1 — On the framing of AI methods that learn from historical switching decisions.

We agree that the limitation we describe is a property of the naive set-up in which a model is trained directly on the binary "did the clinician switch?" signal, and not of supervised learning in general: well-designed studies, such as those that anchor labels to favourable downstream outcomes or to expert-curated examples of switches consistent with guidelines, can mitigate this bias. We have added a sentence to the Introduction acknowledging this, and clarifying that our contribution is best understood as *enhancing* existing rule-based IVOS criteria with probabilistic forecasting, rather than as a critique of supervised learning in general:

> *"This bias can be partly mitigated by careful study design, for example by deriving model training data from a curated cohort of switching decisions deemed appropriate by experts. Our approach instead circumvents switch labels entirely by enhancing established rule-based IVOS criteria with probabilistic forecasts of patient physiology."*

R1.2 — On the implications of imperfect precision/recall and the potential impact on clinician decision-making.

The system is intended as a *ranking* tool rather than a binary classifier: it produces a daily ranked list of patients to prioritise for review, not a "switch / do not switch" recommendation. The binary precision and recall figures in Table 4 are computed at a single F1-optimal operating point and are a secondary diagnostic; the metrics aligned with deployment (AUROC, average precision, precision@5) show that relevant patients are concentrated near the top of the ranking, with the final switch decision left to the clinician.

We nevertheless agree that the ranking framing does not fully eliminate the behavioural effects the reviewer describes, and we have added the following short sentence to the Limitations to make this explicit:

> *"The system's outputs may also shape clinician behaviour: a high false-positive rate could foster skepticism, while over-reliance could promote automation bias. Framing the output as a prioritised review list rather than a binary recommendation partly mitigates but does not eliminate these effects, which warrant explicit study in any prospective evaluation."*

We agree that a prospective, ideally randomised, evaluation is the gold standard, and consider this beyond the scope of the present, methodological contribution. To our knowledge no prior ML-based IVOS system has undergone such an evaluation.

R1.3 — On subgroup analysis by proven infection, infection site, and antibiotic appropriateness.

Unfortunately, the subgroup analyses suggested are not feasible with the data available to us: microbiological testing is performed in only a very small minority of patients receiving IV antibiotics in the MIMIC-IV or UCLH datasets.

We accept the reviewer's point that, in the absence of such an analysis, it is not established that forecasting performance is consistent across patients with proven infection and those receiving empirical antibiotics later found not to be infected. We note that the system is intended to be useful in both contexts, since it targets *physiological stability* and a forecast of sustained stability supports either a switch to oral therapy or, equivalently, safe discontinuation as the reviewer themselves notes, with the final action left to the clinician. To reflect this, we have added a sentence to the Limitations:

> *"The limited availability of microbiology results in both datasets precluded subgroup analyses by proven infection, infection site, or antibiotic appropriateness. Because of this, it is possible that patients in the evaluation set did not have a bacterial infection at all, and it is therefore not established that model performance is consistent across patients with proven infection and those without."*

R1.4 — On external validation at sites with less frequent measurements and convergence of NP and GBDT at the 1-day horizon.

We would like to clarify that the 12h forecast horizon is a property of the prediction window, not the input cadence: the NP is designed for irregularly-sampled inputs and requires only enough measurements in the 48h look-back (10 across the five vitals in our task definition), so a site with roughly daily measurements would still satisfy this. We would also stress that the 10-measurement threshold and 48h look-back are not architectural requirements, the threshold could be lowered and the look-back extended to accommodate a site with sparser sampling. The model can also be queried at any horizon, and the horizon analyses already in the manuscript (Figs 2 and 4) show that both NP and GBDT improve significantly over the repeat baseline at the 24h horizon as well. We agree that retrospective validation in additional non-severe ward cohorts at other sites would strengthen generalisability.

R1.5 — On missing values in non-severe cohorts and their impact on generalisation.

The model has been shown to perform well at two distinct levels of measurement cadence: the densely-sampled MIMIC ICU cohort and the sparser UCLH general-hospital cohort. We agree that retrospective validation in further non-severe cohorts at other sites would be valuable for establishing generalisability, but we believe the evidence from these two datasets is sufficient for the results to be meaningful. We would also note that at sufficiently high levels of missingness, no model, ours or otherwise, can be expected to produce confident forecasts, since the information needed to do so is simply not present in the data.

R1.6 — On the change in metrics or forecasting error when one or more features are discounted.

To quantify the effect of discounting a variable at deployment, we conducted an additional experiment on the MIMIC test set in which respiratory rate is dropped both from the construction of the ground-truth switch-readiness labels and from the model's switch-readiness probability calculation. This directly simulates the ventilated-patient scenario described in the manuscript, in which a clinician chooses to ignore that variable. Importantly, the underlying NP is *not* retrained: the same trained forecasting model is queried at the same target times for the same vital signs, and forecasting error per variable is therefore unchanged. What differs is only which marginal probabilities are aggregated into the joint switch-readiness probability, and which variables enter the label definition.

Excluding respiratory rate raises the prevalence of switch-ready labels from 12.2% to 27.2%, indicating that respiratory rate is the most common single reason for criteria to be violated in this cohort. Against this revised label, AUROC decreases modestly (0.882 → 0.844), while average precision (0.477 → 0.637) and precision@5 (0.311 → 0.624) both increase substantially, reflecting the higher positive-class base rate. The Brier score increases from 0.106 to 0.187 — driven mainly by the change in class balance. We have added a new section to the Supplementary Material (Supplementary Note 2, *Discounting a vital sign at deployment*) reporting these results in full and have pointed the reader to it from the relevant passage in the Discussion.

R1.minor.1 — On the meaning of "microbiology advice".

We thank the reviewer for highlighting the ambiguity. We have rephrased the sentence (Introduction, paragraph 1) to the following:

> *"IVOS depends on timely review of a changing clinical picture, but that review is easy to miss on busy wards. Senior review, especially around discharge planning, microbiology and stewardship input typically push prescribing towards IVOS, but these are finite resources that cannot reach every eligible patient when they are ready, delaying the switch."*

R1.minor.2 — On non-infectious causes of vital sign variability in the ICU cohort.

The higher forecasting error on MIMIC is indeed not only attributable to greater overall severity but also to severe non-infectious conditions that can perturb vital signs independently of the response to antibiotic therapy. We have extended the sentence in the Results to reflect this:

> *"In addition, ICU patients frequently present with severe non-infectious comorbidities which can perturb vital signs in ways that are not driven by the response to antibiotic therapy, further increasing the difficulty of forecasting."*

R1.minor.3 — On framing the role of education and policy changes alongside the CDSS.

We agree that the original sentence implicitly treated the coupling of rule-based CDSSs with education and policy changes as a weakness of the existing prospective evidence, when in practice any IVOS CDSS, including ours, would likely require at least some accompanying measures to be effective. The relevant prospective comparison is therefore CDSS plus matched education and policy support versus that support alone. We have softened the language in the Discussion accordingly:

> *"While rule-based systems have undergone prospective evaluation, these studies typically combine the intervention with education and policy changes. We note that deploying any IVOS CDSS, including ours, would likely require accompanying education and policy support. Therefore, any prospective comparison would need to assess the added value of the CDSS alone."*

Reviewer 2

Review

This study addresses an important and clinically relevant question—when to switch IV antibiotics to oral therapy. The authors use neural processes to forecast vital sign trajectories and infer switch readiness, and they evaluate the approach using two distinct EHR datasets to enhance generalizability. The proposed framework is particularly appealing for clinical deployment, as it supports prioritization of patients for review while preserving clinician judgment. Overall, the study is meaningful, interesting, and clearly written. However, I have several comments and questions regarding the study design and interpretation:

1) Although two different EHR datasets were used, model training and validation were conducted within each dataset separately, so true external validation is not examined. The authors should consider cross-site validation, e.g., training on UCLH and testing on MIMIC, and vice versa, which would provide stronger evidence of transportability and generalizability.
2) The study includes all inpatient admissions with antibiotic prescriptions. It would be important to clarify the underlying clinical conditions (e.g., bacteremia versus other infections) and whether it is appropriate to treat these heterogeneous cases uniformly, given that criteria for IV-to-oral switching may vary across indications.
3) Line 149: the manuscript states that "the NP model would select 2.2 more relevant patients than random selection on the UCLH data, and 3.2 on the MIMIC data." This appears to be a key finding, as also highlighted in the abstract; however, it is not entirely clear how these values were derived. A brief explanation of the calculation would improve interpretability.
4) Line 454: The manuscript stated that the overall switch-readiness probability is the product of individual probabilities across vital signs under a conditional independence assumption. This assumption requires further justification, as physiological variables are often correlated in clinical settings. The implications of this assumption should be discussed.
5) Table 3&4: the performance of the NP model is not markedly superior to that of the GBDT. Given the latter's relative simplicity, it would be helpful to include comparisons of computational cost (e.g., training time), which are relevant for real-world deployment.
6) The NP model demonstrates poorer calibration compared to the NP-tuned variant, which appears better calibrated and thus more clinically suitable. However, this distinction is not sufficiently emphasized in the Discussion. The authors should explicitly address this and consider recommending the tuned model. In addition, incorporating decision curve analysis would help evaluate clinical net benefit across plausible decision thresholds.
7) To maximise interpretability and robustness, suggest adding subgroup analyses e.g. by age, sex, ethnicity and length of stay.
8) It is unclear whether the same patient could appear in both training and test sets through separate admissions after the temporal split. This should be clarified, and if applicable, sensitivity analyses at the patient level should be considered.
9) The model predicts 'clinical stability' by vital signs measurements, which is not equal to switch-readiness. Although the authors explicitly mentioned their paper targets clinical stability in the Introduction, the

manuscript repeatedly uses 'switch-readiness', which may overstate the model's clinical scope. More precise terminology would improve conceptual accuracy.

Minor comments:

1) Line 40: CDSS should be spelled out when first mentioned.
2) The Results section includes substantial methodological detail; this could be streamlined to improve readability.
3) The first paragraph of Discussion is more of Result material.
4) Figures should use color-blind friendly color palette.

Our responses

We thank the reviewer for their careful reading and for noting that the study addresses an important and clinically relevant question, and that the framework is appealing for clinical deployment. We respond to each point below.

R2.1 — On cross-site (train-on-one, test-on-the-other) external validation.

We would like to clarify the intended deployment model of our system, which we believe reframes the point. Our system is not designed as a "foundation" model to be transferred between centres unchanged: the forecasting component is intended to be retrained on local data at each deploying site. A naive cross-site experiment (training on UCLH and testing on MIMIC, or vice versa) would therefore evaluate a use case we do not claim, and its result would be hard to interpret, since the two cohorts differ not only in physiology but in the criteria thresholds defining the labels, the class prevalence (12.2% switch-ready days in MIMIC vs 40.2% in UCLH), the measurement cadence, and the case-mix. Indeed, given these differences, we would expect a model transferred unchanged between these two sites to perform poorly, which is precisely why we advocate local retraining rather than direct transfer.

We would argue that meaningful evidence of generalisability is already present: the same method performs strongly on two datasets differing substantially in country, care setting (US ICU vs UK general hospital), monitoring intensity, and prescribing practice. We also note that any site with the data infrastructure to deploy a transferred model would be able to retrain the forecasting component at modest additional cost, since training is inexpensive (a single NP trains in roughly 25 minutes on one GPU; see R2.5).

R2.2 — On heterogeneity of clinical indications and indication-specific switching criteria.

We agree that switching criteria can legitimately vary across indications (e.g. bacteraemia versus other infections). Unfortunately, the clinical indication is not contemporaneously recorded against IV antibiotic prescriptions in either MIMIC-IV or UCLH, so we cannot reliably stratify the cohort by infection type or site. We have acknowledged this in the Limitations, in the text added in response to R1.3. We do note that, because forecasting and the application of clinical criteria are decoupled, indication-specific thresholds could be applied without retraining the forecasting model wherever that information is available.

While we cannot stratify by indication, in response to R2.7 we have added subgroup analyses across the patient characteristics that are reliably recorded in both datasets (age, sex, ethnicity, and length of stay), and performance is broadly consistent across these subgroups.

R2.3 — On how the 2.2× / 3.2× multipliers were derived.

The multiplier is simply the model's precision@5 divided by the precision@5 of a random ranking, which has expected value equal to the positive-class proportion (0.402 for UCLH and 0.122 for MIMIC), giving the 2.2× and 3.2× figures respectively. The manuscript already notes that random precision@5 equals the proportion of positive examples in the sentence preceding the multiplier; we have added the specific values so the calculation is clearer from the table.

R2.4 — On the conditional independence assumption used to combine per-vital probabilities.

We agree that physiological variables are typically correlated and that the product of marginals prediction assumes conditional independence across vitals and forecast times. To quantify the implications, we ran an

experiment on the MIMIC test set (22,615 encounters) comparing the standard product of marginals probability against a joint probability computed from autoregressive samples of the same trained ConvCNP, which explicitly capture correlations across vitals and forecast times. Although the ConvCNP is trained with a conditionally independent likelihood, autoregressive sampling — drawing each target point conditioned on previously sampled targets — is a well-established technique for producing coherent correlated samples from NPs trained on marginals [Bruinsma et al., 2023].

The two methods agree closely in practice: mean per-encounter switch-readiness probabilities are 0.0192 (product of marginals) versus 0.0191 (autoregressive joint), with a Spearman rank correlation of 0.86. Downstream ranking performance under the product of marginals is also at least as good, significantly so under a paired bootstrap test, on AUROC (0.882 vs 0.850), average precision (0.481 vs 0.444) and Brier score (0.106 vs 0.108). We attribute this to Monte Carlo noise in the AR estimator: the switch-ready event is rare, so a feasible number of samples gives noisy estimates that shuffle the ordering on which ranking metrics depend, whereas the product of marginals is deterministic. The product of marginals is also orders of magnitude cheaper at inference because running a prediction for N points requires N forward passes through the model vs a single forward pass for the standard case.

We have added a brief mention of this analysis to the Methods alongside the description of the probability computation, and report the full experiment in a new Supplementary section:

> *"We empirically verify that this assumption does not materially affect performance: on the MIMIC test set, probabilities computed this way agree closely (Spearman 0.86) with those from autoregressive samples [Bruinsma et al., 2023], an established technique for drawing coherent correlated samples from NPs trained on marginals, and downstream ranking metrics are at least as good (Supplementary Note 2,* Empirical assessment of the conditional independence assumption*)."*

R2.5 — On the computational cost of NP vs GBDT.

On the same hardware (a single NVIDIA A10 or A40 GPU), training a single NP on the MIMIC dataset takes approximately 25 minutes, with a further 20 minutes for the NP-tuned classification head on top, a STraTS model 1 hour, and a single GBDT 1 minute. We have added a sentence reporting the training times in the Methods alongside the description of the training hardware.

> *"In wall-clock terms, training a single NP on the MIMIC dataset took 25 minutes, with a further 20 minutes for the NP-tuned classification head on top, a STraTS model 1 hour, and a single GBDT 1 minute on this hardware."*

R2.6 — On emphasising the calibration of the NP-tuned variant and on decision curve analysis.

We agree that the calibration difference between the standard NP and the NP-tuned variant is clinically relevant and that the manuscript should be more explicit about recommending the tuned model where probability calibration matters. We have added a sentence to the Discussion, alongside the existing mention of the calibration issue, making this recommendation explicit:

> *"We therefore recommend the NP-tuned variant for any application in which the predicted probability is to be interpreted directly rather than used only as a relative ranking score."*

On decision curve analysis: we agree this is a valuable tool for evaluating clinical net benefit, but it is most informative when the model is used to make specific binary recommendations under a fixed decision threshold. Our system is instead intended to produce a daily ranked list of patients for clinician review, with the final switch decision left to the clinician; the relevant evaluation metrics for this use case are ranking-based (AUROC, AP, precision@5) rather than threshold-based. We therefore consider a full decision curve analysis beyond the scope of this work.

R2.7 — On subgroup analyses by age, sex, ethnicity, and length of stay.

We have computed subgroup-level AUROC, average precision, and Brier score for all five models (Logistic, GBDT, NP, NP-Tuned, STraTS) stratified by age band, gender, ethnicity, and length-of-stay band, on both MIMIC-tight and UCLH. The results are added to the Supplementary Material (Tables 15–22). We do not report precision@5 in this subgroup breakdown because it is a per-day metric defined over the daily ranked list, and most subgroups do not contain enough patients on a given day for the top-5 cut to be a meaningful

summary. Performance is broadly consistent across all stratifications, with the only notable deviation being patients with very short length-of-stay ($<$ 3 days) on MIMIC, who have a much higher positive prevalence (32.7% vs the cohort-wide 12.2%) reflecting the fact that patients discharged very early tend to be well at the prediction time. We have added a sentence to the Results pointing the reader to these tables:

> *"Subgroup analyses stratifying performance by age, gender, ethnicity, and length of stay on both datasets are reported in Supplementary Note 4, and show that performance is broadly consistent across subgroups."*

R2.8 — On patient-level overlap between training and test splits.

Because the test set is constructed by a temporal cut on encounter admission dates, a small minority of patients can appear in both splits via separate admissions falling either side of the cut. We have quantified this on MIMIC and recomputed all test-set metrics on the subset of test encounters whose patient does not appear in training: 8.5% of unique test patients (10.9% of test encounters) overlap with training, and all reported metrics on the disjoint subset are within the bootstrap confidence intervals of the full split for every model. We have added a sentence to the Methods making this explicit and pointed the reader to a new Supplementary section reporting the full analysis (Supplementary Note 3):

> *"Because the temporal split is encounter-based, a small minority of patients can appear in both the training and test splits via separate admissions. On MIMIC this overlap is 10.9% of test encounters. Recomputing the test-set metrics on the disjoint subset of test encounters whose patient does not appear in training leaves all reported metrics unchanged within their bootstrap confidence intervals (Supplementary Note 3)."*

This analysis is restricted to MIMIC because UCLH patient identifiers were not exported alongside the prediction outputs for data governance reasons.

R2.9 — On "switch-readiness" overstating the model's clinical scope.

We retain the "switch-readiness" label because the underlying thresholds are designed to be IVOS-specific (from consultation with the microbiology team) rather than generic stability ranges. To remove the ambiguity the reviewer identifies, we have added short clarifying clauses at the points where a reader is most likely to encounter the term in isolation, stating that "switch-readiness" in this paper refers specifically to the physiological-stability component of the IVOS decision and that oral suitability and other clinical factors remain with the reviewing clinician: the caption of Figure 1, the start of the *Switch-readiness prediction* subsection in the Results, the opening of the *Labelling switches* subsection in the Methods, and the start of the *Switch-readiness probability* subsection in the Supplementary.

R2.10 — On spelling out CDSS at first mention.

We thank the reviewer for catching this. The first occurrence in the manuscript has been expanded to "clinical decision support system (CDSS)", and the redundant expansion on the following line removed.

R2.11 — On streamlining methodological detail in the Results section.

We have kept the methodological detail in the Results section to what we judge to be the minimum needed for the results themselves to be intelligible: in particular, the definitions of the switch-ready label, the ranking-oriented metrics (precision@5, AP), and the task construction are necessary to interpret the numbers in the tables. We would prefer to retain this content in place so that the reader does not need to jump between sections to understand the figures and tables, and hope the resulting balance is acceptable.

R2.12 — On the first paragraph of the Discussion being more like Results material.

We agree that the illustrative encounter (and accompanying figure) reads more as Results material. We have moved this paragraph and the figure to the end of the Results section, and added a brief linking sentence at the start of the Discussion that refers back to the figure and introduces the broader complexities of the IVOS problem that the Discussion goes on to address.

R2.13 — On colour-blind friendly figures.

We have aimed to make the figures legible to readers with colour-blindness by using distinct symbols in each plot in addition to colour, so that series can be distinguished without relying on hue alone. If the reviewer has a specific plot in mind that they find illegible, we would be happy to revise it.

Reviewer 4

Review

Summary: This paper proposes a clinical decision support system framework for IV-to-oral (IVOS) antibiotic switching. The approach decouples physiological forecasting and clinical decision-making, using neural processes (NP) to model the former, and existing clinical criteria to estimate switch-readiness probabilities and rank patients for review. The model was constructed and validated retrospectively on MIMIC-IV (ICU, USA), and UCLH (general hospital, UK). NP physiological forecasting moderately outperforms reference models including gradient boosted decision trees but yields forecasted vital signs that can be used to prioritize patients for transition from IV to Oral antibiotics.

Strengths:

- The main strength of the paper is the separation of vital sign forecasting from decision support, avoiding learning and labeling issues associated with historical prescribing actions.
- Neural processes are an appropriate approach given their ability to provide uncertainty quantification around forecasted values.
- Multi-setting validation (ICU and general ward)

Critiques:

- No comparison against a simple, interpretable rule-based IVOS alert system, which is prevalent in stewardship programs and directly relevant to the paper's claims.
- Model baselines are limited (repeat, GBDT); no comparison to strong modern irregular multivariate time series (IMTS) models (e.g., GRU-D, TFT/OmniTFT, STraTS, latent ODE/SDE); also a comparison or consideration with respect to reinforcement learning strategies
- Probabilistic forecasting is only assessed by point MAE; proper scoring rules (continuous ranked probability score, negative log-likelihood) and coverage of predictive intervals are not reported. These metrics would substantiate the quality of uncertainty used in the criteria step.
- Limited handling of patient covariates (conditioning on vital signs only, what about comorbidities, etc?) Did you assess subgroup performance (e.g., ventilated vs non-ventilated, oxygen therapy, age/sex/ethnicity)? Are there contexts where excluding certain vitals systematically improves specificity?
- The computation of switch-readiness as a product of per-time, per-variable probabilities assumes conditional independence across vitals and times; this can miscalibrate joint event probabilities.
- The MIMIC temporal split relies on reconstructed dates; although noted, this can affect precision@K and should be probed with additional leakage-controlled splits (e.g., subject-level).

Questions:

- How was the cohort constructed? Only looking at patients who were on antibiotics? If so it should be included in the data description.
- How were clinical thresholds chosen relative to published IVOS guidance (e.g., UK IVOS/NEWS2), and would you consider site-specific threshold learning or adaptive policies?
- Can these scores provide any insight as to the appropriateness of prior actions? For example, can this retrospectively identify cases in which switching was not appropriate in terms of downstream interventions or outcomes?
- How exactly are joint switch-readiness probabilities computed in deployment: by multiplying marginal probabilities at interval centers (see critique) or by sampling full trajectories? If the former, have you quantified the bias introduced by the independence assumption?

Suggestions:

- The clinical thresholds for “normal ranges” are referenced to the Supplementary; brief justification and alignment with recognized guidance (e.g., UK IVOS/NEWS2) in the main text would help.
- Beyond precision@5, how does precision/recall vary with K (review capacity)? Could you include cumulative gain or decision-curve analyses to guide site-specific deployment?

Minor comments: In multiple tables, “bold indicates best (p   0.05)” appears to invert the usual significance convention (likely p   0.05).

Acronyms and References: GRU-D: Che, Z., Purushotham, S., Cho, K. et al. Recurrent Neural Networks for Multivariate Time Series with Missing Values. Sci Rep 8, 6085 (2018). https://doi.org/10.1038/s41598-018-24271-9

TFT: Lim, B., Arik, S., Loeff, N., Pfister, T. Temporal fusion transformers for interpretable multi-horizon time series forecasting. International Journal of Forecasting 37:4 (2021). https://doi.org/10.1016/j.ijforecast.2021.03.012

TFT-Multi/OmniTFT: He, R., Chiang, J.N. Simultaneous forecasting of vital sign trajectories in the ICU. Sci Rep 15, 14996 (2025). https://doi.org/10.1038/s41598-025-99719-w

Xu, W., Dai, Y., Yang Y., Loza M., et al. OmniTFT: Omni-target forecasting for vital signs and laboratory result trajectories in multi center ICU data. arXiv (2025). https://doi.org/10.48550/arXiv.2511.19485

STraTS: Tipirnene, S., Reddy, C., Self-supervised transformer for sparse and irregularly sampled multivariate clinical time-series. ACM Transactions on Knowledge Discovery from Data (TKDD) 16:6 (2022). https://doi.org/10.1145/3516367

Our responses

We thank the reviewer for their careful and constructive review, and for highlighting the strengths of the approach — in particular the separation of physiological forecasting from decision support, the use of neural processes for uncertainty quantification, and the multi-setting validation. We respond to each point in turn below (first critiques, then questions).

R4.c1 — On comparison against a simple, interpretable rule-based IVOS alert system.

This comparison is in fact already present: the “Repeat” row in Table 4 applies the same fixed-threshold IVOS criteria used to construct the switch-readiness label directly to the most recent measured value of each vital sign, with no forecasting, and is equivalent to a simple rule-based alert system. The Repeat / rule-based approach is necessarily absent from the ranking table (Table 3) because it produces only a binary indicator, an inability to support prioritisation that is itself a limitation of fixed-threshold alerts.

R4.c2 — On comparison against modern irregular multivariate time series (IMTS) models and reinforcement learning.

We have added STraTS [Tipirneni & Reddy, 2022] as a representative modern IMTS baseline, trained on the same daily switch-readiness tasks as the other classifiers. Results are now in Tables 3 and 4, the subgroup analyses (R2.7), the patient-overlap analysis (R2.8), and the precision@K curve (R4.s2); implementation details are in the updated Methods.

STraTS performs competitively: it achieves a small but significant improvement over the NP on AUROC on both datasets (UCLH 0.870 vs 0.858; MIMIC 0.886 vs 0.882) and on average precision on UCLH (0.805 vs 0.785), while on MIMIC the average precision of STraTS, NP and GBDT is statistically indistinguishable. On precision@5, the metric most aligned with deployment, STraTS and the NP are statistically tied on both datasets, and on the binary metrics STraTS ties with or marginally exceeds the NP. We read this as positive evidence for the paper’s central claim: the system (forecast-and-apply-criteria with a clinician-facing ranked list) is not tied to neural processes specifically and works across modern architectures. Crucially, STraTS outputs only a switch-readiness probability and cannot be queried for individual vitals at arbitrary times, so it cannot expose the per-variable forecasts central to clinician trust and to the deployment-time adjustments described in the Discussion (e.g. discounting respiratory rate for a ventilated patient). The Discussion has been updated to make this contrast explicit.

We chose STraTS as a representative of the IMTS family because it is purpose-built for sparse, irregularly sampled clinical time series and performs strongly in the benchmarks the reviewer points to; an exhaustive sweep over GRU-D, TFT/OmniTFT and latent ODE/SDE would substantially enlarge the paper without changing the methodological claim. With regards to reinforcement learning an RL formulation would require either a reward derived from downstream outcomes (inheriting the historical-decision biases we explicitly avoid, see R1.1) or curated ground-truth "correct switching" labels, neither of which is available at scale in MIMIC-IV or UCLH. We agree this is an interesting future direction but consider it out of scope here.

R4.c3 — On proper scoring rules and coverage of predictive intervals for the probabilistic forecasts.

We have evaluated the NP under the continuous ranked probability score (CRPS), a strictly proper scoring rule that integrates over the full predictive distribution. For the deterministic Repeat and GBDT baselines CRPS reduces exactly to MAE, so the values already in Table 2 provide the appropriate reference. Against this reference the NP's CRPS sits substantially below the baselines' MAEs across all five vital signs on the MIMIC test set (e.g. pulse 6.05 vs GBDT 8.92, systolic BP 8.60 vs 12.27), confirming the NP's probabilistic forecasts are meaningfully more informative than the baselines' point predictions under a proper scoring rule. Full results are in Supplementary Note 2 (*CRPS evaluation*) and a short pointer has been added to the Results.

R4.c4 — On patient covariates, subgroup performance, and excluding vitals.

Subgroup analyses by age, gender, ethnicity, and length-of-stay on both datasets have been added to the Supplementary; see R2.7 for the full set of tables. Performance is broadly consistent across subgroups. The specific case of excluding a vital sign for a relevant patient context (e.g. discounting respiratory rate for ventilated patients to improve specificity) is addressed by a new deployment-time ablation; see R1.6 for the experiment and the new Supplementary section. On the broader question of conditioning on additional static covariates (comorbidities, etc.), we deliberately kept the model inputs restricted to routinely measured vital signs to keep the system simple and broadly deployable; extending to static covariates is an interesting direction for future work.

R4.c5 — On the conditional independence assumption in switch-readiness probability.

We have quantified the empirical impact of this assumption by comparing the product-of-marginals against an autoregressive joint estimator on the MIMIC test set; the per-encounter agreement and downstream ranking metrics are reported in a new Supplementary section. See R2.4 for the full discussion.

R4.c6 — On the MIMIC reconstructed-date split and subject-level leakage controls.

We have re-evaluated all test-set discrimination metrics on the disjoint subset of MIMIC test encounters whose patient does not appear in the training split (10.9% of test encounters overlap at the patient level); all metrics on the disjoint subset are within the bootstrap confidence intervals of the full split. See R2.8 for the full analysis and the new Supplementary section.

Questions

R4.q1 — On cohort construction.

Yes, the cohort is restricted to encounters with at least one IV antibiotic prescription. This was implicit in the task-construction rule in the *Data* subsection of the Methods (a task is only created on a day when the patient is on an IV prescription, so encounters with no IV prescription contribute no tasks), but we agree it was not stated at the cohort level. We have added a sentence to the inclusion/exclusion criteria in the *Data* subsection making this explicit.

R4.q2 — On choice of clinical thresholds and site-specific / adaptive policies.

The thresholds were developed in consultation with the microbiology team at our institution, using the National Early Warning Score 2 (NEWS-2) thresholds as a starting reference (the score 1 level for each variable), and held fixed across both datasets. The MIMIC-loose sensitivity analysis already in the manuscript (positive class rises from 12.2% to 50.0%) shows the relative ordering of the top models is essentially unchanged under substantially different thresholds (Supplementary Note 2, *Criteria threshold sensitivity*). Because the system

decouples forecasting from criteria, a deploying site can substitute its own thresholds without retraining the forecasting model; principled adaptive or learned thresholds are an interesting direction for future work.

R4.q3 — On using the scores to retrospectively assess the appropriateness of prior switching decisions.

In principle the ranked switch-readiness probabilities could be used to flag historical cases in which the model would have indicated readiness well before an actual switch (or where no switch occurred despite sustained readiness), and similar retrospective audits of antibiotic prescribing have been performed in prior work [Dutey-Magni et al., 2021]. We did not pursue this analysis here because it requires switch labels and these are not consistently captured in either dataset. We agree this is a valuable direction for future work.

R4.q4 — On the computation of joint switch-readiness probabilities and the bias of the independence assumption.

The product-of-marginals computation is described in the *Neural processes* subsection of the Methods and the *Further neural process details* subsection of the Supplementary Methods, and the empirical bias of the independence assumption is addressed in responses R2.4 / R4.c5.

Suggestions

R4.s1 — On brief justification of clinical thresholds and alignment with recognized guidance.

We have added a short clause to the *Labelling switches* subsection of the Methods clarifying that the thresholds were anchored to the NEWS-2 score-1 boundaries, with the final levels set in consultation with the microbiology team at our institution.

R4.s2 — On precision/recall as a function of K and decision-curve / cumulative-gain analyses.

We have added a precision@K analysis for $K \in \{1, 2, 3, 5, 10\}$ on the MIMIC test set, with a per-day oracle for context (Supplementary Note 2, *Precision@K across review-capacity values*); a pointer has been added to the Results. The relative ordering of models is essentially stable across K. On decision-curve analysis: as noted in R2.6, our system is intended to produce a ranked list of patients for review rather than a binary recommendation under a fixed threshold, so the relevant evaluation metrics are ranking-based; we therefore consider a full decision-curve analysis beyond the scope of this work.

Minor comments

R4.m1 — On the significance convention in table captions ("bold indicates best (p $\geq$ 0.05)" appearing inverted).

We thank the reviewer for flagging the ambiguity. The intended meaning is that bold marks the top-performing model on each metric *together with any models not significantly worse than it* under a paired bootstrap test, so the $p \geq 0.05$ threshold is in fact the correct direction (it identifies models statistically indistinguishable from the best). The full procedure, including the Bonferroni correction for multiple comparisons, is described in the Methods (*Statistical significance testing*). We have revised the captions of Tables 2, 3 and 4 in the main text, and Supplementary Tables 8 and 9, to spell this out and to point the reader to the significance-testing section, so the convention is no longer ambiguous.

Response to Reviewers (Second Round)

We thank the editor and reviewers for the further consideration of our manuscript "Optimising antibiotic switching via forecasting of patient physiology". We are pleased that Reviewers 2 and 4 have no further concerns. Reviewer 1 raised two additional points, both clinical in nature, which we address below. As before, we reproduce the review verbatim and then give point-by-point responses. In the manuscript we highlight changes from this revision in blue, with changes from the previous revisions remaining in green.

Reviewer 1

Review

I read with interest the revised version of the retrospective study by Magnus Ross and colleagues entitled "Optimising antibiotic switching via forecasting of patent physiology". Please find below some comments on its technical merits.

Minor comments

1) The authors should be commended for their comprehensive responses to comments and for the detailed additional analyses they provided in the revised version. In my opinion, what should be further highlighted, as a limitation, is the proof-of concept nature of the analysis and the absolute need for prospective clinical validation. Please note that this also deeply involves the additional considerations expressed by the authors in the revised version. For example, it is now (correctly) stated that "switch-readiness refers specifically to the physiological-stability component of the IVOS decision (vital signs within criteria); oral suitability and other clinical factors remain with the reviewing clinician". The fact is that, in clinical practice, vital signs are often exactly the portion of the assessment that is very quick and easy for experts of IVOS. Consequently, it is also possible that the issue "senior review, especially around discharge planning, microbiology and stewardship input typically push prescribing towards IVOS, but these are finite resources that cannot reach every eligible patient when they are ready, delaying the switch" could not be resolved. Indeed, in clinical practice, clinicians usually check HR, RR, SpO2, SBP, and Temp every day for all patients in their ward very rapidly, also usually and easily remembering the trajectory of vital signs in the previous days (to support these statements, it should be acknowledged that I am a clinician). In those few seconds, they may have already inherently prioritized patients for IVOS and other clinically relevant tasks (and, without efforts, they also have automatically not considered RR in mechanically ventilated patients, etc.). The relevant and time-demanding work is then that of reflecting on suitability of IVOS (availability of oral options, activity of oral options against the causative agent/s, expected patients' adherence to treatment, etc.), which is not addressed by the model. I do not mean that a model should substitute clinical judgment, but that it is possible that the proposed advantage may not be automatically present in real-world practice because it would substitute an already easy and quick task. Although I recognize that this could not be true everywhere, this is nonetheless why I'd suggest to further stress in the limitations that the performance of the model should be compared with classical approaches though appropriate prospective clinical studies (preferably RCT), to confirm whether or not a true advantage is present (e.g., what if the time to review false positives proves longer than the already quick classical approaches, in the absence of substantial advantages? On this aspect, see also the next comment) or where is present (as the efficacy of standard approaches may vary across centers)

2) The other major limitation that supports, in my opinion, the need for caution and further assessment in clinical studies is the lack of microbiological information. I recognize that now the authors state that "the limited availability of microbiology results in both datasets precluded subgroup analyses by proven infection, infection site, or antibiotic appropriateness. Because of this, it is possible that patients in the evaluation set did not have a bacterial infection at all, and it is therefore not established that forecasting performance is consistent across patients with proven infection and those without", but this does not cancel that this major limitation remains and it might have important conceptual implications. For example, without proper subgroup analysis for confirmed infection, how one can be sure that the model is not improving switching where not needed (unconfirmed infections that could conversely benefit from a

re-assessment of whether or not they truly are infections) and that this contributes relevant noise to the hypothetical benefit?

Our responses

We thank the reviewer for these two further comments. We address them below.

R1.1 — On vital-sign assessment being the quick part of the IVOS decision, and the consequent need for prospective evaluation.

We agree with the reviewer's clinical observation that, for a single patient, checking vital signs against criteria is quick, and that the more time-consuming work of judging oral suitability (tolerance, availability of oral alternatives, likely adherence etc.) is not addressed by the model and remains entirely with the clinician. We would make two points in response.

First, the model is doing non-trivial forecasting rather than re-presenting the current values: the "Repeat" baseline applies the criteria directly to the latest measurement (essentially the snapshot a clinician reads at the bedside) and is significantly outperformed by the forecasting models on both datasets, indicating that there is information in the vital-sign trajectory beyond a point-in-time check.

Second, our claim is not that the system speeds up the per-patient assessment necessarily but that it provides a prioritisation function. While identifying an improving patient is easy for a clinician reviewing a single case, reliably performing that assessment across every eligible inpatient each day is not. This is reflected in the persistent under-switching that motivates the work (e.g. 19% of patients in England remaining on IV antibiotics despite meeting the criteria). The system is designed for the setting in which there is not enough capacity to review every patient, allowing limited review effort to be directed at those most likely to be ready.

We fully agree with the reviewer that whether this prioritisation confers a real-world advantage can only be established by prospective, ideally randomised, comparison against standard practice. We have made this more explicit in the Discussion:

> *"Such an evaluation would also need to establish the value of the prioritisation itself: because assessing a single patient against the criteria is already quick, any benefit must instead come from directing finite review capacity across every eligible inpatient, which retrospective metrics cannot demonstrate."*

R1.2 — On the lack of microbiological confirmation

We accept that, without microbiological confirmation, we cannot be certain every patient in the cohort had a bacterial infection. We would note, however, that in most hospitals the majority of patients receiving IV antibiotics never have a microbiologically confirmed infection, while IVOS decisions must still be made for them daily; restricting to confirmed infection would exclude much of the population in which the decision is actually faced. Prior ML work on IVOS likewise does not restrict to microbiologically confirmed cases [Bolton et al., 2024].

More importantly, we would suggest that surfacing such patients prompts useful clinical review rather than introducing noise. The system flags physiological stability and leaves the resulting action to the clinician; for a patient who may not have a bacterial infection, that same review is a natural opportunity to reassess whether antimicrobials are needed at all. That being said, we have not evaluated the system as a discontinuation tool, and our results concern ranking for switch-readiness only.

We have added the following to the discussion to reflect these points.

> *"We note, however, that most patients receiving IV antibiotics never have a microbiologically confirmed infection, while IVOS decisions must still be made for them. Because the system flags physiological stability and leaves the resulting action to the clinician, surfacing such patients still prompts a review that may identify either a switch or, where appropriate, a reassessment of the need for antimicrobials."*

Reviewers 2 and 4

We thank Reviewers 2 and 4 for confirming that their previous comments have been satisfactorily addressed. We have no further changes to make in response to these reports.

Response to Reviewers

We thank the editor and all four reviewers for their careful reading of our manuscript. The reviews are thoughtful and constructive, and have prompted a number of new analyses and clarifications that we believe strengthen the work. We respond to each point in turn below. Changes in the manuscript are highlighted in green.

For each reviewer, we first reproduce their full review verbatim under a subheading, and then give our point-by-point responses. Individual comments are referenced by short codes (e.g. R1.1, R2.3, R4.c2) in the responses.

We have also made a number of small formatting changes to bring the manuscript into line with the journal guidelines, including rewording the abstract to spell out acronyms, adding bold titles and panel labels to figures, reformatting subsection headings, and reorganising the supplementary material. These purely cosmetic changes do not affect the substance of the manuscript and are not highlighted in green.

Summary of additional experiments and results

In response to the reviewers' comments we have run a number of new experiments and analyses, which we list here for convenience:

- Added STraTS [Tipirneni & Reddy, 2022] as a modern irregular multivariate time series baseline, retrained and re-evaluated on the same daily switch-readiness tasks, with results integrated into the main ranking and binary tables (see R4.c2).
- Added subgroup analyses of AUROC, average precision, and Brier score stratified by age, gender, ethnicity, and length of stay for all five models on both MIMIC and UCLH (see R2.7 and R4.c4).
- Quantified the conditional independence assumption by comparing the product-of-marginals switch-readiness probability against an autoregressive joint estimator from the same trained ConvCNP on the MIMIC test set (see R2.4 and R4.c5).
- Reported wall-clock training times for the NP, NP-tuned head, STraTS, and GBDT on the same hardware (see R2.5).
- Added a deployment-time ablation in which respiratory rate is dropped from both the label definition and the joint probability calculation on the MIMIC test set (see R1.6).
- Quantified patient-level overlap across the MIMIC temporal split and recomputed all test-set metrics on the disjoint subset of test encounters whose patient does not appear in training (see R2.8 and R4.c6).
- Added a precision@K analysis on the MIMIC test set for K in {1, 2, 3, 5, 10}, with a per-day oracle for context (see R4.s2).
- Evaluated the NP's probabilistic forecasts under the continuous ranked probability score, a strictly proper scoring rule, against the MAE of the deterministic baselines (see R4.c3).

Reviewer 1

Review

I read with interest the retrospective study by Magnus Ross and colleagues entitled "Optimising antibiotic switching via forecasting of patient physiology". Please find below some major and minor comments on its technical merits.

Major comments

1) Page 2, lines 44-48: "However, fixed rules struggle to assess whether a patient is improving rather than merely meeting thresholds at one point in time. AI enabled methods that learn from historical switching behavior face a deeper limitation: if the training signal is what clinicians did, then the best the model can do is reproduce that sub-optimal behavior. It cannot reliably improve practice against a clinical standard." I am not totally convinced by this justification. Certainly, I agree that learning from historical switching (clinicians' decisions, including suboptimal ones) is a major limitation and that this may have occurred in the previous articles cited by the authors. However, simply learning from all historical decisions is not the rule in well-designed prediction studies that lead to the development of scores for guiding decision-making in healthcare, including switch to oral antibiotics. There are proper corrections. For example, scores may

stem from studies looking at patients with switching at a given time that would be considered appropriate, vital signs at the time of switching or trend of vital signs in a time window preceding the time of switching, and subsequent relevant clinical outcomes (cure, recurrent infection, etc). This would allow to identify vital signs values associated with safe switching without relying on biased historical decisions (i.e., those suboptimal and not in line with guidelines). It may be a design issue rather than a model issue, in my opinion.

2) Page 2, lines 40-41: “The goal of a CDSS is therefore not to automate IVOS, but to surface patients for expert review and discussion with the primary team.” This is in line with page 2, lines 56-58: “The system ranks patients by predicted switch-readiness, helping teams prioritise reviews while preserving clinical judgement for factors outside routine vital signs data”. In my opinion, this is a correct idea and consideration. However, also the related limitations should be acknowledged, based on the study results. In this regard, there are two possible concerns, in my opinion. The first (least important) is the suboptimal recall, especially for the MIMIC dataset (ranging from 0.564 to 0.691 for the different model, including also NP and NP-tuned, as shown in Table 4). This means that at least 3 out of 10 patients who are ready to switch would be false negatives or, perhaps better, would show a low probability of readiness to switch despite they would be perfectly fit, meaning that they would not be proposed to expert clinicians for evaluation while conversely they could be readily intercepted by standard non-AI-supported approach (the cost-benefit of this possible scenario should be evaluated with dedicated future analyses). Anyway, this first concern seems to be less important clinically (although, of course, it is important from a antibiotic stewardship perspective) since the patient with true infection would continue to be treated with appropriate IV antibiotics (i.e., no expected changes in efficacy for the given treated infection). The second, more important concern regards the suboptimal precision (PPV) also in the UCLH cohort (the one in which the forecasting model seem to perform better), with a maximum of 0.667 for GBDT and 0.660 for NP-tuned. This means again that at least 3 out of 10 positives (i.e., classified as ready to switch by the model) would not be, in reality, ready to switch, and a switch to oral therapy could be deleterious in terms of efficacy. Certainly, the authors are right that this is exactly the scenario in which the expert review should intervene and that prospective studies are needed to validate this strategy (as they correctly discuss in the study limitations paragraph). However, they miss to discuss, in my opinion, also the possible impact of the forecasting model on expert decisions. Indeed, 3 out of 10 is a non-negligible frequency, and knowing this could, based on different users, either increase skepticism (high rate of not followed suggestions) or promote excessive reliance on suggestions by early adopters/clinicians unawarely favouring the technology due to baseline positive attitude toward technology, or also increasing automation bias with use (leading to perilous switches in false positives). For these reasons, not only the validation should be prospective (a term which includes also observational designs) but also randomized (implementation vs. not implementation by proper RCT design) and should take into account clinicians' attitudes toward acting or not acting according to the proposed ranking of readiness to switch (in the implementation arm).

3) In the previous comment, I used the term “true infection” (although technically “proven infection” would be more terminologically correct). Moving this consideration to the denominator of the study (study population) this can bring the attention on another crucial point, in my opinion. The study population seem to be composed by patients in the two datasets who received intravenous antibiotics. However, antibiotics may be administered also empirically, due to the suspicion of infection, in patients who are later proved not to have an infection but another disease. This is not an uncommon situation and, in these patients, any improvement in vital signs is inherently not dependent on antibiotic therapy. Of course, it can be argued that the model may works also in these patients (by suggesting, instead that they are ready to switch, that they are ready to discontinue antibiotics). However, this has to be supported by data, since it is not automatic that vital signs/trend of vital signs can similarly forecast switching to oral antibiotics in patients receiving antibiotics for a proven infection and discontinuation of antibiotics in patients receiving empirical antibiotics but later found not to have an infection. If presence/absence of a proven infection is an information available in the MIMIC and UCLH datasets I'd strongly encourage the authors to perform such a subgroup analysis. In the subgroup of patients with proven infection please also report the type and site of infection, and the etiological agent whenever available. Regarding this latter point, the availability of the etiological agent and the related susceptibility test results would also allow to create a further subgroup of patients with infection in which the administered antibiotic is appropriate

(based on expected activity given the susceptibility of the etiological agent to the administered antibiotic), although a complete assessment would also require clinicians labelling whether the PK of the antibiotic in the site of infection is appropriate and the dosage is correct. All of this is likely too much granular to be explored, nonetheless conducting subgroup analysis at least stratifying appropriateness by susceptibility test results could significantly improve the generalization potential of results, in my opinion.

4) Page 5, lines 95-97: "As the horizon increases the performance of the GBDT and NP converge, with the models providing similar performance on the 1 day horizon." This result may be problematic to externally validate the proposed model considering that it seems to work better in the UCLH cohort which includes less severe patients than in the MIMIC cohort (including more severe ICU patients), with consequent less frequent measurements. While this may not be the specific case of the UCLH cohort if a high number of availability of within 12-hour horizon measurements to properly train the model, this may not be the case for other cohorts of non-critically ill patients in which only daily measurements may be available and thus proper validation could be provided only for the 24-hour horizon (unless the validation is prospective through assessment of clinical outcomes after the switch decisions are supported by the 12-hour forecasting trained on the UCLH cohort). In other words, other retrospective studies in other non-severe cohorts to externally validate the 12-hour horizon forecasting ability may not be easy to conduct.

5) Another problem related to non-ICU cohort (i.e., those with less severe patients like the UCLH on, in which the model eventually seems to forecast better) regards the training of the model, and involves missing values. Consider page 14, lines 365-368: "A task is created on a given day only if the patient is considered to be on an IV prescription at the prediction time, there are at least 10 measurements in the preceding 48-hour look-back window, and there is at least one measurement in the 12-hour prediction window (9 am to 9 pm)"; and page 15, lines 414-416: "This choice to treat missing values as meeting criteria, rather than violating, is a modelling decision that must be clearly communicated to end users; the alternative would result in substantially more imbalanced class distributions, since missing data is common." The fact that non-severe cohorts may have more missing values may further impact on the generalization potential of results, further stressing the need for external validation in other non-severe cohorts (see previous comment).

6) Page 11, lines 239-245: "The separation between forecasting and application of criteria allows the model to be used much more flexibly in deployment. Take the example of a patient on mechanical ventilation, which controls respiratory rate, potentially causing a patient to seem ready for switching when they're still unwell. In this case, the measured respiratory rate may fall within normal ranges not because the patient has improved, but because the ventilator is artificially regulating their breathing. When using the forecasting approach, the respiratory rate can be discounted from the switch-readiness calculation very easily, or the threshold can be adjusted on a case by case basis." In my opinion, possible changes of metrics or forecasting error when one or more features are discounted compared when all features are available should be further discussed.

Minor comments

1) Page 1, lines 23-25: "IVOS depends on timely review of a changing clinical picture, but that review is easy to miss on busy wards, especially when senior input or microbiology advice is delayed". Microbiology advice may refer to different situations (e.g., availability of laboratory results, consultation by clinical microbiologist, consultation by infectious diseases specialists, structured antimicrobial stewardship feedback). Please further elaborate.

2) Page 4, lines 87-90: "The UCLH data is more easily forecastable than the MIMIC data, reflected in the lower errors for all models in each measurement. This is likely due to the fact that the MIMIC ICU cohort is made up of patients with more serious, less stable conditions." It should also be added, in my opinion, that ICU patients not only have usually more severe conditions, but also severe and unstable conditions due to non-infectious diseases that could more unpredictably impact on vital signs and increase the error.

3) Page 12, lines 303-305: "While rule-based systems have undergone prospective evaluation, these studies typically combine the intervention with education and policy changes, making it difficult to isolate the contribution of the CDSS itself." This may not be an important difference with the presence model. Rather, the need for expert evaluation of the ranking of readiness to switch proposed by the forecasting model may conversely further stress the need for education and policy changes to prompt correct evaluation

> and decisions made while considering the ranking (see comment 2). The key may be not to evaluate the independent contribution of the CDSS, but whether CDSS plus proper education/policy is better than proper education/policy without CDSS (or plus a rule-based system).

Our responses

We thank the reviewer for their careful reading of the manuscript and for the detailed and constructive comments on its technical merits. We respond to each major and minor comment in turn below.

R1.1 — On the framing of AI methods that learn from historical switching decisions.

We agree that the limitation we describe is a property of the naive set-up in which a model is trained directly on the binary "did the clinician switch?" signal, and not of supervised learning in general: well-designed studies, such as those that anchor labels to favourable downstream outcomes or to expert-curated examples of switches consistent with guidelines, can mitigate this bias. We have added a sentence to the Introduction acknowledging this, and clarifying that our contribution is best understood as *enhancing* existing rule-based IVOS criteria with probabilistic forecasting, rather than as a critique of supervised learning in general:

> *"This bias can be partly mitigated by careful study design, for example by deriving model training data from a curated cohort of switching decisions deemed appropriate by experts. Our approach instead circumvents switch labels entirely by enhancing established rule-based IVOS criteria with probabilistic forecasts of patient physiology."*

R1.2 — On the implications of imperfect precision/recall and the potential impact on clinician decision-making.

The system is intended as a *ranking* tool rather than a binary classifier: it produces a daily ranked list of patients to prioritise for review, not a "switch / do not switch" recommendation. The binary precision and recall figures in Table 4 are computed at a single F1-optimal operating point and are a secondary diagnostic; the metrics aligned with deployment (AUROC, average precision, precision@5) show that relevant patients are concentrated near the top of the ranking, with the final switch decision left to the clinician.

We nevertheless agree that the ranking framing does not fully eliminate the behavioural effects the reviewer describes, and we have added the following short sentence to the Limitations to make this explicit:

> *"The system's outputs may also shape clinician behaviour: a high false-positive rate could foster skepticism, while over-reliance could promote automation bias. Framing the output as a prioritised review list rather than a binary recommendation partly mitigates but does not eliminate these effects, which warrant explicit study in any prospective evaluation."*

We agree that a prospective, ideally randomised, evaluation is the gold standard, and consider this beyond the scope of the present, methodological contribution. To our knowledge no prior ML-based IVOS system has undergone such an evaluation.

R1.3 — On subgroup analysis by proven infection, infection site, and antibiotic appropriateness.

Unfortunately, the subgroup analyses suggested are not feasible with the data available to us: microbiological testing is performed in only a very small minority of patients receiving IV antibiotics in the MIMIC-IV or UCLH datasets.

We accept the reviewer's point that, in the absence of such an analysis, it is not established that forecasting performance is consistent across patients with proven infection and those receiving empirical antibiotics later found not to be infected. We note that the system is intended to be useful in both contexts, since it targets *physiological stability* and a forecast of sustained stability supports either a switch to oral therapy or, equivalently, safe discontinuation as the reviewer themselves notes, with the final action left to the clinician. To reflect this, we have added a sentence to the Limitations:

> *"The limited availability of microbiology results in both datasets precluded subgroup analyses by proven infection, infection site, or antibiotic appropriateness. Because of this, it is possible that patients in the evaluation set did not have a bacterial infection at all, and it is therefore not established that model performance is consistent across patients with proven infection and those without."*

R1.4 — On external validation at sites with less frequent measurements and convergence of NP and GBDT at the 1-day horizon.

We would like to clarify that the 12h forecast horizon is a property of the prediction window, not the input cadence: the NP is designed for irregularly-sampled inputs and requires only enough measurements in the 48h look-back (10 across the five vitals in our task definition), so a site with roughly daily measurements would still satisfy this. We would also stress that the 10-measurement threshold and 48h look-back are not architectural requirements, the threshold could be lowered and the look-back extended to accommodate a site with sparser sampling. The model can also be queried at any horizon, and the horizon analyses already in the manuscript (Figs 2 and 4) show that both NP and GBDT improve significantly over the repeat baseline at the 24h horizon as well. We agree that retrospective validation in additional non-severe ward cohorts at other sites would strengthen generalisability.

R1.5 — On missing values in non-severe cohorts and their impact on generalisation.

The model has been shown to perform well at two distinct levels of measurement cadence: the densely-sampled MIMIC ICU cohort and the sparser UCLH general-hospital cohort. We agree that retrospective validation in further non-severe cohorts at other sites would be valuable for establishing generalisability, but we believe the evidence from these two datasets is sufficient for the results to be meaningful. We would also note that at sufficiently high levels of missingness, no model, ours or otherwise, can be expected to produce confident forecasts, since the information needed to do so is simply not present in the data.

R1.6 — On the change in metrics or forecasting error when one or more features are discounted.

To quantify the effect of discounting a variable at deployment, we conducted an additional experiment on the MIMIC test set in which respiratory rate is dropped both from the construction of the ground-truth switch-readiness labels and from the model's switch-readiness probability calculation. This directly simulates the ventilated-patient scenario described in the manuscript, in which a clinician chooses to ignore that variable. Importantly, the underlying NP is *not* retrained: the same trained forecasting model is queried at the same target times for the same vital signs, and forecasting error per variable is therefore unchanged. What differs is only which marginal probabilities are aggregated into the joint switch-readiness probability, and which variables enter the label definition.

Excluding respiratory rate raises the prevalence of switch-ready labels from 12.2% to 27.2%, indicating that respiratory rate is the most common single reason for criteria to be violated in this cohort. Against this revised label, AUROC decreases modestly (0.882 → 0.844), while average precision (0.477 → 0.637) and precision@5 (0.311 → 0.624) both increase substantially, reflecting the higher positive-class base rate. The Brier score increases from 0.106 to 0.187 — driven mainly by the change in class balance. We have added a new section to the Supplementary Material (Supplementary Note 2, *Discounting a vital sign at deployment*) reporting these results in full and have pointed the reader to it from the relevant passage in the Discussion.

R1.minor.1 — On the meaning of "microbiology advice".

We thank the reviewer for highlighting the ambiguity. We have rephrased the sentence (Introduction, paragraph 1) to the following:

> *"IVOS depends on timely review of a changing clinical picture, but that review is easy to miss on busy wards. Senior review, especially around discharge planning, microbiology and stewardship input typically push prescribing towards IVOS, but these are finite resources that cannot reach every eligible patient when they are ready, delaying the switch."*

R1.minor.2 — On non-infectious causes of vital sign variability in the ICU cohort.

The higher forecasting error on MIMIC is indeed not only attributable to greater overall severity but also to severe non-infectious conditions that can perturb vital signs independently of the response to antibiotic therapy. We have extended the sentence in the Results to reflect this:

> *"In addition, ICU patients frequently present with severe non-infectious comorbidities which can perturb vital signs in ways that are not driven by the response to antibiotic therapy, further increasing the difficulty of forecasting."*

R1.minor.3 — On framing the role of education and policy changes alongside the CDSS.

We agree that the original sentence implicitly treated the coupling of rule-based CDSSs with education and policy changes as a weakness of the existing prospective evidence, when in practice any IVOS CDSS, including ours, would likely require at least some accompanying measures to be effective. The relevant prospective comparison is therefore CDSS plus matched education and policy support versus that support alone. We have softened the language in the Discussion accordingly:

> *"While rule-based systems have undergone prospective evaluation, these studies typically combine the intervention with education and policy changes. We note that deploying any IVOS CDSS, including ours, would likely require accompanying education and policy support. Therefore, any prospective comparison would need to assess the added value of the CDSS alone."*

Reviewer 2

Review

This study addresses an important and clinically relevant question—when to switch IV antibiotics to oral therapy. The authors use neural processes to forecast vital sign trajectories and infer switch readiness, and they evaluate the approach using two distinct EHR datasets to enhance generalizability. The proposed framework is particularly appealing for clinical deployment, as it supports prioritization of patients for review while preserving clinician judgment. Overall, the study is meaningful, interesting, and clearly written. However, I have several comments and questions regarding the study design and interpretation:

1) Although two different EHR datasets were used, model training and validation were conducted within each dataset separately, so true external validation is not examined. The authors should consider cross-site validation, e.g., training on UCLH and testing on MIMIC, and vice versa, which would provide stronger evidence of transportability and generalizability.
2) The study includes all inpatient admissions with antibiotic prescriptions. It would be important to clarify the underlying clinical conditions (e.g., bacteremia versus other infections) and whether it is appropriate to treat these heterogeneous cases uniformly, given that criteria for IV-to-oral switching may vary across indications.
3) Line 149: the manuscript states that "the NP model would select 2.2 more relevant patients than random selection on the UCLH data, and 3.2 on the MIMIC data." This appears to be a key finding, as also highlighted in the abstract; however, it is not entirely clear how these values were derived. A brief explanation of the calculation would improve interpretability.
4) Line 454: The manuscript stated that the overall switch-readiness probability is the product of individual probabilities across vital signs under a conditional independence assumption. This assumption requires further justification, as physiological variables are often correlated in clinical settings. The implications of this assumption should be discussed.
5) Table 3&4: the performance of the NP model is not markedly superior to that of the GBDT. Given the latter's relative simplicity, it would be helpful to include comparisons of computational cost (e.g., training time), which are relevant for real-world deployment.
6) The NP model demonstrates poorer calibration compared to the NP-tuned variant, which appears better calibrated and thus more clinically suitable. However, this distinction is not sufficiently emphasized in the Discussion. The authors should explicitly address this and consider recommending the tuned model. In addition, incorporating decision curve analysis would help evaluate clinical net benefit across plausible decision thresholds.
7) To maximise interpretability and robustness, suggest adding subgroup analyses e.g. by age, sex, ethnicity and length of stay.
8) It is unclear whether the same patient could appear in both training and test sets through separate admissions after the temporal split. This should be clarified, and if applicable, sensitivity analyses at the patient level should be considered.
9) The model predicts 'clinical stability' by vital signs measurements, which is not equal to switch-readiness. Although the authors explicitly mentioned their paper targets clinical stability in the Introduction, the

manuscript repeatedly uses 'switch-readiness', which may overstate the model's clinical scope. More precise terminology would improve conceptual accuracy.

Minor comments:

1) Line 40: CDSS should be spelled out when first mentioned.
2) The Results section includes substantial methodological detail; this could be streamlined to improve readability.
3) The first paragraph of Discussion is more of Result material.
4) Figures should use color-blind friendly color palette.

Our responses

We thank the reviewer for their careful reading and for noting that the study addresses an important and clinically relevant question, and that the framework is appealing for clinical deployment. We respond to each point below.

R2.1 — On cross-site (train-on-one, test-on-the-other) external validation.

We would like to clarify the intended deployment model of our system, which we believe reframes the point. Our system is not designed as a "foundation" model to be transferred between centres unchanged: the forecasting component is intended to be retrained on local data at each deploying site. A naive cross-site experiment (training on UCLH and testing on MIMIC, or vice versa) would therefore evaluate a use case we do not claim, and its result would be hard to interpret, since the two cohorts differ not only in physiology but in the criteria thresholds defining the labels, the class prevalence (12.2% switch-ready days in MIMIC vs 40.2% in UCLH), the measurement cadence, and the case-mix. Indeed, given these differences, we would expect a model transferred unchanged between these two sites to perform poorly, which is precisely why we advocate local retraining rather than direct transfer.

We would argue that meaningful evidence of generalisability is already present: the same method performs strongly on two datasets differing substantially in country, care setting (US ICU vs UK general hospital), monitoring intensity, and prescribing practice. We also note that any site with the data infrastructure to deploy a transferred model would be able to retrain the forecasting component at modest additional cost, since training is inexpensive (a single NP trains in roughly 25 minutes on one GPU; see R2.5).

R2.2 — On heterogeneity of clinical indications and indication-specific switching criteria.

We agree that switching criteria can legitimately vary across indications (e.g. bacteraemia versus other infections). Unfortunately, the clinical indication is not contemporaneously recorded against IV antibiotic prescriptions in either MIMIC-IV or UCLH, so we cannot reliably stratify the cohort by infection type or site. We have acknowledged this in the Limitations, in the text added in response to R1.3. We do note that, because forecasting and the application of clinical criteria are decoupled, indication-specific thresholds could be applied without retraining the forecasting model wherever that information is available.

While we cannot stratify by indication, in response to R2.7 we have added subgroup analyses across the patient characteristics that are reliably recorded in both datasets (age, sex, ethnicity, and length of stay), and performance is broadly consistent across these subgroups.

R2.3 — On how the 2.2× / 3.2× multipliers were derived.

The multiplier is simply the model's precision@5 divided by the precision@5 of a random ranking, which has expected value equal to the positive-class proportion (0.402 for UCLH and 0.122 for MIMIC), giving the 2.2× and 3.2× figures respectively. The manuscript already notes that random precision@5 equals the proportion of positive examples in the sentence preceding the multiplier; we have added the specific values so the calculation is clearer from the table.

R2.4 — On the conditional independence assumption used to combine per-vital probabilities.

We agree that physiological variables are typically correlated and that the product of marginals prediction assumes conditional independence across vitals and forecast times. To quantify the implications, we ran an

experiment on the MIMIC test set (22,615 encounters) comparing the standard product of marginals probability against a joint probability computed from autoregressive samples of the same trained ConvCNP, which explicitly capture correlations across vitals and forecast times. Although the ConvCNP is trained with a conditionally independent likelihood, autoregressive sampling — drawing each target point conditioned on previously sampled targets — is a well-established technique for producing coherent correlated samples from NPs trained on marginals [Bruinsma et al., 2023].

The two methods agree closely in practice: mean per-encounter switch-readiness probabilities are 0.0192 (product of marginals) versus 0.0191 (autoregressive joint), with a Spearman rank correlation of 0.86. Downstream ranking performance under the product of marginals is also at least as good, significantly so under a paired bootstrap test, on AUROC (0.882 vs 0.850), average precision (0.481 vs 0.444) and Brier score (0.106 vs 0.108). We attribute this to Monte Carlo noise in the AR estimator: the switch-ready event is rare, so a feasible number of samples gives noisy estimates that shuffle the ordering on which ranking metrics depend, whereas the product of marginals is deterministic. The product of marginals is also orders of magnitude cheaper at inference because running a prediction for N points requires N forward passes through the model vs a single forward pass for the standard case.

We have added a brief mention of this analysis to the Methods alongside the description of the probability computation, and report the full experiment in a new Supplementary section:

> *"We empirically verify that this assumption does not materially affect performance: on the MIMIC test set, probabilities computed this way agree closely (Spearman 0.86) with those from autoregressive samples [Bruinsma et al., 2023], an established technique for drawing coherent correlated samples from NPs trained on marginals, and downstream ranking metrics are at least as good (Supplementary Note 2,* Empirical assessment of the conditional independence assumption*)."*

R2.5 — On the computational cost of NP vs GBDT.

On the same hardware (a single NVIDIA A10 or A40 GPU), training a single NP on the MIMIC dataset takes approximately 25 minutes, with a further 20 minutes for the NP-tuned classification head on top, a STraTS model 1 hour, and a single GBDT 1 minute. We have added a sentence reporting the training times in the Methods alongside the description of the training hardware.

> *"In wall-clock terms, training a single NP on the MIMIC dataset took 25 minutes, with a further 20 minutes for the NP-tuned classification head on top, a STraTS model 1 hour, and a single GBDT 1 minute on this hardware."*

R2.6 — On emphasising the calibration of the NP-tuned variant and on decision curve analysis.

We agree that the calibration difference between the standard NP and the NP-tuned variant is clinically relevant and that the manuscript should be more explicit about recommending the tuned model where probability calibration matters. We have added a sentence to the Discussion, alongside the existing mention of the calibration issue, making this recommendation explicit:

> *"We therefore recommend the NP-tuned variant for any application in which the predicted probability is to be interpreted directly rather than used only as a relative ranking score."*

On decision curve analysis: we agree this is a valuable tool for evaluating clinical net benefit, but it is most informative when the model is used to make specific binary recommendations under a fixed decision threshold. Our system is instead intended to produce a daily ranked list of patients for clinician review, with the final switch decision left to the clinician; the relevant evaluation metrics for this use case are ranking-based (AUROC, AP, precision@5) rather than threshold-based. We therefore consider a full decision curve analysis beyond the scope of this work.

R2.7 — On subgroup analyses by age, sex, ethnicity, and length of stay.

We have computed subgroup-level AUROC, average precision, and Brier score for all five models (Logistic, GBDT, NP, NP-Tuned, STraTS) stratified by age band, gender, ethnicity, and length-of-stay band, on both MIMIC-tight and UCLH. The results are added to the Supplementary Material (Tables 15–22). We do not report precision@5 in this subgroup breakdown because it is a per-day metric defined over the daily ranked list, and most subgroups do not contain enough patients on a given day for the top-5 cut to be a meaningful

summary. Performance is broadly consistent across all stratifications, with the only notable deviation being patients with very short length-of-stay (< 3 days) on MIMIC, who have a much higher positive prevalence (32.7% vs the cohort-wide 12.2%) reflecting the fact that patients discharged very early tend to be well at the prediction time. We have added a sentence to the Results pointing the reader to these tables:

> *"Subgroup analyses stratifying performance by age, gender, ethnicity, and length of stay on both datasets are reported in Supplementary Note 4, and show that performance is broadly consistent across subgroups."*

R2.8 — On patient-level overlap between training and test splits.

Because the test set is constructed by a temporal cut on encounter admission dates, a small minority of patients can appear in both splits via separate admissions falling either side of the cut. We have quantified this on MIMIC and recomputed all test-set metrics on the subset of test encounters whose patient does not appear in training: 8.5% of unique test patients (10.9% of test encounters) overlap with training, and all reported metrics on the disjoint subset are within the bootstrap confidence intervals of the full split for every model. We have added a sentence to the Methods making this explicit and pointed the reader to a new Supplementary section reporting the full analysis (Supplementary Note 3):

> *"Because the temporal split is encounter-based, a small minority of patients can appear in both the training and test splits via separate admissions. On MIMIC this overlap is 10.9% of test encounters. Recomputing the test-set metrics on the disjoint subset of test encounters whose patient does not appear in training leaves all reported metrics unchanged within their bootstrap confidence intervals (Supplementary Note 3)."*

This analysis is restricted to MIMIC because UCLH patient identifiers were not exported alongside the prediction outputs for data governance reasons.

R2.9 — On "switch-readiness" overstating the model's clinical scope.

We retain the "switch-readiness" label because the underlying thresholds are designed to be IVOS-specific (from consultation with the microbiology team) rather than generic stability ranges. To remove the ambiguity the reviewer identifies, we have added short clarifying clauses at the points where a reader is most likely to encounter the term in isolation, stating that "switch-readiness" in this paper refers specifically to the physiological-stability component of the IVOS decision and that oral suitability and other clinical factors remain with the reviewing clinician: the caption of Figure 1, the start of the *Switch-readiness prediction* subsection in the Results, the opening of the *Labelling switches* subsection in the Methods, and the start of the *Switch-readiness probability* subsection in the Supplementary.

R2.10 — On spelling out CDSS at first mention.

We thank the reviewer for catching this. The first occurrence in the manuscript has been expanded to "clinical decision support system (CDSS)", and the redundant expansion on the following line removed.

R2.11 — On streamlining methodological detail in the Results section.

We have kept the methodological detail in the Results section to what we judge to be the minimum needed for the results themselves to be intelligible: in particular, the definitions of the switch-ready label, the ranking-oriented metrics (precision@5, AP), and the task construction are necessary to interpret the numbers in the tables. We would prefer to retain this content in place so that the reader does not need to jump between sections to understand the figures and tables, and hope the resulting balance is acceptable.

R2.12 — On the first paragraph of the Discussion being more like Results material.

We agree that the illustrative encounter (and accompanying figure) reads more as Results material. We have moved this paragraph and the figure to the end of the Results section, and added a brief linking sentence at the start of the Discussion that refers back to the figure and introduces the broader complexities of the IVOS problem that the Discussion goes on to address.

R2.13 — On colour-blind friendly figures.

We have aimed to make the figures legible to readers with colour-blindness by using distinct symbols in each plot in addition to colour, so that series can be distinguished without relying on hue alone. If the reviewer has a specific plot in mind that they find illegible, we would be happy to revise it.

Reviewer 4

Review

Summary: This paper proposes a clinical decision support system framework for IV-to-oral (IVOS) antibiotic switching. The approach decouples physiological forecasting and clinical decision-making, using neural processes (NP) to model the former, and existing clinical criteria to estimate switch-readiness probabilities and rank patients for review. The model was constructed and validated retrospectively on MIMIC-IV (ICU, USA), and UCLH (general hospital, UK). NP physiological forecasting moderately outperforms reference models including gradient boosted decision trees but yields forecasted vital signs that can be used to prioritize patients for transition from IV to Oral antibiotics.

Strengths:

- The main strength of the paper is the separation of vital sign forecasting from decision support, avoiding learning and labeling issues associated with historical prescribing actions.
- Neural processes are an appropriate approach given their ability to provide uncertainty quantification around forecasted values.
- Multi-setting validation (ICU and general ward)

Critiques:

- No comparison against a simple, interpretable rule-based IVOS alert system, which is prevalent in stewardship programs and directly relevant to the paper's claims.
- Model baselines are limited (repeat, GBDT); no comparison to strong modern irregular multivariate time series (IMTS) models (e.g., GRU-D, TFT/OmniTFT, STraTS, latent ODE/SDE); also a comparison or consideration with respect to reinforcement learning strategies
- Probabilistic forecasting is only assessed by point MAE; proper scoring rules (continuous ranked probability score, negative log-likelihood) and coverage of predictive intervals are not reported. These metrics would substantiate the quality of uncertainty used in the criteria step.
- Limited handling of patient covariates (conditioning on vital signs only, what about comorbidities, etc?) Did you assess subgroup performance (e.g., ventilated vs non-ventilated, oxygen therapy, age/sex/ethnicity)? Are there contexts where excluding certain vitals systematically improves specificity?
- The computation of switch-readiness as a product of per-time, per-variable probabilities assumes conditional independence across vitals and times; this can miscalibrate joint event probabilities.
- The MIMIC temporal split relies on reconstructed dates; although noted, this can affect precision@K and should be probed with additional leakage-controlled splits (e.g., subject-level).

Questions:

- How was the cohort constructed? Only looking at patients who were on antibiotics? If so it should be included in the data description.
- How were clinical thresholds chosen relative to published IVOS guidance (e.g., UK IVOS/NEWS2), and would you consider site-specific threshold learning or adaptive policies?
- Can these scores provide any insight as to the appropriateness of prior actions? For example, can this retrospectively identify cases in which switching was not appropriate in terms of downstream interventions or outcomes?
- How exactly are joint switch-readiness probabilities computed in deployment: by multiplying marginal probabilities at interval centers (see critique) or by sampling full trajectories? If the former, have you quantified the bias introduced by the independence assumption?

Suggestions:

- The clinical thresholds for "normal ranges" are referenced to the Supplementary; brief justification and alignment with recognized guidance (e.g., UK IVOS/NEWS2) in the main text would help.
- Beyond precision@5, how does precision/recall vary with K (review capacity)? Could you include cumulative gain or decision-curve analyses to guide site-specific deployment?

Minor comments: In multiple tables, "bold indicates best (p 0.05)" appears to invert the usual significance convention (likely p 0.05).

Acronyms and References: GRU-D: Che, Z., Purushotham, S., Cho, K. et al. Recurrent Neural Networks for Multivariate Time Series with Missing Values. Sci Rep 8, 6085 (2018). https://doi.org/10.1038/s41598-018-24271-9

TFT: Lim, B., Arik, S., Loeff, N., Pfister, T. Temporal fusion transformers for interpretable multi-horizon time series forecasting. International Journal of Forecasting 37:4 (2021). https://doi.org/10.1016/j.ijforecast.2021.03.012

TFT-Multi/OmniTFT: He, R., Chiang, J.N. Simultaneous forecasting of vital sign trajectories in the ICU. Sci Rep 15, 14996 (2025). https://doi.org/10.1038/s41598-025-99719-w

Xu, W., Dai, Y., Yang Y., Loza M., et al. OmniTFT: Omni-target forecasting for vital signs and laboratory result trajectories in multi center ICU data. arXiv (2025). https://doi.org/10.48550/arXiv.2511.19485

STraTS: Tipirnene, S., Reddy, C., Self-supervised transformer for sparse and irregularly sampled multivariate clinical time-series. ACM Transactions on Knowledge Discovery from Data (TKDD) 16:6 (2022). https://doi.org/10.1145/3516367

Our responses

We thank the reviewer for their careful and constructive review, and for highlighting the strengths of the approach — in particular the separation of physiological forecasting from decision support, the use of neural processes for uncertainty quantification, and the multi-setting validation. We respond to each point in turn below (first critiques, then questions).

R4.c1 — On comparison against a simple, interpretable rule-based IVOS alert system.

This comparison is in fact already present: the "Repeat" row in Table 4 applies the same fixed-threshold IVOS criteria used to construct the switch-readiness label directly to the most recent measured value of each vital sign, with no forecasting, and is equivalent to a simple rule-based alert system. The Repeat / rule-based approach is necessarily absent from the ranking table (Table 3) because it produces only a binary indicator, an inability to support prioritisation that is itself a limitation of fixed-threshold alerts.

R4.c2 — On comparison against modern irregular multivariate time series (IMTS) models and reinforcement learning.

We have added STraTS [Tipirneni & Reddy, 2022] as a representative modern IMTS baseline, trained on the same daily switch-readiness tasks as the other classifiers. Results are now in Tables 3 and 4, the subgroup analyses (R2.7), the patient-overlap analysis (R2.8), and the precision@K curve (R4.s2); implementation details are in the updated Methods.

STraTS performs competitively: it achieves a small but significant improvement over the NP on AUROC on both datasets (UCLH 0.870 vs 0.858; MIMIC 0.886 vs 0.882) and on average precision on UCLH (0.805 vs 0.785), while on MIMIC the average precision of STraTS, NP and GBDT is statistically indistinguishable. On precision@5, the metric most aligned with deployment, STraTS and the NP are statistically tied on both datasets, and on the binary metrics STraTS ties with or marginally exceeds the NP. We read this as positive evidence for the paper's central claim: the system (forecast-and-apply-criteria with a clinician-facing ranked list) is not tied to neural processes specifically and works across modern architectures. Crucially, STraTS outputs only a switch-readiness probability and cannot be queried for individual vitals at arbitrary times, so it cannot expose the per-variable forecasts central to clinician trust and to the deployment-time adjustments described in the Discussion (e.g. discounting respiratory rate for a ventilated patient). The Discussion has been updated to make this contrast explicit.

We chose STraTS as a representative of the IMTS family because it is purpose-built for sparse, irregularly sampled clinical time series and performs strongly in the benchmarks the reviewer points to; an exhaustive sweep over GRU-D, TFT/OmniTFT and latent ODE/SDE would substantially enlarge the paper without changing the methodological claim. With regards to reinforcement learning an RL formulation would require either a reward derived from downstream outcomes (inheriting the historical-decision biases we explicitly avoid, see R1.1) or curated ground-truth "correct switching" labels, neither of which is available at scale in MIMIC-IV or UCLH. We agree this is an interesting future direction but consider it out of scope here.

R4.c3 — On proper scoring rules and coverage of predictive intervals for the probabilistic forecasts.

We have evaluated the NP under the continuous ranked probability score (CRPS), a strictly proper scoring rule that integrates over the full predictive distribution. For the deterministic Repeat and GBDT baselines CRPS reduces exactly to MAE, so the values already in Table 2 provide the appropriate reference. Against this reference the NP's CRPS sits substantially below the baselines' MAEs across all five vital signs on the MIMIC test set (e.g. pulse 6.05 vs GBDT 8.92, systolic BP 8.60 vs 12.27), confirming the NP's probabilistic forecasts are meaningfully more informative than the baselines' point predictions under a proper scoring rule. Full results are in Supplementary Note 2 (*CRPS evaluation*) and a short pointer has been added to the Results.

R4.c4 — On patient covariates, subgroup performance, and excluding vitals.

Subgroup analyses by age, gender, ethnicity, and length-of-stay on both datasets have been added to the Supplementary; see R2.7 for the full set of tables. Performance is broadly consistent across subgroups. The specific case of excluding a vital sign for a relevant patient context (e.g. discounting respiratory rate for ventilated patients to improve specificity) is addressed by a new deployment-time ablation; see R1.6 for the experiment and the new Supplementary section. On the broader question of conditioning on additional static covariates (comorbidities, etc.), we deliberately kept the model inputs restricted to routinely measured vital signs to keep the system simple and broadly deployable; extending to static covariates is an interesting direction for future work.

R4.c5 — On the conditional independence assumption in switch-readiness probability.

We have quantified the empirical impact of this assumption by comparing the product-of-marginals against an autoregressive joint estimator on the MIMIC test set; the per-encounter agreement and downstream ranking metrics are reported in a new Supplementary section. See R2.4 for the full discussion.

R4.c6 — On the MIMIC reconstructed-date split and subject-level leakage controls.

We have re-evaluated all test-set discrimination metrics on the disjoint subset of MIMIC test encounters whose patient does not appear in the training split (10.9% of test encounters overlap at the patient level); all metrics on the disjoint subset are within the bootstrap confidence intervals of the full split. See R2.8 for the full analysis and the new Supplementary section.

Questions

R4.q1 — On cohort construction.

Yes, the cohort is restricted to encounters with at least one IV antibiotic prescription. This was implicit in the task-construction rule in the *Data* subsection of the Methods (a task is only created on a day when the patient is on an IV prescription, so encounters with no IV prescription contribute no tasks), but we agree it was not stated at the cohort level. We have added a sentence to the inclusion/exclusion criteria in the *Data* subsection making this explicit.

R4.q2 — On choice of clinical thresholds and site-specific / adaptive policies.

The thresholds were developed in consultation with the microbiology team at our institution, using the National Early Warning Score 2 (NEWS-2) thresholds as a starting reference (the score 1 level for each variable), and held fixed across both datasets. The MIMIC-loose sensitivity analysis already in the manuscript (positive class rises from 12.2% to 50.0%) shows the relative ordering of the top models is essentially unchanged under substantially different thresholds (Supplementary Note 2, *Criteria threshold sensitivity*). Because the system

decouples forecasting from criteria, a deploying site can substitute its own thresholds without retraining the forecasting model; principled adaptive or learned thresholds are an interesting direction for future work.

R4.q3 — On using the scores to retrospectively assess the appropriateness of prior switching decisions.

In principle the ranked switch-readiness probabilities could be used to flag historical cases in which the model would have indicated readiness well before an actual switch (or where no switch occurred despite sustained readiness), and similar retrospective audits of antibiotic prescribing have been performed in prior work [Dutey-Magni et al., 2021]. We did not pursue this analysis here because it requires switch labels and these are not consistently captured in either dataset. We agree this is a valuable direction for future work.

R4.q4 — On the computation of joint switch-readiness probabilities and the bias of the independence assumption.

The product-of-marginals computation is described in the *Neural processes* subsection of the Methods and the *Further neural process details* subsection of the Supplementary Methods, and the empirical bias of the independence assumption is addressed in responses R2.4 / R4.c5.

Suggestions

R4.s1 — On brief justification of clinical thresholds and alignment with recognized guidance.

We have added a short clause to the *Labelling switches* subsection of the Methods clarifying that the thresholds were anchored to the NEWS-2 score-1 boundaries, with the final levels set in consultation with the microbiology team at our institution.

R4.s2 — On precision/recall as a function of K and decision-curve / cumulative-gain analyses.

We have added a precision@K analysis for $K \in \{1, 2, 3, 5, 10\}$ on the MIMIC test set, with a per-day oracle for context (Supplementary Note 2, *Precision@K across review-capacity values*); a pointer has been added to the Results. The relative ordering of models is essentially stable across K. On decision-curve analysis: as noted in R2.6, our system is intended to produce a ranked list of patients for review rather than a binary recommendation under a fixed threshold, so the relevant evaluation metrics are ranking-based; we therefore consider a full decision-curve analysis beyond the scope of this work.

Minor comments

R4.m1 — On the significance convention in table captions ("bold indicates best (p $\geq$ 0.05)" appearing inverted).

We thank the reviewer for flagging the ambiguity. The intended meaning is that bold marks the top-performing model on each metric *together with any models not significantly worse than it* under a paired bootstrap test, so the $p \geq 0.05$ threshold is in fact the correct direction (it identifies models statistically indistinguishable from the best). The full procedure, including the Bonferroni correction for multiple comparisons, is described in the Methods (*Statistical significance testing*). We have revised the captions of Tables 2, 3 and 4 in the main text, and Supplementary Tables 8 and 9, to spell this out and to point the reader to the significance-testing section, so the convention is no longer ambiguous.

Response to Reviewers (Second Round)

We thank the editor and reviewers for the further consideration of our manuscript "Optimising antibiotic switching via forecasting of patient physiology". We are pleased that Reviewers 2 and 4 have no further concerns. Reviewer 1 raised two additional points, both clinical in nature, which we address below. As before, we reproduce the review verbatim and then give point-by-point responses. In the manuscript we highlight changes from this revision in blue, with changes from the previous revisions remaining in green.

Reviewer 1

Review

I read with interest the revised version of the retrospective study by Magnus Ross and colleagues entitled "Optimising antibiotic switching via forecasting of patent physiology". Please find below some comments on its technical merits.

Minor comments

1) The authors should be commended for their comprehensive responses to comments and for the detailed additional analyses they provided in the revised version. In my opinion, what should be further highlighted, as a limitation, is the proof-of concept nature of the analysis and the absolute need for prospective clinical validation. Please note that this also deeply involves the additional considerations expressed by the authors in the revised version. For example, it is now (correctly) stated that "switch-readiness refers specifically to the physiological-stability component of the IVOS decision (vital signs within criteria); oral suitability and other clinical factors remain with the reviewing clinician". The fact is that, in clinical practice, vital signs are often exactly the portion of the assessment that is very quick and easy for experts of IVOS. Consequently, it is also possible that the issue "senior review, especially around discharge planning, microbiology and stewardship input typically push prescribing towards IVOS, but these are finite resources that cannot reach every eligible patient when they are ready, delaying the switch" could not be resolved. Indeed, in clinical practice, clinicians usually check HR, RR, SpO2, SBP, and Temp every day for all patients in their ward very rapidly, also usually and easily remembering the trajectory of vital signs in the previous days (to support these statements, it should be acknowledged that I am a clinician). In those few seconds, they may have already inherently prioritized patients for IVOS and other clinically relevant tasks (and, without efforts, they also have automatically not considered RR in mechanically ventilated patients, etc.). The relevant and time-demanding work is then that of reflecting on suitability of IVOS (availability of oral options, activity of oral options against the causative agent/s, expected patients' adherence to treatment, etc.), which is not addressed by the model. I do not mean that a model should substitute clinical judgment, but that it is possible that the proposed advantage may not be automatically present in real-world practice because it would substitute an already easy and quick task. Although I recognize that this could not be true everywhere, this is nonetheless why I'd suggest to further stress in the limitations that the performance of the model should be compared with classical approaches though appropriate prospective clinical studies (preferably RCT), to confirm whether or not a true advantage is present (e.g., what if the time to review false positives proves longer than the already quick classical approaches, in the absence of substantial advantages? On this aspect, see also the next comment) or where is present (as the efficacy of standard approaches may vary across centers)

2) The other major limitation that supports, in my opinion, the need for caution and further assessment in clinical studies is the lack of microbiological information. I recognize that now the authors state that "the limited availability of microbiology results in both datasets precluded subgroup analyses by proven infection, infection site, or antibiotic appropriateness. Because of this, it is possible that patients in the evaluation set did not have a bacterial infection at all, and it is therefore not established that forecasting performance is consistent across patients with proven infection and those without", but this does not cancel that this major limitation remains and it might have important conceptual implications. For example, without proper subgroup analysis for confirmed infection, how one can be sure that the model is not improving switching where not needed (unconfirmed infections that could conversely benefit from a re-assessment of whether or not they truly are infections) and that this contributes relevant noise to the hypothetical benefit?

Our responses

We thank the reviewer for these two further comments. We address them below.

R1.1 — On vital-sign assessment being the quick part of the IVOS decision, and the consequent need for prospective evaluation.

We agree with the reviewer's clinical observation that, for a single patient, checking vital signs against criteria is quick, and that the more time-consuming work of judging oral suitability (tolerance, availability of oral

alternatives, likely adherence etc.) is not addressed by the model and remains entirely with the clinician. We would make two points in response.

First, the model is doing non-trivial forecasting rather than re-presenting the current values: the "Repeat" baseline applies the criteria directly to the latest measurement (essentially the snapshot a clinician reads at the bedside) and is significantly outperformed by the forecasting models on both datasets, indicating that there is information in the vital-sign trajectory beyond a point-in-time check.

Second, our claim is not that the system speeds up the per-patient assessment necessarily but that it provides a prioritisation function. While identifying an improving patient is easy for a clinician reviewing a single case, reliably performing that assessment across every eligible inpatient each day is not. This is reflected in the persistent under-switching that motivates the work (e.g. 19% of patients in England remaining on IV antibiotics despite meeting the criteria). The system is designed for the setting in which there is not enough capacity to review every patient, allowing limited review effort to be directed at those most likely to be ready.

We fully agree with the reviewer that whether this prioritisation confers a real-world advantage can only be established by prospective, ideally randomised, comparison against standard practice. We have made this more explicit in the Discussion:

> *"Such an evaluation would also need to establish the value of the prioritisation itself: because assessing a single patient against the criteria is already quick, any benefit must instead come from directing finite review capacity across every eligible inpatient, which retrospective metrics cannot demonstrate."*

R1.2 — On the lack of microbiological confirmation

We accept that, without microbiological confirmation, we cannot be certain every patient in the cohort had a bacterial infection. We would note, however, that in most hospitals the majority of patients receiving IV antibiotics never have a microbiologically confirmed infection, while IVOS decisions must still be made for them daily; restricting to confirmed infection would exclude much of the population in which the decision is actually faced. Prior ML work on IVOS likewise does not restrict to microbiologically confirmed cases [Bolton et al., 2024].

More importantly, we would suggest that surfacing such patients prompts useful clinical review rather than introducing noise. The system flags physiological stability and leaves the resulting action to the clinician; for a patient who may not have a bacterial infection, that same review is a natural opportunity to reassess whether antimicrobials are needed at all. That being said, we have not evaluated the system as a discontinuation tool, and our results concern ranking for switch-readiness only.

We have added the following to the discussion to reflect these points.

> *"We note, however, that most patients receiving IV antibiotics never have a microbiologically confirmed infection, while IVOS decisions must still be made for them. Because the system flags physiological stability and leaves the resulting action to the clinician, surfacing such patients still prompts a review that may identify either a switch or, where appropriate, a reassessment of the need for antimicrobials."*

Reviewers 2 and 4

We thank Reviewers 2 and 4 for confirming that their previous comments have been satisfactorily addressed. We have no further changes to make in response to these reports.
